# 2-Aminothiazole-Flavonoid Hybrid Derivatives Binding to Tau Protein and Responsible for Antitumor Activity in Glioblastoma

**DOI:** 10.3390/ijms242015050

**Published:** 2023-10-10

**Authors:** Rayane Hedna, Attilio DiMaio, Maxime Robin, Diane Allegro, Mario Tatoni, Vincent Peyrot, Pascale Barbier, Hervé Kovacic, Gilles Breuzard

**Affiliations:** 1 Faculté des Sciences Médicales et Paramédicales, Institut de Neurophysiopathologie (INP), UMR 7051, CNRS, Aix Marseille Université, 13005 Marseille, France; rayane.hedna@etu.univ-amu.fr (R.H.); diane.allegro@univ-amu.fr (D.A.); mario.tatoni@univ-amu.fr (M.T.); vincent.peyrot@univ-amu.fr (V.P.); pascale.barbier@univ-amu.fr (P.B.); herve.kovacic@univ-amu.fr (H.K.); 2 Faculté de Pharmacie, Institut Méditerranéen de Biodiversité et Ecologie Marine et Continentale (IMBE), UMR 7263, CNRS, IRD 237, Aix-Marseille Université, 13005 Marseille, France; attilio.di-maio@etu.univ-amu.fr (A.D.); maxime.robin@univ-amu.fr (M.R.)

**Keywords:** 2-aminothiazole, flavonoid, microtubule-associated protein Tau, glioblastoma, medicinal chemistry

## Abstract

Tau protein has been described for several decades as a promoter of tubulin assembly into microtubules. Dysregulation or alterations in Tau expression have been related to various brain cancers, including the highly aggressive and lethal brain tumor glioblastoma multiform (GBM). In this respect, Tau holds significant promise as a target for the development of novel therapies. Here, we examined the structure–activity relationship of a new series of seventeen 2-aminothiazole-fused to flavonoid hybrid compounds (TZF) on Tau binding, Tau fibrillation, and cellular effects on Tau-expressing cancer cells. By spectrofluorometric approach, we found that two compounds, **2** and **9**, demonstrated high affinity for Tau and exhibited a strong propensity to inhibit Tau fibrillation. Then, the biological activity of these compounds was evaluated on several Tau-expressing cells derived from glioblastoma. The two lead compounds displayed a high anti-metabolic activity on cells related to an increased fission of the mitochondria network. Moreover, we showed that both compounds induced microtubule bundling within newly formed neurite-like protrusions, as well as with defection of cell migration. Taken together, our results provide a strong experimental basis to develop new potent molecules targeting Tau-expressing cancer cells, such as GBM.

## 1. Introduction

In eukaryotic cells, the microtubule cytoskeleton constitutes a functional network essential for cell division, motility, intracellular trafficking of organelles and macromolecules, and cell morphogenesis. The primary significance of microtubules stems from their versatile and remarkable dynamicity. This dynamic instability is characterized by rapid stochastic transitions between growth and shortening, which result from the association and dissociation of αβ-tubulin protein dimers with their ends [1]. Given their crucial role in biological processes, microtubules have become a preferred target for numerous compounds, commonly referred to as ‘antimitotic agents’ in chemotherapy. 

Thiazole, a five-membered heterocyclic ligand containing sulfur and nitrogen atoms, serves as an essential core scaffold in many medicinally important compounds that include but are not limited to clinically available anticancer compounds such as tiazofurin (an inhibitor of inosine 5′-monophosphate dehydrogenase, a key factor in de novo GTP biosynthesis) [2], dasatinib (a Bcr-Abl tyrosine kinase inhibitor) [3], dabrafenib (an inhibitor of enzyme B-Raf) [4], and patellamide D (cytotoxicity against multidrug-resistant cancer) [5] (for more details, refer to [6]). More interestingly, it has been demonstrated that the thiazole-containing drugs like epothilone and its derivatives possess noteworthy microtubule inhibitory properties [7,8]. However, the administration of these compounds to patients remains ineffective in the treatment of many cancers, including the highly aggressive and lethal brain tumor glioblastoma multiform (GBM) [9,10,11]. These therapeutic failures can be attributed to multiple factors, including the emergence of tumor resistance to antimitotic agents. In order to overcome these challenges, an alternative approach could involve the development of hybrid compounds which fuse the thiazole scaffold with new drugs targeting microtubule-associated proteins, such as Tau protein. 

Tau promotes the assembly and stabilization of microtubules [12,13,14]. In adults, there are six predominant Tau isoforms, resulting from alternative splicing of mRNA of a single gene *MAPT*, each with three or four microtubule-binding repeats located in the C-terminal half of the protein, and with zero to two inserts located in the N-terminal portion [15]. Tau protein expression was initially reported as being physiologically restricted to neurons, in which its presence under hyperphosphorylated and aggregated states is toxic [16,17]. Subsequently, growing evidence has unveiled the role of Tau overexpression in a variety of cancers such as GBM [18,19]. Additionally, we recently demonstrated that Tau contributes significantly to the invasiveness and proliferation of GBM-derived U87 cells [20,21]. We further argued that Tau protein could represent a new interesting anticancer target in GBM [22]. Interestingly, molecules developed for Tau-related neurodegenerative diseases could display anticancer potency, especially compounds containing the thiazole group (see review of [23]). However, most studies describing new drugs focused on the disruption of cancer-related kinase proteins, not Tau protein itself [24,25,26,27]. The development of new hybrid compounds specific to Tau protein is therefore a more attractive approach to directly affect microtubules. In this regard, flavonoid derivatives, including flavonol and flavone, constitute a substantial class of naturally occurring compounds with diverse pharmacological properties such as antitumor, antibacterial, immune-stimulator, antifungal, and antidiabetic activities in addition to anti-Tau fibrils properties [28,29,30,31,32,33]. For example, Sonawane et al. recently showed in vitro that Baicalein inhibited formation of Tau fibrils by enhancing the formation of SDS-stable oligomers [34]. More interestingly, the authors also found using far-UV circular dichroism spectroscopy that this compound induces some partial folding in the β-sheet structure of Tau. Using atomic force microscopy and fluorescence microscopy, Kumar et al. demonstrated that quercetin inhibits Tau fibrillation [35]. Altogether, these results strongly suggest that some flavonoids can bind to Tau protein as well as maintain Tau in a soluble form. 

**Figure 1:**
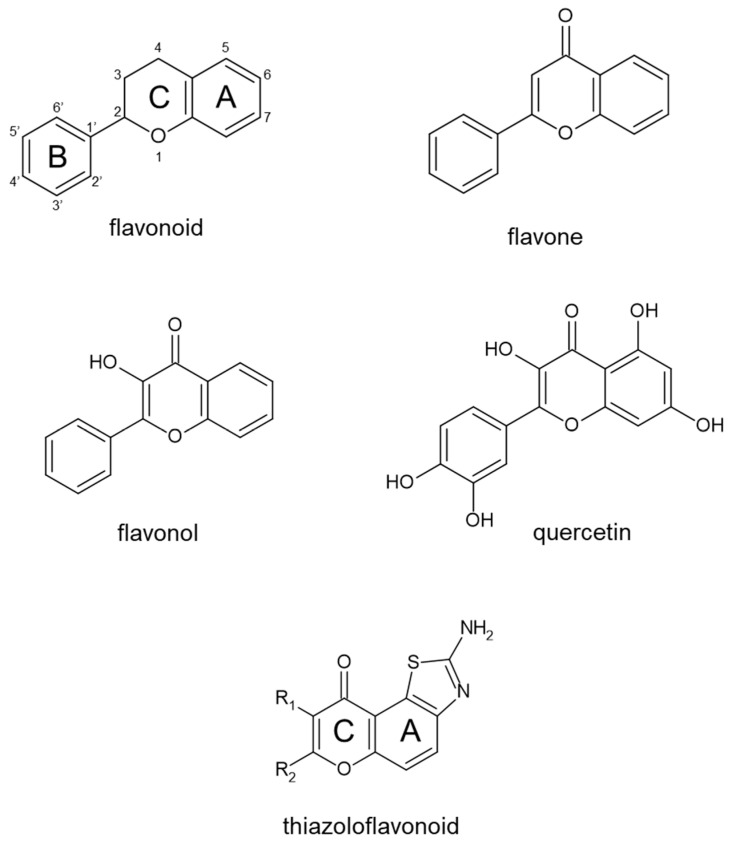
Basic chemical structure of the flavonoids, flavones, flavonols, quercetin, and thiazoloflavonoid derivatives investigated here.

Given the specific interaction of some flavonoids with Tau as well as the anticancer benefits of compounds with the thiazole group, we examined the structure–activity relationship of new seventeen 2-aminothiazole-flavonoid (TZF) hybrid derivatives (Figure 1; patent No: WO2016083490A1), and then we further explored their mechanism of action in cells. The resulting compounds underwent in vitro assessment to determine their binding constants to Tau protein and their ability to dissolve Tau aggregates. We then examined their anti-metabolic activity on multiple cell models expressing or not the Tau protein and including four GBM-derived cells among other models [20]. Two lead compounds, **2** and **9**, displayed Tau-dependent biological effects towards microtubule network remodeling in cells. Our findings revealed the potential of Tau-targeting TZF for the treatment of GBM and other cancers characterized by Tau overexpression. Lastly, we proposed a binding model for the interaction of the biologically active compounds with Tau.

## 2. Results

### 2.1. Thiazoloflavonoids Inhibit Human Full-Length Tau Aggregation into Filaments

The fluorescence emission measurement of the fibrillary cross-β-sheet dye, thioflavin S (ThS), was employed to assess the extent of Tau filaments formation, as previously described [36,37]. An in vitro ThS fluorescence assay was performed with two main objectives: (1) to screen compounds with inhibitory effects on the assembly of Tau filaments, and (2) to determine those capable of dissolving mature Tau filaments. As a preliminary step, we monitored the progress of heparin-induced assembly of Tau filaments in solution by measuring ThS fluorescence emission every hour for 15 h (Figure S1 in Supplementary Materials File S2). The fluorescence intensity increased for the untreated Tau up till 8 h and then remained stable, validating Tau aggregation into β-sheet structure.

**Figure 2:**
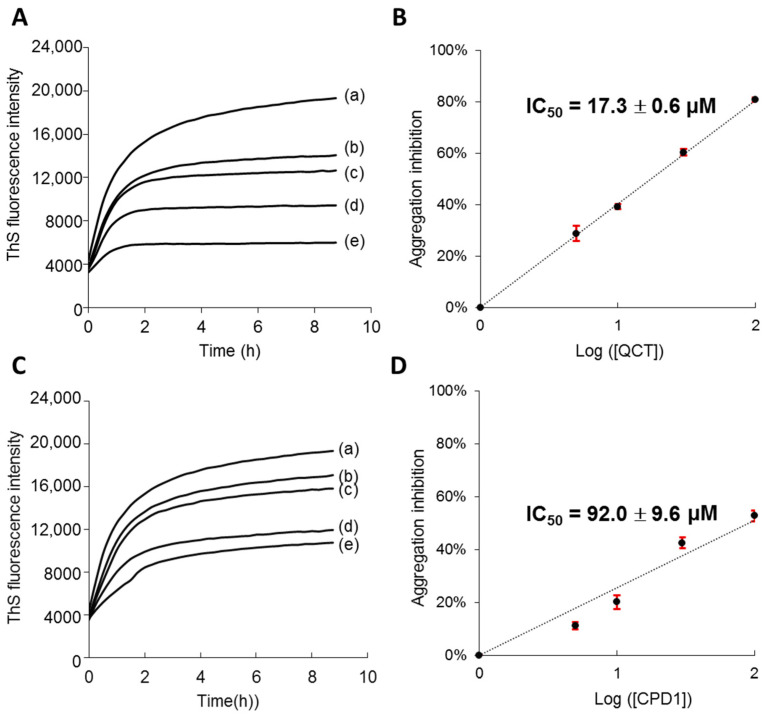
Inhibitory effects of quercetin (QCT) and compound (**1**) on the heparin-induced aggregation of Tau. Time courses of the aggregates formation were performed in the presence of thioflavine S (ThS) at 37 °C. The variation of ThS fluorescence emission vs. time was recorded at 520 nm by spectrofluorometry (with excitation at 440 nm). Panels (**A**,**C**) show (curve a) Tau at 2 µM and (curves b–e) aliquots of the same solution with 5 µM (b), 10 µM (c), 30 µM (d), 100 µM (e) of QCT (panel (**A**)) and compound (**1**) (Panel (**C**)); Panels (**B**,**D**) show the percentage of aggregation inhibition as a function of the ratio of the total drug concentration to total Tau concentration: QCT (Panel (**B**)), compound (**1**) (Panel (**D**)); ‘IC_50_’ values in graphs represent the calculated concentration of compound reducing of 50% of Tau aggregates (in µM).

Next, time courses of Tau assembly were performed with quercetin (QCT), as well as 2-aminothiazole-fused chromen-4-one derivatives **2**–**17** and one compound in which the thiazole group was maintained detached from the A ring (compound **1**) (0–100 µM). For QCT (Figure 2A,B), the rate of Tau assembly as well as the final quantity of aggregates were lower in the presence of the drug (curves (b–e), in panel A) compared to untreated Tau (curve (a) corresponding to 2 µM Tau without ligand). Our data suggest an efficient inhibition of Tau assembly by QCT at very low concentrations. Moreover, as shown in Figure 2B, the extent of inhibition increased linearly with the ratio of the total ligand concentration to total Tau concentration until a plateau value corresponding to a full inhibition of the filament formation was reached. Specifically, 17.3 µM of QCT was necessary to halve the Tau assembly (see Table 1). In the presence of compound **1**, 50% inhibition occurred at 92.0 µM of drugs (Figure 2C,D and Table 1). Our data indicate that compound **1** has a moderate effect on inhibition of Tau fibrillation in comparison with QCT, which could be due to lower binding affinity constants to Tau. However, our data suggest for both compounds an inhibition of Tau assembly by the binding of either a Tau–drug complex or a drug molecule to the growing Tau oligomer.

Various ring systems that are bio-isosteric to the phenyl ring B such as (**1**) were introduced to the C ring, yielding in compounds **2**–**5** (Table 1). In this first series, the 2-aminothiazole group was fused to the A ring side on chromen-4-one heterocycle. As a result, the inhibitory effect on Tau assembly was lost for compound **3**, as well as for compound **5**, where a carboxyl group substituted ring B (Table 1). On the other hand, compound **2**, with a diphenyl ether group, exhibited threefold greater potency in inhibiting filaments formation compared to compound **1**. Furthermore, **4**, displaying an ethyl benzoate group, emerged as the most active compound with 50% inhibition occurring at 9.7 µM of drugs. To complete this series, compound **16**, in which O1 and C3 in ring C were substituted by nitrogen atoms, displayed inhibitory activity threefold higher compared to analog 1. Surprisingly, the structural analogue of 16, compound **17,** did not elicit potent activity on Tau fibrillation.

Prompted by our previously reported high antitumor activities of new 4-arylcoumarin analogues with fluorine residues [38], we designed a second series of compounds (**6**–**15**) in which fluorine was added in ortho (**6**, **9**, **13**), meta (**7**, **10**, **14**), or para (**8**, **11**, **15**) on the ring B coupled to H- (**6**–**8**), OH- (**9**–**11**), or OCH3- (**13**–**15**) on R1 (Table 1). Most of these compounds demonstrated high efficacy in inhibiting Tau assembly at µM concentrations, except for compound **11** which did not have any effect. Therefore, our data strongly suggest that fluorine residue on the phenyl ring B could bring a substantial inhibitory effect on Tau assembly.

**Figure 3:**
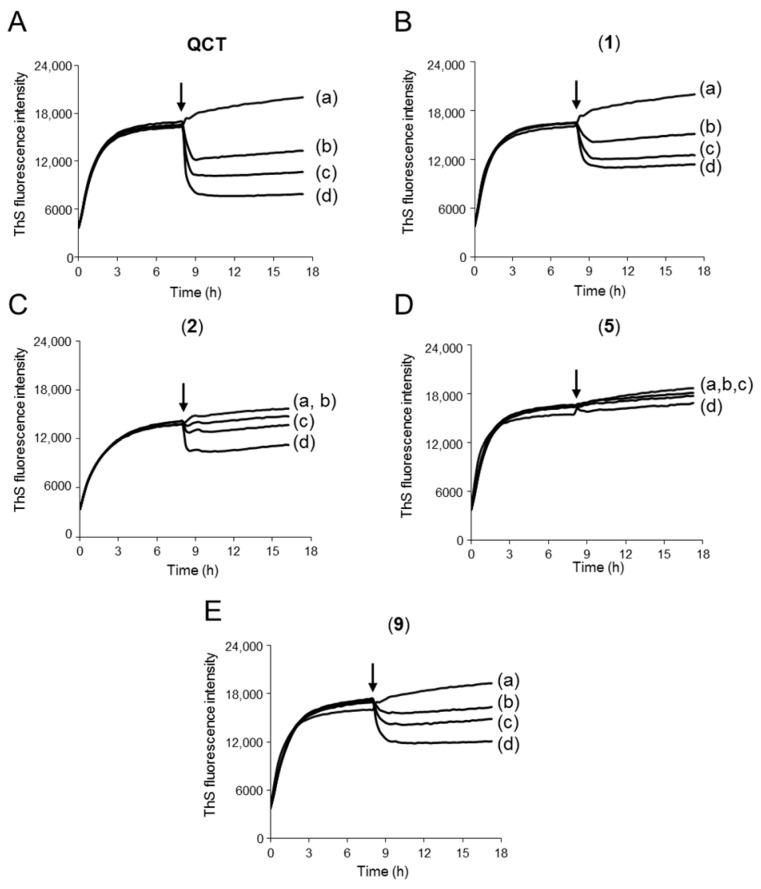
Compounds QCT (panel (**A**)), (**1**) (panel (**B**)), (**2**) (panel (**C**)), and (**9**) (panel (**E**)) were able to dissolve mature Tau aggregates, not compound (**5**) (panel (**D**)). Eight-hour time courses were performed with ThS to monitor heparin-induced Tau aggregation by spectrofluorometry at 520 nm (37 °C), then 10, 30, and 100 µM of compounds (curves b–d) were added to the ThS-labelled Tau aggregates. A control was performed without compound (curve a).

Moreover, we performed a qualitative study of the ability of QCT and compounds **1, 5**, and **9** to dissolve preformed Tau aggregates (Figure 3). The outcomes revealed that the ThS fluorescence kinetics demonstrated a drop in intensity over time in QCT-, **1**-**,** and **9**-treated reactions (curves b-c) as opposed to control, which showed steady fluorescence throughout the incubation (curve a). As expected, no significant change of ThS fluorescence intensity was measured for **5**-treated Tau filaments, indicating the inability of compound **5** to dissolve Tau fibrils. 

In this new series of TZF derivatives, many compounds exhibit notable potency in inhibiting Tau assembly as well as dissolving the preformed filaments. Furthermore, we also demonstrated that a thiazole nucleus fused to chromen-4-one heterocycle can bring a superior inhibitory effect of Tau assembly compared to when it is detached, as is the case for compounds **2** and **9**. Altogether, our results indicate a potent interaction of compounds to Tau protein. In the next step, we measured the binding kinetic parameters of these compounds to examine their interaction mode with Tau protein.

### 2.2. Kinetics and Mechanism of Thiazoloflavonoids Binding to Tau Protein

Fluorescence spectroscopy is an invaluable tool to investigate interactions between proteins and ligands, including quenching mechanism, and conformational change within protein in the presence of small molecules [39,40]. To determine their binding mechanism, the binding constants of all compounds were calculated. The existing studies show that the intrinsic fluorescence of full-length Tau protein mainly comes from five tyrosine (Tyr8, Tyr29, Tyr197, Tyr310, and Tyr394) and three phenylalanine (Phe8, Phe346, and Phe378). Note that no tryptophan figures in the protein sequence. Moreover, Phe residues are not excited due to low quantum yield. In contrast, Tyr residues have the strongest fluorescence intensity at the maximum emission wavelength of 303 nm and are most sensitive to microenvironment changes, which indicate conformational changes upon compound binding [41]. In our experimental conditions, Tau quenching was monitored between 280 and 500 nm, with an excitation at 275 nm. Figure S2 (in Supplementary Materials File S2) exhibits the fluorescence spectra of the Tau–compound systems. The Tau fluorescence emission gradually decreased at 303 nm with the increasing concentrations of compounds (QCT, (**1**–**17**)), indicating that these small molecules approached the Tyr residues of Tau near the binding site. These changes suggest that QCT can quench the inner fluorescence of Tau and clearly illustrate the interactions between the ligands.

When plotting the steady-state quenching data using the regular Stern–Volmer equation, no linear relationship was obtained. Such behavior may be caused by differences in the accessibility of tyrosine residues. To understand the fluorescence quenching mechanism of Tau with the compounds, we used the modified Stern–Volmer plot (Equation (8)) to analyze the entire static quenching process. Binding parameters (the compound equilibrium dissociation constant (K_D_) and the Tyr accessible fraction (f_A_)) are summarized in Table 1 (right columns). The results of steady-state fluorescence experiments did indeed show a strong compound dependence: for QCT, the equilibrium dissociation constant was determined to be 2.4 ± 0.6 × 10^−6^ M with f_A_ = 0.12 ± 0.04, but for compound **1**, it was substantially higher, 7.0 ± 2.3 × 10^−6^ M with f_A_ = 0.39 ± 0.08. The fact that QCT has both a lower dissociation constant and a lower accessible fraction value indicates a higher embedding in the Tau protein than compound **1**.

Moreover, we calculated low dissociation constants and low accessible fractions for compounds **2** and **4** (respectively, K_D_ = 4.7 ± 1.8 × 10^−6^ M with f_A_ = 0.14 ± 0.10; and K_D_ = 2.4 ± 0.8 × 10^−6^ M with f_A_ = 0.12 ± 0.01). Similarly, compounds **16** and **17** also showed low dissociation constant (respectively, K_D_ = 5.0 ± 2.5 × 10^−6^ M with f_A_ = 0.20 ± 0.03; and K_D_ = 3.5 ± 1.0 × 10^−6^ M with f_A_ = 0.14 ± 0.05). Both low K_D_ and f_A_ values were also calculated for the series of fluorinated TZFs (**7**, **9**–**15**) (see Table 1). Our data indicated similar binding modes with a low accessible fraction in Tau protein for all these ligands. In contrast, compound **5** displayed an equilibrium dissociation constant of 6.4 ± 1.5 × 10^−6^ M with f_A_ = 0.38 ± 0.01, indicating low embedding of ligand in Tau protein. Note that for the tested compounds **3**, **6**, and **8**, a modified Stern–Volmer equation resulted in a nonlinear plot, precluding the calculation of binding kinetic parameters. This may indicate a nonpolar binding mechanism for these ligands.

In our study, the modified Stern–Volmer equation was used to derive dissociation constants and accessible fractions from steady-state fluorescence data. Our results revealed the ability of compounds to bind to Tau protein. Indeed, these compounds exhibit low binding dissociation constants and low accessible fraction, indicating a significant embedded moiety of compound in Tau protein. In contrast, compounds **1** and **5** showed higher kinetic parameters, suggesting a substantially different mode of interaction with Tau. Our data were consistent with the inhibitory activity of the same compounds against Tau aggregation. Hence, compounds could promote a soluble state of Tau protein. Next, we investigated the effects of compounds on cell viability.

### 2.3. Biological Activity of Compounds with Ring B Substitution

It has been previously reported that flavonoid derivatives, such as QCT, displayed anti-proliferative activity in numerous tumor cells [42,43,44]. We performed MTT assay to determine the impact of QCT and compound **1** on cellular metabolic activity, an indicator of cell viability, proliferation, and toxicity [45]. This approach was carried out on the GBM cells (U87, U251, U138, and T98G), the neuroblastoma cell (SK-N-SH), and the colon adenocarcinoma cell (Caco-2). The compounds QCT and **1** served as reference drugs (Table 2). Surprisingly, we measured no effect of treatment with QCT in all cell models, albeit its IC50 value for inhibition of Tau assembly in vitro. It is possible that membrane permeability disturbed the passage of QCT into used cell lines, as supported by the low predictive value of log P (Table 1). Overall, our data are consistent with previous studies demonstrating that brain tumor cells are poorly sensitive to QCT treatment [46,47]. More interestingly, we measured a reduced metabolic activity of GBM cells for compound **1** with an IC_50_ value ranging between 4.2 ± 0.7 µM and 22.4 ± 5.2 µM among the Tau-expressing GBM cell models, against 33.2 ± 13.3 µM in Caco-2 cells not expressing Tau protein. For compound **1** which showed a low effect on Tau assembly inhibition, we cannot rule out an indirect effect of the 2-aminothiazole moiety, as previously described in [48,49,50].

The effects of the six 2-aminothiazole-fused chromen-4-one derivatives (**2**–**5**, **16**, **17**) were also tested on different cell lines. In the series of compounds **2**–**5**, the most active compound on GBM and neuroblastoma cells was **2**, with an IC_50_ value up to 10-fold lower than **1** in the U87 cell line. Our results indicate that 2-aminothiazole, when fused to flavone heterocycles, brings a better anti-metabolic activity than when detached. However, when the diphenyl ether group on ring C of **2** was replaced by the benzyl group (**3**), ethyl benzoate group (**4**), or carboxyl group (**5**), biological effects were completely lost on all cell lines. Furthermore, compounds **16** and **17**, two structural analogues, did not elicit any activity on all cells. Our results suggest that biological activity could be related to the electronic properties of compounds, particularly considering the hydrophobic substituents on the C ring chromen-4-one, as indicated by the log *p* values (see Table 1 for values). Moreover, the activity of **1** was exclusive to brain tumor cells, suggesting that Tau expression could be a selective condition for the compound’s activity.

### 2.4. Biological Activity of Derivatives with a Fluorinated Substituent on Ring B

We secondly examined the biological effect of ten analogues with a fluorinated ring B (**6**–**15**) by focusing our attention towards GBM-derived cell lines U87, U251, U138, and T98G. Table 2 recapitulates the calculated IC_50_ values.

In the series where R1 is a hydrogen atom on ring C (**6**–**8**), the most active compound was compound **6**, with a fluorine in an ortho position on ring B. When the fluorine was placed in the meta position as in **7**, no effect was observed. Compound **8**, with the fluorine in a para position, exhibited a strong reduction in activity for U251 and U138 cells (respectively, IC_50_ = 3.6 ± 1.1 µM and 10.2 ± 5.0 µM) and null for U87 and T98G cells. For compounds **9**–**12** with hydroxyl substituent on ring C, the biological activities were within the µM range for all GBM cells. More interestingly, compound **12**, lacking fluorine in ring B, still significantly reduced the viability of U87 and T98G cells (respectively, IC_50_ = 4.1 ± 3.4 µM and 1.7 ± 0.9 µM), suggesting that hydrogen and fluorine on ring B confer similar electronic properties to compounds. Moreover, when a methoxide group replaced the hydrogen on ring C as in compounds **13**–**15**, the biological activities were also in the µM range for all cells, with efficacy of the compound **14** up to 6-fold higher than **13** and **15** (respectively, 1.1 ± 0.2 µM in U87 cells, 1.3 ± 0.5 µM in U251 cells, 1.4 ± 0.2 in U138 cells, and 2.7 ± 0.5 µM in T98G cells).

From these observations, the biological effects could be related to the electronic properties of substituents on both C and B rings. In this regard, our results revealed that compounds with a hydroxyl group on C3 of ring C had high activity. Among hydroxylated derivatives, those incorporating fluorine in the ortho and meta positions on the 2-phenyl ring B induced the highest biological effect in both cell lines. For the other compounds, the effect was moderate to negligible. Given the Tau binding properties of these compounds, we investigated whether the anti-metabolic activity of compounds was associated with Tau expression in cells.

### 2.5. The Biological Activity of Compounds ***2*** and ***9*** Depends on Tau Expression

We further measured the anti-metabolic activity of many compounds (**2**–**5**, **9**–**12**, **16**, and **17**) in two U87 cell models expressing (U87 shCTRL) or not (U87 shTau) the Tau protein [20,21] (Table 3). In the series of **2**–**5** and **16** and **17**, compound **2** displayed 3 times higher the activity against U87 shCTRL cells compared to U87 shTau cells (respectively, IC_50_ = 1.4 ± 0.5 µM and 4.7 ± 0.3 µM). This result strongly suggests that the biological effect of **2** could be associated with Tau protein expression.

In the series of compounds **9**–**12**, we measured the anti-metabolic activity of 9 in a µM range for U87 shCTRL cell (IC50 = 1.6 ± 0.5 µM) and no activity in U87 shTau cells. Our data supported that the biological effect of 9 is associated with Tau expression in cells. Further results showed that the re-expression of EGFP-Tau in U87 shTau cells restores compound **9** activity to an IC50 value falling in the same range as that measured when expressing free EGFP in U87 shCTRL cells (Table S1 in Supplementary Materials File S2). It is worth noting that we verified the partial rescue of Tau expression in U87 shTau cells by Western blotting, as previously described in [20]. 

Based on the results from the ring modifications described above, the two analogues **2** and **9** turned out to be the most active ring C and ring B substituents. The two compounds displayed the highest activity, which was associated with Tau expression in GBM cell lines, with IC_50_ values in the µM range. Next, these two compounds were selected for further biological evaluation within the context of understanding their mechanisms of action.

### 2.6. Biological Activity of Compounds Involves No Change of Division and Death of Cells

It is well established that the disruption of microtubules alters diverse biological processes in most cell types such as mitosis and cell death pathways; however, the involvement of Tau in these processes is largely unknown. To understand the role of active compounds **2** and **9** in this context, we examined the effect of these compounds on the cell cycle progression of U87 shCTRL and U87 shTau cells. After 48 h of treatment with compounds (10 µM), flow cytometry was used to quantify the percentage of cells in the G1, S, and G2/M phases. In our experiments, the treatment conditions with the specific antimitotic agent nocodazole (NOC; 1µM) were added as a positive control to our study. Our data are presented in Figure S3 A (in Supplementary Materials File S2).

When U87 shCTRL and shTau cells were treated with NOC, the percentages of cells in phases G1 (respectively, 10 ± 3% and 9 ± 7%) and G2/M (82 ± 6% and 77 ± 4%) were drastically higher than those of untreated cells (%G1 = 56 ± 2% and 69 ± 2%, %G2/M = 28 ± 3% and 18 ± 0.5%, respectively). As expected, the two cell types were blocked in the mitotic phase in the presence of NOC. Moreover, treatment of cells with biological inactive compound **5** had no effect on cycle phases ((%G1 = 53 ± 4% and 62 ± 5%, %G2/M = 34 ± 5% and 25 ± 1%, respectively). Note that this was true even when cells were treated with very high concentrations of **5** (up to 150 µM). More surprisingly, no significant effect on cell cycle progression was measured when the two cells were treated with active compounds **2** and **9**. As a complementary approach, we performed a high-content image analysis of the mitotic index on cells pre-incubated with compounds for 48 h (Figure S3B). As a result, we quantified 1.92 ± 0.79% and 2.5 ± 0.41% of respectively untreated U87 shCTRL and shTau cells in mitosis, indicating a similar proliferative activity for the two cell models. 

As expected, when cells were treated with compound **5**, no significant change of the mitotic index (respectively, 2.47 ± 0.19% and 3 ± 0.39%) was measured compared to untreated cells, whereas a drastic increase of mitotic indexes was measured for NOC-treated cells (9.38 ± 3.55% and 23.0 ± 4.01%). Furthermore, no significant variation of mitotic indexes was observed for cells treated with compounds **2** and **9** compared to untreated cells. Altogether, our data indicated that the biological activity of all tested compounds did not involve cell cycle arrest.

To further investigate the effects of compounds **2**, **5**, and **9** on apoptosis and necrosis, we quantified, respectively, the expression of caspase 3 protein (Casp3), and the activity of the free metabolic enzyme lactate-dehydrogenase (LDH) in the medium (see the Materials and Methods section for details) (Figure S4 in Supplementary Materials File S2). As a result, no change in the expression of Casp3 was measured for the two cell types after 48 h treatment with all compounds (Figure S4A). Our findings showed that the active compounds **2** and **9** induce their biological effect through a caspase-independent process. In addition, the analysis of LDH activity (Figure S4B) in cells treated for 24 h remained similar to untreated cells regardless of the compound used. Once more, the activity of compounds **2** and **9** did not lead to an increased number of cells undergoing necrosis.

Altogether, we showed that active compounds **2** and **9** did not change the distribution of treated cells in G1, S, and G2/M phases. Contrary to usual antimitotic agents such as NOC, our findings revealed that the anti-metabolic activity of compounds did not disrupt cell division. Additionally, these compounds did not trigger the activation of cell death pathways such as apoptosis and necrosis. Subsequently, we examined the impact of compounds on autophagy and mitochondria networks.

### 2.7. Compounds Affect Moderately Autophagy Independently from Tau Expression

**Figure 4:**
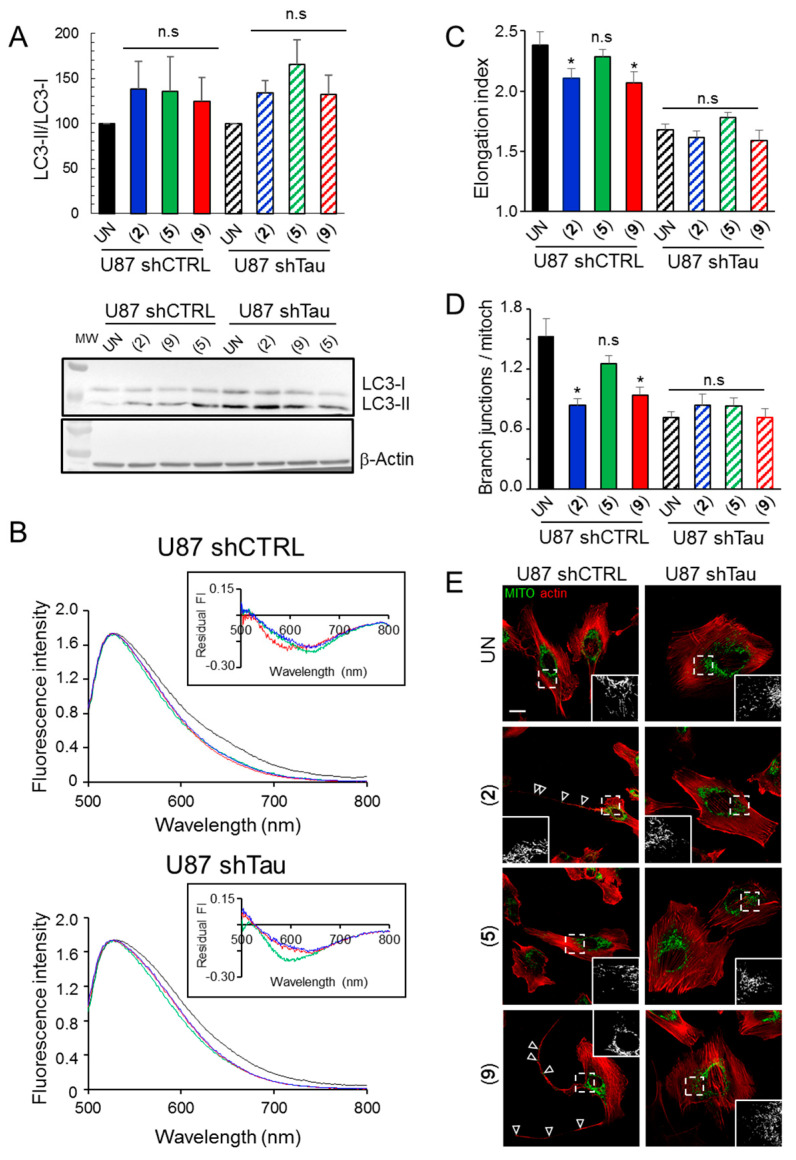
Compounds (**2**) and (**9**) affect autophagy function machinery and alter mitochondrial morphology. The cells are treated with 0.1% DMSO (condition ‘UN’, untreated), or 5 μM compounds (**2**), (**5**), (**9**) for 48 h at 37 °C before analysis. (**A**) Quantities of autophagy LC3 proteins were analyzed by Western blot of U87 shCTRL and U87 shTau cells: for each sample, the ratio of the amount of mature (LC3-II) to immature (LC3-I) isoform was calculated as relative fold of the reference value (with or without the compound); significant statistical differences were calculated using one-way ANOVA with a post hoc Tukey–Kramer HSD test with (*) *p* < 0.01 from three independent experiments; ns: non-significant. The inset in (**A**) shows representative examples of Western blot used for quantification. (**B**) Fluorescence emission of acridine orange for U87 shCTRL (**upper panel**) and U87 shTau (**lower panel**) cells: untreated (black spectra), and treated with compounds (**2**) (blue spectra), (**5**) (green spectra), and (**9**) (red spectra); the spectra are the average data of three independent experiments; Inset: residual fluorescence intensity of acridine orange between treated and untreated conditions: negative fluorescence intensity values show a shift in spectra toward blue wavelengths, indicating a neutral pH of the probe microenvironment. (**C**) Quantification of the mitochondria elongation index in U87 shCTRL (full bars) and U87 shTau (hatched bars) cells: untreated (black bars), and treated with compounds (**2**) (blue bars), (**5**) (green bars), and (**9**) (red bars). (**D**) Quantification of the average branch junctions in mitochondria of U87 shCTRL (full bars) and U87 shTau (hatched bars) cells: untreated (black bars), and treated with compounds (**2**) (blue bars), (**5**) (green bars), and (**9**) (red bars); for (**C**,**D**), significant statistical differences were calculated using one-way ANOVA with a post hoc Tukey–Kramer HSD test with (*) *p* < 0.01 from three independent experiments; ns: non-significant. (**E**) illustrates the distribution of mitochondria in U87 shCTRL and shTau cells untreated); head arrows indicate mitochondria in a few long protrusions; Inset: the dashed box shows a zoomed ROI of the mitochondria network; scale bar: 20 µm.

It is well-described that the intracellular degradative autophagy process occurs in response to cell stress such as organelle damage, the presence of aberrant proteins, and nutritional shortage [51]. To assess whether the active compounds impacted autophagosome formation, we conducted Western blotting to examine the ratio of mature LC3-II to immature LC3-I proteins in U87 shCTRL and shTau cells treated with compounds **2**, **5**, and **9** (Figure 4A). The results indicated no statistically significant changes in ratios for both cell types, indicating that compounds did not affect autophagosomes. To complete these data, a pH-sensitive acridine orange probe was carried out to evaluate late autophagy events such as the fusion of autophagosomes with acidic lysosomal vesicles. Live cells were stained with acridine orange, and then fluorescence emission spectra were recorded (Figure 4B). Following a treatment with compounds, a shift to blue emission was observed for two treated cell types, suggesting a traffic disruption of autophagosomal vesicles. Our findings revealed that compounds **2**, **5**, and **9** interfere with the autophagy processes in a Tau-independent manner.

### 2.8. Compounds ***2*** and ***9*** Impair Mitochondria Network in Tau-Expressing Cells

The organization and dynamics of mitochondrial network are essential for sustaining the bioenergetics functions of this organelle. Numerous natural compounds, including flavonoids, were shown to modulate mitochondria activities [52,53]. Therefore, we further investigated the effect of compounds on mitochondria within the two cell types. Immunostaining of citrate synthase was performed, alongside a labelling of actin networks, and then the two morphologic parameters, elongation index of mitochondria and numbers of branch junctions per mitochondria, were evaluated (Figure 4C,D). Our analysis showed that compounds **2** and **9** induced a moderate decrease (−12.5%) in the elongation indexes of mitochondria in U87 shCTRL cells. In addition, we observed a reduction of 1.8- and 1.6-fold in the number of branch junctions per mitochondria, respectively, for compounds **2** and **9**. No effect of compound **5** was observed. Furthermore, none of the compounds had an impact on the mitochondria networks of U87 shTau cells. Note that we verified by Western blot that the amount of citrate synthase was constant for all experimental conditions (Figure S5 in Supplementary Materials File S2). Figure 4E comprises a set of representative images illustrating the mitochondria network reorganization after treatment with 5 µM of compounds for 24 h in the two cell types. Our observations indicated that mitochondria were both shorter and less branched when U87 shCTRL cells were treated with compounds **2** and **9**. Furthermore, many mitochondria were localized within a few elongated protrusions labelled for actin networks (head arrow on images), suggesting that some fractionated mitochondria were translocated. On the other hand, no effect of three compounds was observed in U87 shTau cells. 

Our data showed that compounds **2** and **9** impaired the fusion of mitochondria in U87 shCTRL cells. In addition, we observed many actin-rich protrusions, indicating that these compounds could induce a strong remodeling of cytoskeletons. Next, we focused on the impact of compounds on microtubule networks. 

### 2.9. Compounds ***2*** and ***9*** Induce Migratory Defect Due to Remodeling of Microtubule Network in Tau-Expressing Cells

**Figure 5:**
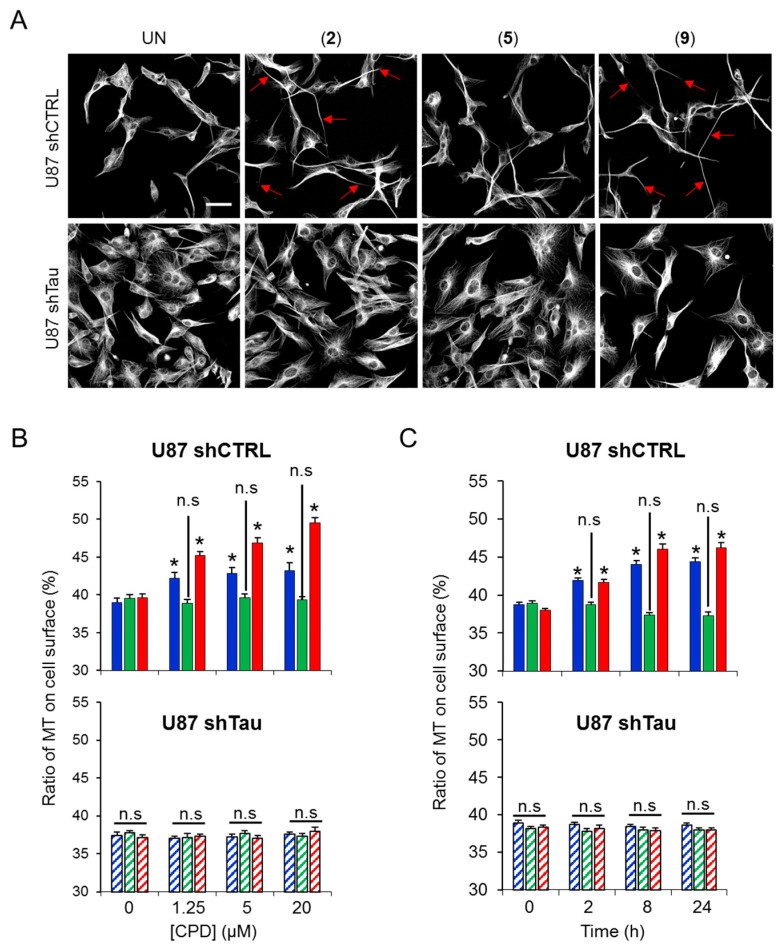
Compounds (**2**) and (**9**) induce a dose- and time-dependent remodeling of cytoplasmic microtubule networks of glioblastoma U87 cells. The two U87 shCTRL (full bars) and shTau (hatched bars) cells were incubated with compound (**2**) (blue bars), (**5**) (green bars), or (**9**) (red bars) at 37 °C, fixed, and then labelled by indirect immunofluorescence of alpha-tubulin. The microtubule networks are visualized by CLSM. (**A**) is representative of the results obtained with 0.1% DMSO (condition ‘UN’, untreated), or with 5 µM of compounds (**2**–**9**) for 24 h; red arrows indicate few long protrusions with a labelled microtubule; scale bar: 20 µm. (**B**) Effect of concentration of compounds (0–20 μM); (**C**) Effect of the incubation time with compounds on cytoplasmic networks; for (**B**,**C**), ratios of microtubules on total cell surfaces (in%) are calculated for cells treated with compounds (**2**–**9**), all reported values correspond to means ± s.e.m. of 50 cells; significant statistical differences are calculated using the Tukey–Kramer HSD test with * *p* < 0.05 between the value obtained for conditions ‘0 µM’ or ‘0 h’ and the others for each graph; n.s., not significant.

We previously demonstrated that Tau governs the necessary remodeling of microtubules for effective migration of GBM U87 cells [20]. Therefore, the impact of compounds **2** and **9** on the distribution of the microtubule network was investigated. As shown in Figure 5A, the set of representative images illustrates the high microtubule network reorganization after treatment with 5 µM of drugs for 24 h. In U87 shCTRL cells treated with compounds **2** and **9**, we observed that the microtubule networks were quite dense in the cell body, and some elongated protrusions contained microtubule bundles (red arrows in figures). In contrast, treatment with compound **5** induced no change in the microtubule networks. Moreover, no significant effect of all compounds was observed for treated U87 shTau cells. As Supplementary Materials, we verified with turbidity time courses of in vitro Tau-promoted microtubule assembly in the presence of compounds that the rate of assembly as well as the final amounts of microtubules were similar to the control experiment (without compound) (Figure S6). Our data suggest an exclusive effect of the compounds to Tau in cells. Two experimental conditions were designed to explore (1) the effect of drug concentrations and (2) the effect of drug incubation time on the microtubule cytoskeleton. For this purpose, we performed an indirect immunofluorescence staining of the microtubule network in untreated or drug-treated U87 shCTRL and shTau cells. From images collected by CLSM, the state of microtubule assembly was calculated as being the ratio of the surface of the microtubule network to the total surface of cells.

**Figure 6:**
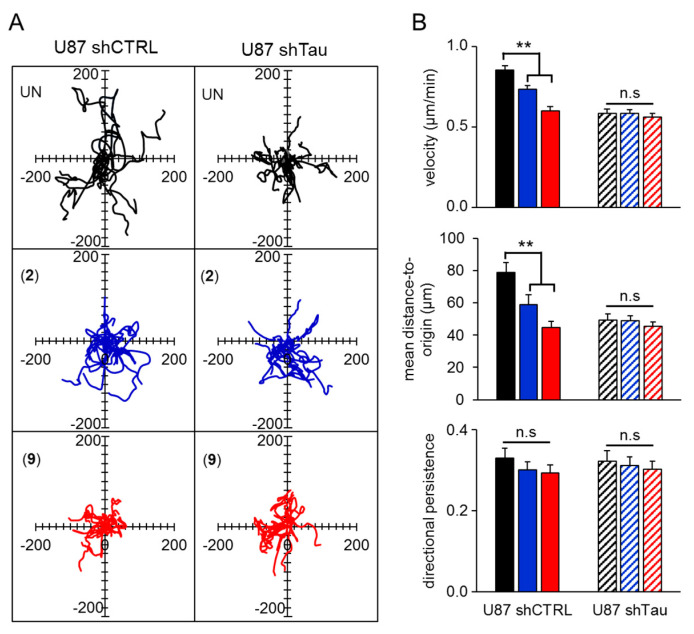
Compounds (**2**) and (**9**) disrupt migration of glioblastoma U87 cells. Cells were seeded at 10^3^ cells/well on fibronectin pre-coated 24-well plates and then treated with DMSO (black bars), 1.4 µM of compound (**2**) (blue bars), or 1.6 µM of compound (**9**) (red bars) immediately preceding live image acquisition for 5 h with an image recorded every 10 min. (**A**) Representative migration paths of ten cells are reported by using position parameters: black line for untreated (‘UN’) cells (**upper panels**), blue line for cells treated with (**2**) (**middle panels**), red line for cells treated with (**9**) (**lower panels**); (**B**) Migratory parameters were determined for cells as in A: velocity (**upper panel**), mean distance to origin (**middle**) and directional persistence (**lower**) were calculated for shCTRL cells expressing Tau (full bars) and shTau cells depleted for Tau (hatched bars). Data are expressed as the mean ± s.e.m. of n = 80 cells and are representative of three independent experiments. ** *p* < 0.01; n.s., not significant (one-way ANOVA with a post hoc Tukey–Kramer HSD test).

To investigate the effect of drug concentration, the microtubule network was visualized after a 24 h treatment with 0–20 µM of compounds **2**, **5**, and **9** (Figure 5B,C). In untreated cells, it was determined that 39 ± 5% of the cell surface was occupied by the fluorescent microtubule network in both U87 shCTRL and shTau cells (Figure 6A). All these values come close to the range of 31–41% of tubulin in microtubules in cultured tissue cells, as previously determined by Ostlund et al. [54] and our team [38]. For compounds **2** and **9**, a higher quantity of labelled microtubules was evident after treatment with a concentration of 1.25 µM (respectively, 42.2 ± 6.7% and 45.2 ± 4.6%) in U87 shCTRL and was more apparent at 20 µM (respectively, 43.2 ± 9.3% and 49.5 ± 6.3%). When cells were treated with compound **5**, no effect was observed at equal concentrations. For drug-treated U87 shTau cells, we observed no change for all compounds whatever their concentrations. All these data highlight the higher biological effect of compounds **2** and **9** vs. **5** in cells expressing Tau protein. 

The effect of incubation time with compounds on the microtubule network was then examined. For this, cells were incubated with 5 µM of compounds for 0–24 h at 37 °C (Figure 6C). With compounds **2** and **9**, the higher quantity of labelled microtubules was effective at 2 h of incubation and maximal after 8 h for U87 shCTRL cells (respectively, 44.1 ± 3.8% and 46.0 ± 6.1%). On the other hand, compound **5** had no effect on microtubule networks of U87 shCTRL cells. Moreover, we measured no change of microtubule distribution for all compounds for drug-treated U87 shTau cells. Our results indicate that compounds **2** and **9** are more active on cytoskeleton than compound **5** in U87 shCTRL cells.

Furthermore, we assessed the influence of compounds **2** and **9** on cell migration. For this purpose, we performed a two-dimensional (2D) random motility assay on both cell types using time-lapse video microscopy, as we previously described in [20]. The result of a representative migration experiment (where the migration origin of each cell was defined at x = 0, y = 0; n = 5 cells) is shown in Figure 6A. As results, the motility of U87 shTau cells was lower than that of U87 shCTRL cells (right panels). In addition, we analyzed the trajectory obtained from the time-lapse recording of each 80 individual cells in order to calculate migratory parameters such as velocity, distance to origin, and directional persistence (right panels) during migration (Figure 6B). The U87 shTau cells exhibited a significant reduction in both velocity (−32%) and distance to origin (−38%) compared to U87 shCTRL cells (bars with solid colors). Moreover, we observed no change in directional persistence across cell types. These results remain consistent with our previous data [20]. On the other hand, when U87 shCTRL cells were treated with compounds **2** and **9**, we respectively measured a 15 and 30% decrease in velocity, as well as a 25 and 43% decrease in distance to origin (middle panel). Interestingly, these migratory parameters fell within the same range as those observed for U87 shTau exposed to compounds. Note also that treated U87 shTau cells displayed similar values of velocity, distance to origin, and directional persistence as for untreated cells. Therefore, following compounds **2** and **9** treatment, our data remain consistent with expected results for drugs that induce microtubule stabilization mediated by Tau.

In conclusion, our data showed that compounds **2** and **9** induced microtubule remodeling in bundles in U87 shCTRL cells, concomitantly with impairment of cell migration. In addition, our turbidimetry data indicate that the interaction of these compounds with Tau does not change the microtubule-stabilizing function of the protein. Taken together, the interaction of these compounds with Tau in cells could induce accumulation of the protein along the length of microtubules, leading to a strong stabilization of the cytoskeleton.

## 3. Discussion

Tau, and its active role in the assembly of tubulin, were identified in the 1970s [12,13]. Indeed, it is well documented that Tau promotes assembly and stabilization of microtubules both in vitro and in vivo [14,15,55]. In this way, we investigated the impact on Tau of a new class of hybrid 2-aminothiazole-fused flavonoid derivatives. Using an in vitro ThS fluorescence assay, we observed that many compounds such as **2** and **9** were able both to inhibit heparin-induced Tau assembly and to dissolve mature Tau filaments. Moreover, we demonstrated that these derivatives exhibit both low-binding dissociation constants and low accessible fraction, indicating a significant embedded moiety of these compounds in the Tau protein. Taken together, we could identify at least one potential binding site of compounds with Tau, located at Tyr310 in the repeated domain 3 (R3) region. It is plausible to think that compounds may interfere with β-sheet secondary structure of the microtubule-binding domains and/or the stacking of Tau molecules in PHFs. Notably, assembly studies revealed that β-sheet formation in the first six residues of R2 (^275^VQIINK^280^), and/or the first six residues of R3 (^306^VQIVYK^311^), is important for Tau protein filament formation [56,57,58]. Furthermore, recent cryo-EM studies by Zhang et al. have revealed various structures of heparin-induced Tau filaments, with the predominant “snake” conformation representing around 45% of the total four conformations of heparin-induced Tau filaments [59]. Taking this cryo-EM structure (PDB code 6QJH), we performed in silico molecular docking of compound **9** with Tau (Figure S7 in Supplementary Materials File S2). Up to 150 poses were predicted, and the one with the least energy at binding equilibrium displayed the planar structure of TZF heterocycle close to tyrosine residue in the VQIVYK sequence of R3 (score: −6.73 kcal/mol; cluster size: 2; illustrated in Figure S7 in Supplementary Materials File S2). Furthermore, we observed that the fluorinated ring B was likely embedded between two successive rungs in the Tau filament. This could result in potent steric hindrance for Tau assembly. Overall, it has been reported that many VQIVYK inhibitors can block the heparin-induced assembly of full-length Tau [60,61,62]. Recently, the Das’ team reported by AFM analysis that 10 µM QCT abrogated R3 peptide fibrillation [63]. More interestingly, the authors demonstrated that QCT was able to prevent seeding of endogenous Tau aggregates by exogenous seeds. Our model of interaction between TZF derivatives with Tau protein is consistent with these findings.

Further in-cell studies were conducted in order to establish a relationship between the rings B and C modifications and the biological activity of compounds **2**–**17** across various cell lines. Among the molecules tested, analogues **2** and **9** drew our attention for their µM anti-metabolic activity correlating with the level of Tau expression in the cell. Further evidence for this relationship was derived from the recovery of the activity of compound **9** after the re-expression of the protein in our Tau-depleted cell clone U87 shTau. After ruling out the possibilities of an impact of compounds on both cell cycle and apoptosis/necrosis pathways, we further explored the compound-mediated disruption of autophagy, a cell death mechanism that remains enigmatic regarding tumor cells [64,65]. In our study, a qualitative assay with a fluorescent pH-sensitive acridine orange probe was performed to evaluate the formation of autolysosomal vesicles. Autolysosomes are a late step in the autophagy pathway corresponding to the fusion of autophagosomes with acidic lysosome vesicles [64]. Here, all tested compounds induced a shift to blue of acridine orange fluorescence emission in two cell types, indicating a neutral pH of the probe microenvironment. Altogether, our observations suggest that TZFs disturb the autophagy process, independently from Tau expression.

Considering the antioxidant properties of the flavonoid heterocycle, we examined the impact of compounds on mitochondria. Analysis of mitochondrial morphology showed that treated U87 shCTRL cells have significantly fewer branches per mitochondrial network, as well as a relevant increase in mitochondria fission (Figure 4). No change was observed for U87 shTau cells. Our analysis indicates that these compounds improve abnormal Tau-induced mitochondrial dysfunction. Mitochondrial activity is essential for organelle biogenesis and thereby for the sufficient supply of cytosolic ATP-particularly important for tumor cells with high energy demands like GBM cells. Defective mitochondria can be removed by mitochondrial autophagy, a process known as mitophagy, which can be initiated when the mitochondrial membrane potential dissipates due to functional impairment. This depolarization activates the Parkin/PINK1 pathway, leading to mitochondrion removal [66,67]. In this way, Cummins et al. have identified in N2a neuroblastoma cells the underlying pathomechanism, wherein Tau accumulation aberrantly interacts with Parkin in the cytosol, preventing it from proper translocation to defective mitochondria [68]. It is tempting to think that the binding of our compounds to Tau improves the inhibitory effect of the protein on mitophagy. Moreover, it has been recently reported that the ubiquitous Tau-related microtubule-associated protein 4 (MAP4) can bias the bidirectional transport of mitochondria toward the microtubule minus-ends [69]. Interestingly, these authors also demonstrated that MAP4 achieves this bias by tethering the cargo to the microtubules, allowing it to impair the force generation of the plus-end motor kinesin-1. In our study, our observations revealed that compounds **2** and **9** induce an abnormal distribution of mitochondria in protrusions of U87 shCTRL cells, suggesting that Tau-decorated microtubules could impair kinesin-based transport of damaged mitochondria.

Besides, we demonstrated that treatment with compounds **2** and **9** mainly affected velocities and distances to origin in U87 shCTRL cells, two migratory parameters that we previously reported to be enhanced by Tau expression [20,21]. Indeed, we proved that Tau protein governs the remodeling of microtubule and actin networks for the retraction of the tail of U87 cells, which is necessary for effective migration [20]. Hence, we examined the status of the microtubule network in cells treated with these compounds. Our main observations revealed microtubule remodeling into bundles at newly formed protrusions in U87 shCTRL cells. Moreover, we showed that this effect was dependent on both concentration and incubation time. Many studies reported that an accumulation of Tau in the cell induces the formation of neurite-like protrusions [70,71,72,73]. Altogether, our data are consistent with increased Tau-mediated stabilization of microtubules.

In conclusion, seventeen 2-aminothiazole-flavonoid hybrid derivatives were synthesized, and structure activity relationships were derived from our findings. Among these, two compounds stood out: 2-amino-7-(3-phenoxyphenyl)-9H-chromeno [6,5-d]thiazol-9-one (compound **2**) and 2-amino-7-(2-fluorophenyl)-9H-chromeno [6,5-d]thiazol-9-one (compound **9**). Both of each show a high inhibitory activity of Tau fibrillation, coupled with a biological effect that was consistent with Tau expression in GBM cells. Notably, these compounds could lead to an accumulation of Tau in cells which was concomitant with both a reduced motility of cells and mitochondria impairment. All data correlate well with Tau accumulation governing a remodeling of the microtubule network. Our results provide a strong experimental basis to develop new potent molecules for Tau-related therapies such as cancer and neurodegenerative diseases. Further work with these compounds may include testing them for other biological activities, given that the TZF skeleton is a privileged structure.

## 4. Materials and Methods

### 4.1. Chemistry

**Scheme 1:**
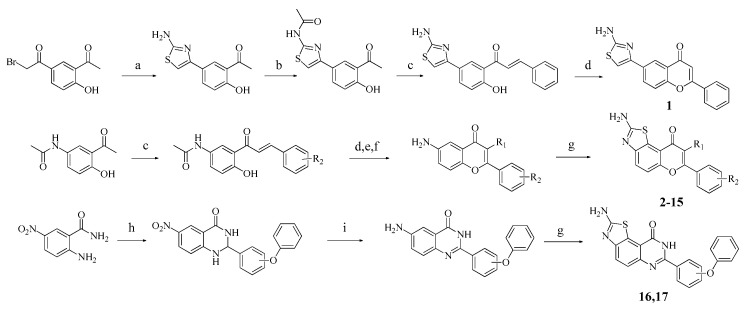
Synthesis of thiazoloflavonoid derivatives. Reagents and conditions: (**a**) thiourea, EtOH, rt overnight. (**b**) (AcO)_2_, reflux, 1 h. (**c**) aldehyde, MeOH, LiOH, MWI (140 °C, 300 W, 20 min). (**d**) R1 = H, DMSO/I_2_ MWI (120 °C, 300 W, 20 min). (**e**) R1 = OH, NaOH/H_2_O_2_, r.t., 24 h. (**f**) R1 = OMe, DMS, Acetone/K_2_CO_3_, 60 °C, 6 h. (**g**) KSCN, AcOH, Br_2_, r.t., 2 h. (**h**) aldehyde, DMF/ZnCl_2_, r.t. 1 h. (**i**) H2, Pd/C, EtOH.

Unless otherwise specified, all chemicals were purchased from commercial suppliers and directly used as received without additional purification. Column chromatography was carried out with silica gel (200–300 mesh) to purify products, using proper solvents as the eluent system. NMR spectra were recorded at 300 MHz for 1H NMR spectra and 75 MHz for 13C NMR spectra by spectrometer (Advance 300 spectrometer, Bruker, Wissembourg, France). Chemical shifts are quoted in parts per million referenced to the appropriate solvent peak (^1^H NMR: CDCl_3_ 7.26 ppm, DMSO-d_6_ 2.49 ppm, ^13^C NMR: CDCl_3_ 77.0 ppm, DMSO-d_6_ 39.5 ppm). The following abbreviations were used to describe peak splitting patterns when appropriate: s = singlet, d = doublet, t = triplet, q = quartet, m = multiplet. Mass spectra were performed on a spectrometer operating on ESI-TOF. The synthetic process and full characterization data of compounds **1**–**17** are depicted in Scheme 1 and Supplementary Materials File S1, respectively.

### 4.2. Biology

#### 4.2.1. Cell Culture and Transfection Protocol

All cell lines were purchased from ATCC, Gaithersburg, MD, USA. Cells from human glioblastoma (clones U87 wt, HTB-14; U118, HTB-15; U251, HTB-12; U138, HTB-16), neuroblastoma (clone SK-N-SH, HTB-11), and colorectal adenocarcinoma (clone Caco-2, HTB-37) were routinely grown at 37 °C in a humidified atmosphere containing 5% CO_2_. Cells were maintained by regular passages in complete media supplemented with 10% FBS, 2mM of L-glutamine, 100 U/mL of penicillin, and 50 U/mL of streptomycin (Invitrogen, Paris, France). For U87 wt, U251, U138, and SK-N-SH cells, the medium consisted of EMEM (Lonza, Levallois Perret, France). U118 and Caco-2 lines were maintained in DMEM. The U87 wt-derived lines, U87 shCTRL, and U87 shTau2 cells were continuously maintained in complete EMEM medium supplemented with 0.4 µg/mL of puromycin. The shRNA sequences and the protocol of clone selection and Tau downregulation were previously described in [20]. For Tau expression rescue, the U87 shCTRL and U87 shTau cells were seeded two days prior to experiments at 4.10^5^ cells per 60 mm dish in 6 mL of complete medium. Cells were transfected using LipofectAmine 2000 according to Invitrogen instructions and 8 µg EGFP-free plasmid constructs (for U87 shCTRL) and EGFP-hTau40 plasmid constructs (for U87 shTau) [74] were used in total. One day prior to experiments, all cells were plated in a 96-well microtiter plate to prepare for viability assays. Cells were free from mycoplasma as determined by mycoalert tests (Lonza). 

#### 4.2.2. Cell Viability Assay

Cells harvested from sub-confluent monolayers were seeded at 15,000 cells per mL in a 96-well microtiter plate (Cambridge Technology, Labège, France) and cultured for 24 h under standard conditions. Standard medium was then replaced by fresh medium containing no drugs or compounds at different concentrations. The surviving cells were quantified after 72 h by the MTT assay, according to the manufacturer’s instructions. Briefly, 20 µL of 3-(4,5-diMethylThiazol-2-yl)-2,5diphenyl Tetrazolium bromide (MTT) solution at 5 mg.mL^−1^ was added to cells for 3 h at 37 °C. The supernatant was discarded and replaced by 100 µL of dimethyl sulfoxide to dissolve formazan crystals. The absorbance was then read at 600 nm by spectrophotometry. For all concentrations of a given compound, cell viability was expressed as the percentage of the ratio between the mean absorbance of treated cells and the mean absorbance of untreated cells. Three independent experiments were performed, and the data were expressed as percentage of survival (using the untreated cells as 100%) and subjected to statistical analysis (n = 3). The IC_50_ was calculated using the Chou and Talalay method [75]. 

#### 4.2.3. Tau and Tubulin Purification

The full-length human Tau (hTau40) was expressed in a transformed *E. coli* BL21 (DE3) strain and purified as described previously [76]. Lyophilized Tau was stored at −80 °C and directly resuspended in the appropriate buffer before use. Tau concentration was measured at 280 nm using an extinction coefficient of 7700 M^−1^ cm^−1^.

Tubulin was extracted and purified from lamb brains by Weisenberg procedure consisting of ammonium sulfate fractionation and ion exchange chromatography as previously described [76]. Tubulin was stored in a sucrose buffer in liquid nitrogen. Before use, tubulin is equilibrated by passing it through cold sephadex G-25 columns pre-equilibrated with 20 mM of NaPi pH 6.5, 1 mM of TCEP, and 10^−4^ M of GTP. Tubulin concentration was determined spectrophotometrically at 275 nm using a molar extinction coefficient equal to 109,000 M^−1^ cm^−1^ in 6 M of guanidine hydrochloride. 

#### 4.2.4. Turbidimetry for In Vitro Tubulin Polymerization

The polymerization of tubulin was monitored by measuring the apparent absorbance at 350 nm using a Beckman DU 7400 spectrophotometer at 37 °C. Tubulin at a final concentration of 10 µM was mixed with compounds at the indicated concentration and immediately placed into the spectrometer chamber. When the signal was stable, Tau at a final concentration of 5 µM was added to induce microtubule formation. Measurements were continued until turbidity values reached a plateau.

#### 4.2.5. Tau Oligomerization Inhibition Assay

Tau oligomerization inhibition assay was performed at 37 °C in 96-well plates by monitoring thioflavin-S fluorescence at 520 nm after excitation at 440 nm using a microplate reader (POLARstar Omega system, BMG Labtech, Paris, France). The Tau oligomerization process was induced by heparin. A 2 µM quantity of Tau was mixed with 0.75 µM of heparin, 5 µM of thioflavin S, and compounds at the indicated concentration in buffer A (25 mM of NaPi, 25 mM of NaCl, 0.5 mM of TCEP, 5% DMSO, pH = 6.9 adjusted with NaOH) and immediately placed into the plate reader. Measurements were taken each 15 min for 8.75 h or until a plateau was reached. The plates were shaken at 200 rpm for 10 s before each measurement. The real value of fluorescence at the plateau was determined using SciDAVis v1.23 software. Data were fitted to the SciDAVis built-in ExpGrowth equation by means of nonlinear least-squares regression analysis.

Y = Y_0_ + A × exp(x/t) (1)

The unscaled Levenberg–Marquardt algorithm was used, and initial values were given to Y_0_, A, and t. The final Y_0_ value of the model corresponds to the plateau value. The plateau values were then used to calculate the Tau oligomerization inhibition percentage. The IC_50_ for each compound was calculated from the scatter plot of Tau oligomerization inhibition (in percentage) as a function of log10 of compound concentration. The IC_50_ value (in µM) thus corresponds to ten raised to the power of the slope of the trendline of the plot.

#### 4.2.6. Fluorescence Quenching and Binding Parameters

Fluorescence measurements were performed with a Horiba Jobin Yvon FluoroMax-3 spectrofluorometer. Tau (5 µM) was titrated with compounds (0–100 µM) in buffer B (20 mM of NaPi, 1 mM of TCEP, 5% DMSO and pH = 6.8). For experiments, the excitation wavelength was fixed at 275 nm, and the emission spectra were recorded between 280 nm and 500 nm at room temperature. The inner filter effects were corrected according to Lakowicz [77] as follows:

F = F_obs_ × exp{(A_em_ + A_ex_)/2} (2)

where F_obs_ and F are the observed and corrected fluorescence values at the emission wavelength of 303 nm. A_ex_ and A_em_ are the absorptions at the excitation and emission wavelengths, respectively, calculated with A = ε.l.C, in which ε is the extinction coefficient of Tau, l is the path length of cuvette, and C is Tau concentration.

We measured fluorescence quenching when Tau was complexed to compounds. Then, we applied the Stern–Volmer equation to determine the dependence between variations of fluorescence intensity of Tau and the concentration of quenchers (all tested compounds), as follows:

F_0_/F = 1 + K.[Q] (3)

where F_0_ is the initial fluorescence intensity of Tau recorded at 303 nm without compound, Q the concentration of compound (in M), K the associative constant (in M^−1^).

In our condition, we observed no linearity of plots between the ratios F_0_/F and [Q]. We supposed that quenched Tau fluorophores were constituted of two accessible (F_0a_) and buried (F_0b_) populations. The total fluorescence in the absence of quencher (F_0_) is given by:

F_0_ = F_0a_ + F_0b_ (4)

where 0 refers to the fluorescence intensity in the absence of quencher.

In the presence of quencher, we defined fa as the fraction of the initial fluorescence of Tau accessible to compounds:

f_a_ = F_0a_/(F_0a_ + F_0b_) (5)

Therefore, the intensity of the accessible fraction (f_a_) is decreased according to the Stern–Volmer equation, whereas the buried fraction is not quenched, as given by:

F = {F_0a_/(1 + K.[Q])} + F_0b_ (6)

Then, subtraction of Equations (6) and (4) yields:

ΔF = F_0_ − F = F_0a_ × {K.[Q]/(1 + K.[Q])} (7)

At last, inversion of Equation (7) followed by division into Equation (4) yields:

F_0_/ΔF = {1/(f_a_.K.[Q])} + 1/f_a_ (8)

We applied this modified form of the Stern–Volmer equation to graphically determine the values of f_a_ and K. For our experiments, we plotted F_0_/ΔF against 1/[Q], then we determined the fa value at the point of intersection with the *y*-axis, while the slope represented (f_a_K)^−1^.

Then, the dissociation constants K_D_ (in M) from Tau were calculated for all compounds, as follows:

K_D_ = 1/K (9)

#### 4.2.7. Cell Cycle and Mitosis Index

Measurements of cell cycle from DNA content were performed by flow cytometry as described before [38]. Briefly, 4.10^5^ cells in exponential growth were seeded in 6-well plates. After 24 h, they were treated with 10 µM of compounds for 48 h. Nocodazole was used as a positive control with a concentration of 1 µM. The trypsinized cells were harvested and then centrifuged (1200 rpm, 5 min, at room temperature). The cell pellet was resuspended in 1 mL of PBS 1× at 4 °C, and then an extra 4 mL of cold PBS was added to wash the pellet. Cells were then centrifuged again (1200 rpm, 5 min, at 4 °C). To fixate the cells, the pellet was resuspended in 100 µL of cold 70% ethanol, then an additional 900 µL was added and cells were incubated for 30 min at −20 °C. After that, 9 mL of cold PBS was added, and cells were centrifuged (2000 rpm, 5 min, at 4 °C) to remove ethanol. The cell pellet was then resuspended in the staining mix containing 50 µg.mL^−1^ of propidium iodide (Molecular Probes, Paris, France) and 100 µg.mL^−1^ of RNAse A (Sigma-Aldrich, Saint-Quentin-Fallavier, France) in PBS for 20 min at room temperature in darkness. Samples were analyzed on a Beckman–Coulter–Gallios flow cytometer using the Kaluza for Gallios Acquisition v1.0 software. Propidium iodide was excited at 488 nm, and fluorescence was analyzed at 620 nm on channel FL-3. For the analysis, there is considerable overlap between early S phase and G1 and between late S phase and G2/M due to broadening of the distribution caused by variability in the staining of the cells and instrumental variability. Therefore, the percentages of cells in the sub-G1, G1, S, and G2/M phases of the cell cycle were calculated using the Watson et al. algorithm for cell cycle analysis in Kaluza for Gallios Analysis v2.1 software [78]. The software automatically adjusted gates based on PI median fluorescence intensity of the cells and their frequency among total cells. The number of cells in each phase was divided by the total number of analyzed cells, and the values were reported as percentage of the total cells for each cell cycle phase.

For scoring mitotic indexes, an automated high-content screening confocal microscopy approach was employed to identify cells undergoing mitosis from a large population of cells. For this, cells nuclei were stained with DAPI. Briefly, fibronectin-coated clear-glass-bottom black 96-well imaging plates were seeded with 100 µL of cell suspension (60,000 cell.mL^−1^) in each well, with both U87 shCTRL and U87 ShTau cell lines. Cells were treated with 5-times IC_50_ of compounds for 48 h, then they were fixated inside the palates with 4% formaldehyde (in PBS pH 7.4) for 20 min at room temperature, permeabilized with PBS-Triton X-100 0.5% for 10 min at room temperature. DNA was then stained with DAPI (10 µg.mL^−1^) for 5 min at room temperature and in the darkness. After being washed, cells were kept in PBS at 4 °C and imaged while in PBS. Automated confocal microscopy was carried out using an Opera Phenix (PerkinElmer) high-content screening microscope using a 20× Air/0.4 NA air objective. For each condition, 6 wells were prepared and a total of 61 fields were acquired for each well. Image analysis was performed using Harmony high-content analysis v4.8 software and PhenoLOGIC Machine Learning technology, which uses a learn-by-example approach. As a first step, nuclei were segmented and multiple parameters for these nuclei were calculated using Harmony v4.8 software building blocks. After training, PhenoLOGIC Machine Learning then selects the best parameters to detect nuclei undergoing the M phase of mitosis. From this, the mitotic index of each condition was calculated by dividing the number of cells undergoing mitosis over the total number of cells.

#### 4.2.8. Measurement of Autophagy by Spectrofluorometry

Briefly, 2.10^5^ cells in exponential growth were seeded in 6-well plates. After 24 h, they were treated with 5 µM of compounds for 48 h. Trypsinized cells were harvested, counted, and then centrifuged (1200 rpm, 5 min, at RT). The cells pellet was resuspended in 2 mL of acridine orange diluted in complete culture media to a final concentration of 1 µg/mL and incubated for 15 min in the dark. Cells were then collected by centrifugation (1200 rpm, 5 min, at RT). The cells pellet was resuspended in PBS to a suspension concentration of 2.10^5^ cells/mL. The suspension was transferred to a quartz cuvette, and then fluorescence emission of acridine orange was measured with a FluoroMax-3 spectrofluorometer (Horiba Jobin Yvon, Palaisseau, France) (excitation at 490 nm).

#### 4.2.9. Immunofluorescence of Mitochondria and Microtubules

For indirect immunofluorescence of mitochondria, 10^4^ cells were plated on cover glass and incubated for 24 h with compounds. Cells were then fixed in 4% formaldehyde (in PBS pH 7.4) for 20 min at RT, permeabilized with PBS-Triton X-100 0.5% for 10 min at RT. Immunostaining was carried out overnight at 4 °C with a primary anti-citrate synthase antibody (dilution 1:400 in PBS-BSA 3%; SC390693; SantaCruz Biotechnologies, Heidelberg, Germany). The following day, the cells were washed then incubated with anti-mouse secondary antibody (dilution 1:800 in PBS-BSA 3%; A-11029; Invitrogen) and TRITC-phalloidin (dilution 1:2500 in PBS-BSA 3%; P1951; Sigma-Aldrich, Saint-Quentin-Fallavier, France) for 1 h at RT in the dark. For the microtubule network, the same protocol for mitochondria labeling was carried out by replacing with primary anti-α-tubulin antibody (dilution 1:400 in PBS-BSA 1% from a 1 mg/mL solution; monoclonal antibody, clone DM1A, Sigma-Aldrich, Saint-Quentin-Fallavier, France). Next, cells were washed in PBS and cover glasses were mounted with a drop of ProLong^®^ anti-fade solution (Invitrogen). Mitochondria and cytoskeletons were imaged using a confocal laser scanning microscope (CLSM) Leica SP5 with a Leica inverted microscope, equipped with a Plan-Apochromat 63×oil immersion objective (NA = 1.4). Each image was recorded with the CLSM’s spectral mode selecting specific domains of the emission spectrum. The Alexafluor488 and FITC fluorophores were excited at 488 nm with an argon laser, and fluorescence emission was collected between 496 nm and 535 nm. The TRITC fluorophore was excited at 543 nm with a helium–neon laser, and fluorescence was collected between 560 and 620 nm. The public-domain Fiji v1.54f software was used for image analysis.

#### 4.2.10. Analysis of Mitochondria and Microtubule Networks in Cells

The analyses of mitochondria and microtubule networks were performed using, respectively, the ‘Mitochondria analyzer’ plugin for Fiji v1.54f software and the method described in our earlier work [38]. Briefly, a series of images were transferred to the software and corrected for background fluorescence intensity contribution. Next, the surface of 50 single cells was measured by considering the cell shape as the boundary of labelling of actin or α-tubulin cytoskeletons (respectively for measurement on mitochondria or microtubules). Then, the mitochondria elongation and mean branch junction per mitochondria parameters, and the surface of the microtubule network, were measured by performing systematically the Otsu method to reduce all gray-level images to binary images. Note that the Otsu plugin in ImageJ is an algorithm which searches for the threshold minimizing the intra-class variance of pixel intensities, defined as a weighted sum of variances of the two classes; the local set is a circular region of interest, and the central pixel is tested against the Otsu threshold found for that region [79,80]. Moreover, the ratios of microtubules on cell surfaces were calculated as percentages.

#### 4.2.11. Western Blotting

Cells were lysed with freshly made RIPA buffer derivative (4.3 mL of TBS 1×, 50 µL of SDS 10%, 10 µL of EDTA 0.5 M, 500 µL of Triton 10×, and 1% inhibitors of protease and phosphatase). After 30 min of lysis at 4 °C under agitation, lysates were centrifuged at 10,000× *g* for 10 min at 4 °C. Equivalent amounts of proteins from the supernatant fraction were subjected to Western blot analysis. Protein samples were loaded (40 μg/lane) and separated on 10 or 12% sodium dodecyl sulfate-polyacrylamide gels. The separated proteins were transferred onto nitrocellulose blotting membrane using the Bio-Rad transfer system. Before blocking, the blots were stained with Ponceau red to visualize transfer efficiency. The membranes were incubated with blocking solution (5% milk and 0.05% Tween 20 in TBS1×) for 1 h and then incubated overnight with the proper primary antibodies. The membranes were then washed 3 times with a TBST (TBS plus 0.05% Tween 20) solution and incubated with peroxidase-conjugated secondary antibodies for 1 h. The membranes were again washed 3 times with TBST and one time with PBS and revealed using the Immobilon^®^ Western Chemiluminescent HRP substrate and visualized using a G-Box imaging system (Syngene). The band intensity was quantified using the ImageJ v1.50g software. The following primary antibodies were used: anti-citrate synthase (SC390693; SantaCruz Biotechnologies), anti-LC3 (3868T, cell signaling), anti Casp3 (9662, cell signaling), anti-β-actin (sc-47778, SantaCruz Biotechnologies), anti-GAPDH (G8795; Sigma Aldrich). The HRP-coupled secondary antibodies were purchased from Cell Signaling Technology. The band intensity was quantified using the NIH ImageJ v1.50g software.

#### 4.2.12. In Vitro 2D Cell Motility Assays and Analysis

Cell motility was recorded by time-lapse videomicroscopy, as previously described [20]. Briefly, cells were trypsinized and then seeded on Fibronectin pre-coated 24-well plates at 10^4^ cells per well and allowed to adhere at 37 °C for 2 h in a 5% CO_2_ incubator. The cells were briefly rinsed in imaging medium (EMEM containing 10% FBS) to remove unattached cells before imaging. For conditions using compounds, agents were added to cells 15 min before launching the recording of the time-lapse series. Next, the plates were placed on an inverted wide-field Nikon microscope equipped with a 10× air objective (NA = 0.3) at 37 °C in 5% CO_2_. For all conditions, 61 frames of four fields were acquired using a CCD camera (Coolsnap HQ camera, Photometrics, Tucson, AZ, USA) driven by NIS elements AR 2.30 software (Nikon, Heidelberg, Germany). Migration tracks (n = 60–80 cells) were used to calculate total migration distance, distance to origin, velocity, and directional persistence of cell migration. Velocity was calculated as the total migration distance divided by 600 (600 min is total time of experiment). The distance to origin was determined as the net translocation between the initial and the final position. Directional persistence was calculated as the ratio of the distance to origin to that of the total distance migration.

#### 4.2.13. Molecular Modeling

The ArgusLab 4.0.1 software was used to construct and optimize the binding of compound (**9**) to Tau protein with the semi-empirical AM1 method. The cryo-EM structure of snake Tau conformation in PHFs (Protein Data Bank [PDB] 6QJH) was used in this study. The ligand was superimposed on the tyrosine residue of the sequence ^306^VQIVYK^311^ of the Tau protein. A total of 150 poses of the ligand were generated, and equilibrium binding energies ranged between −0.15 kcal/mol and −6.73 kcal/mol.

#### 4.2.14. Statistical Analysis

Biological data are presented as mean ± s.e.m., while data obtained from proteins in solution are presented as mean ± SD, based on results from at least three independent experiments for all collected data. For cell cycle and mitosis index analysis, the statistical significances of data were analyzed using a nonparametric Mann–Whitney test. Asterisks indicate significance level vs. control (*) *p* < 0.05. For analysis of cell migration and immunofluorescence microscopy of the microtubule network, multiple comparisons were done using one-way ANOVA with a post hoc Tukey–Kramer HSD test, and differences were considered as statistically significant with (**) *p* < 0.01. For quantifications of Western blots, a nonparametric Mann–Whitney test was used to compare independent samples, with differences statistically significant with (*) *p* < 0.05.

## Figures and Tables

**Table 1:** Parameters of aggregation inhibition and equilibrium binding of quercetin (QCT) and compounds (**1**–**17**) to Tau protein.

	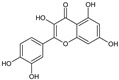 QCT		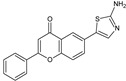 1	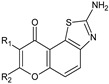 2–15	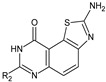 16–17	
Compounds	R_1_	R_2_	Log P ^(a)^	Inhibition of Aggregation IC_50_ ^(b,c)^ (µM)	Equilibrium Binding Constants to Tau ^(a,c)^	
					K_D_ (×10^−6^ M)	f_A_
QCT	-	-	1.68	17.3 ± 0.6	2.4 ± 0.6	0.12 ± 0.04
**1**	-	-	4.17	92.0 ± 9.6	7.0 ± 2.3	0.39 ± 0.08
**2**	H	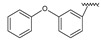	5.46	31.7 ± 7.3	4.7 ± 1.8	0.14 ± 0.10
**3**	H	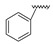	5.05	>100	nd	nd
**4**	H	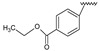	4.28	9.7 ± 0.2	2.4 ± 0.8	0.12 ± 0.01
**5**	H	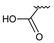	1.62	>100	6.4 ± 1.5	0.38 ± 0.01
**6**	H	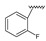	3.85	9.0 ± 0.5	nd	nd
**7**	H	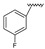	3.87	17.0 ± 2.9	5.9 ± 1.6	0.21 ± 0.02
**8**	H	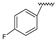	3.89	15.0 ± 1.2	nd	nd
**9**	OH	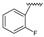	3.55	22.2 ± 4.0	5.5 ± 3.1	0.18 ± 0.04
**10**	OH	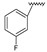	3.58	9.9 ± 1.1	6.3 ± 1.2	0.20 0 ± 0.04
**11**	OH	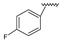	3.60	>100	5.2 ± 1.8	0.16 ± 0.01
**12**	OH	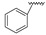	3.44	12.4 ± 1.2	4.7 ± 1.3	0.19 ± 0.05
**13**	OCH_3_	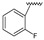	3.83	9.0 ± 0.6	5.4 ± 2.5	0.21 ± 0.02
**14**	OCH_3_	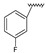	3.85	11.7 ± 2.0	4.2 ± 0.3	0.21 ± 0.04
**15**	OCH_3_	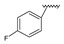	3.88	6.5 ± 0.1	4.1 ± 1.0	0.23 ± 0.03
**16**	-	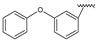	4.82	10.8 ± 2.2	5.0 ± 2.5	0.20 ± 0.03
17	-	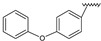	4.85	>100	3.5 ± 1.0	0.14 ± 0.05

^(a)^: the octanol–water partition coefficient log P are predictive values of compounds hydrophobicity; ^(b)^: Drug concentration that inhibits Tau aggregates by 50%; experiments were carried out in buffer A (25 mM of NaPi, 25 mM of NaCl, 0.5 mM of TCEP, 5% DMSO, pH = 6.9 adjusted with NaOH) at 25 °C; ^(c)^: all data are the mean ± SD of three independent experiments; nd: not determined.

**Table 2:** Effects of QCT and compounds (**1**–**17**) on the viability of glioblastoma (U87, U251, U138, and T98G), neuroblastoma (SK-N-SH), and colon adenocarcinoma (Caco-2) cell lines.

Compounds	IC_50_ ^(a)^ (µM)					
	U87	U251	U138	T98G	SK-N-SH	Caco-2
QCT	>100	>100	>100	>100	nd	>100
**1**	22.4 ± 5.2	6.7 ± 2.2	6.4 ± 1.3	4.2 ± 0.7	nd	33.2 ± 13.3
**2**	1.9 ± 0.7	1.7 ± 0.5	2.3 ±1.0	2.8 ± 2.4	1.2 ± 0.3	>100
**3**	>100	>100	>100	>100	>100	>100
**4**	>100	>100	>100	>100	>100	>100
**5**	>100	>100	>100	>100	>100	>100
**6**	2.6 ± 1.3	1.3 ±0.4	1.0 ± 0.4	2.9 ± 0.7	nd	nd
**7**	>100	>100	>100	>100	nd	nd
**8**	>100	3.6 ± 1.1	10.2 ± 5.0	>100	nd	nd
**9**	2.4 ± 1.7	1.7 ± 0.5	2.3 ± 1.0	2.8 ± 2.4	1.2 ± 0.2	>100
**10**	2.6 ± 1.5	1.0 ± 0.4	0.9 ± 0.4	1.5 ± 0.7	nd	nd
**11**	5.1 ± 1.8	1.9 ± 0.2	5.0 ± 1.1	3.5 ± 1.0	nd	nd
**12**	4.1 ± 3.4	nd	nd	1.7 ± 0.9	nd	nd
**13**	7.9 ± 3.4	1.7 ± 0.1	1.5 ± 0.3	9.2 ± 1.2	nd	nd
**14**	1.1 ± 0.2	1.3 ± 0.5	1.4 ±0.2	2.7 ± 0.5	nd	nd
**15**	8.8 ± 3.3	1.9 ± 0.2	4.1 ± 1.6	11.3 ± 4.1	nd	nd
**16**	>100	>100	>100	>100	nd	nd
**17**	5.1 ± 0.8	>100	>100	>100	>100	>100

^(a)^ Drug concentration that inhibits metabolic activity by 50% after cell incubation in culture media for 72 h. Data are the mean ± SEM of three independent experiments; nd: not determined.

**Table 3:** Biological effects of derivatives on U87 shCTRL and U87 shTau cells derived from U87 cell types, and expressing or not Tau protein, respectively, as described in [20].

Compounds	IC_50_ ^(a)^ (µM)	
	U87 shCTRL	U87 shTau
**2**	1.4 ± 0.5	4.7 ± 0.3
**3**	>100	>100
**4**	>100	>100
**5**	>100	>100
**9**	1.6 ± 0.5	>100
**10**	1.2 ± 0.4	1.4 ± 0.6
**11**	0.3 ± 0.2	0.4 ± 0.4
**12**	0.6 ± 0.1	0.5 ± 0.3
**16**	>100	>100
**17**	>100	>100

^(a)^ Drug concentration that inhibits metabolic activity by 50% after cell incubation in culture media for 72 h. Data are the mean ± SEM of three independent experiments.

## Data Availability

The data presented in this study are available on request from the corresponding author. The data are not publicly available due to patent applications pending for several of the compounds studied in this study.

## Supplementary Materials

The following supporting information can be downloaded at: https://www.mdpi.com/article/10.3390/ijms242015050/s1, References [81,82,83,84] are cited in the Supplementary Materials.

## References

[1] Mitchison T., Kirschner M. (1984). Dynamic Instability of Microtubule Growth. Nature.

[2] Franchetti P., Cappellacci L., Grifantini M., Barzi A., Nocentini G., Yang H., O’Connor A., Jayaram H.N., Carrell C., Goldstein B.M. (1995). Furanfurin and Thiophenfurin: Two Novel Tiazofurin Analogs. Synthesis, Structure, Antitumor Activity, and Interactions with Inosine Monophosphate Dehydrogenase. J. Med. Chem..

[3] Li X., He Y., Ruiz C.H., Koenig M., Cameron M.D. (2009). Characterization of Dasatinib and Its Structural Analogs as CYP3A4 Mechanism-Based Inactivators and the Proposed Bioactivation Pathways. Drug Metab. Dispos..

[4] Hu-Lieskovan S., Mok S., Homet Moreno B., Tsoi J., Robert L., Goedert L., Pinheiro E.M., Koya R.C., Graeber T.G., Comin-Anduix B. (2015). Improved Antitumor Activity of Immunotherapy with BRAF and MEK Inhibitors in BRAFV600E Melanoma. Sci. Transl. Med..

[5] Williams A.B., Jacobs R.S. (1993). A Marine Natural Product, Patellamide D, Reverses Multidrug Resistance in a Human Leukemic Cell Line. Cancer Lett..

[6] Sharma P.C., Bansal K.K., Sharma A., Sharma D., Deep A. (2020). Thiazole-Containing Compounds as Therapeutic Targets for Cancer Therapy. Eur. J. Med. Chem..

[7] Khrapunovich-Baine M., Menon V., Yang C.-P.H., Northcote P.T., Miller J.H., Angeletti R.H., Fiser A., Horwitz S.B., Xiao H. (2011). Hallmarks of Molecular Action of Microtubule Stabilizing Agents. J. Biol. Chem..

[8] Kaiser S., John Muller J., Eduardo Froehlich P., Cristina Baggio Gnoatto S., Maria Bergold A. (2013). From Bacteria to Antineoplastic: Epothilones A Successful History. Anti-Cancer Agents Med. Chem.-Anti-Cancer Agents.

[9] Lara-Velazquez M., Al-Kharboosh R., Jeanneret S., Vazquez-Ramos C., Mahato D., Tavanaiepour D., Rahmathulla G., Quinones-Hinojosa A. (2017). Advances in Brain Tumor Surgery for Glioblastoma in Adults. Brain Sci..

[10] De Vleeschouwer S. (2017). Glioblastoma.

[11] Figarella-Branger D., Chappe C., Padovani L., Mercurio S., Colin C., Forest F., Bouvier C. (2013). Glial and glioneuronal tumors in adults and children: Main genetic alterations and towards a histomolecular classification. Bull. Cancer.

[12] Weingarten M.D., Lockwood A.H., Hwo S.Y., Kirschner M.W. (1975). A Protein Factor Essential for Microtubule Assembly. Proc. Natl. Acad. Sci. USA.

[13] Cleveland D.W., Hwo S.Y., Kirschner M.W. (1977). Physical and Chemical Properties of Purified Tau Factor and the Role of Tau in Microtubule Assembly. J. Mol. Biol..

[14] Drubin D.G., Kirschner M.W. (1986). Tau Protein Function in Living Cells. J. Cell Biol..

[15] Goedert M., Jakes R. (1990). Expression of Separate Isoforms of Human Tau Protein: Correlation with the Tau Pattern in Brain and Effects on Tubulin Polymerization. EMBO J..

[16] Fuster-Matanzo A., de Barreda E.G., Dawson H.N., Vitek M.P., Avila J., Hernández F. (2009). Function of Tau Protein in Adult Newborn Neurons. FEBS Lett..

[17] Lebouvier T., Pasquier F., Buée L. (2017). Update on Tauopathies. Curr. Opin. Neurol..

[18] Couchie D., Fages C., Bridoux A.M., Rolland B., Tardy M., Nunez J. (1985). Microtubule-Associated Proteins and in Vitro Astrocyte Differentiation. J. Cell Biol..

[19] Miyazono M., Iwaki T., Kitamoto T., Shin R.-W., Fukui M., Tateishi J. (1993). Widespread Distribution of Tau in the Astrocytic Elements of Glial Tumors. Acta Neuropathol..

[20] Breuzard G., Pagano A., Bastonero S., Malesinski S., Parat F., Barbier P., Peyrot V., Kovacic H. (2019). Tau Regulates the Microtubule-Dependent Migration of Glioblastoma Cells via the Rho-ROCK Signaling Pathway. J. Cell Sci..

[21] Pagano A., Breuzard G., Parat F., Tchoghandjian A., Figarella-Branger D., De Bessa T.C., Garrouste F., Douence A., Barbier P., Kovacic H. (2021). Tau Regulates Glioblastoma Progression, 3D Cell Organization, Growth and Migration via the PI3K-AKT Axis. Cancers.

[22] Hedna R., Kovacic H., Pagano A., Peyrot V., Robin M., Devred F., Breuzard G. (2022). Tau Protein as Therapeutic Target for Cancer? Focus on Glioblastoma. Cancers.

[23] Pawar S., Kumar K., Gupta M.K., Rawal R.K. (2021). Synthetic and Medicinal Perspective of Fused-Thiazoles as Anticancer Agents. Anti-Cancer Agents Med. Chem.-Anti-Cancer Agents.

[24] Gandini A., Bartolini M., Tedesco D., Martinez-Gonzalez L., Roca C., Campillo N.E., Zaldivar-Diez J., Perez C., Zuccheri G., Miti A. (2018). Tau-Centric Multitarget Approach for Alzheimer’s Disease: Development of First-in-Class Dual Glycogen Synthase Kinase 3β and Tau-Aggregation Inhibitors. J. Med. Chem..

[25] Gandini A., Gonçalves A.E., Strocchi S., Albertini C., Janočková J., Tramarin A., Grifoni D., Poeta E., Soukup O., Muñoz-Torrero D. (2022). Discovery of Dual Aβ/Tau Inhibitors and Evaluation of Their Therapeutic Effect on a Drosophila Model of Alzheimer’s Disease. ACS Chem. Neurosci..

[26] Jaiswal S., Mishra S., Torgal S.S., Shengule S. (2018). Neuroprotective Effect of Epalrestat Mediated through Oxidative Stress Markers, Cytokines and TAU Protein Levels in Diabetic Rats. Life Sci..

[27] Shao H., Li X., Hayashi S., Bertron J.L., Schwarz D.M.C., Tang B.C., Gestwicki J.E. (2021). Inhibitors of Heat Shock Protein 70 (Hsp70) with Enhanced Metabolic Stability Reduce Tau Levels. Bioorg. Med. Chem. Lett..

[28] Huang W., Wang Y., Tian W., Cui X., Tu P., Li J., Shi S., Liu X. (2022). Biosynthesis Investigations of Terpenoid, Alkaloid, and Flavonoid Antimicrobial Agents Derived from Medicinal Plants. Antibiotics.

[29] Marena G.D., Dos Ramos M.A.S., Carvalho G.C., Paris J.A., Resende F.A., Corrêa I., Ono G.Y.B., Sousa Araujo V.H., de Camargo B.A.F., Bauab T.M. (2022). Natural Product-Based Nanomedicine Applied to Fungal Infection Treatment: A Review of the Last 4 Years. Phytother. Res..

[30] Matsumura Y., Kitabatake M., Kayano S., Ito T. (2023). Dietary Phenolic Compounds: Their Health Benefits and Association with the Gut Microbiota. Antioxidants.

[31] Selvaraj S., Krishnan U.M. (2021). Vanadium–Flavonoid Complexes: A Promising Class of Molecules for Therapeutic Applications. J. Med. Chem..

[32] Yu K.C., Kwan P., Cheung S.K.K., Ho A., Baum L. (2018). Effects of Resveratrol and Morin on Insoluble Tau in Tau Transgenic Mice. Transl. Neurosci..

[33] Fei X., Wang J., Chen C., Ding B., Fu X., Chen W., Wang C., Xu R. (2019). Eupatilin Inhibits Glioma Proliferation, Migration, and Invasion by Arresting Cell Cycle at G1/S Phase and Disrupting the Cytoskeletal Structure. Cancer Manag. Res..

[34] Sonawane S.K., Uversky V.N., Chinnathambi S. (2021). Baicalein Inhibits Heparin-Induced Tau Aggregation by Initializing Non-Toxic Tau Oligomer Formation. Cell Commun. Signal..

[35] Kumar S., Krishnakumar V.G., Morya V., Gupta S., Datta B. (2019). Nanobiocatalyst Facilitated Aglycosidic Quercetin as a Potent Inhibitor of Tau Protein Aggregation. Int. J. Biol. Macromol..

[36] Cox K., Combs B., Abdelmesih B., Morfini G., Brady S.T., Kanaan N.M. (2016). Analysis of Isoform-Specific Tau Aggregates Suggests a Common Toxic Mechanism Involving Similar Pathological Conformations and Axonal Transport Inhibition. Neurobiol. Aging.

[37] Kanaan N.M., Hamel C., Grabinski T., Combs B. (2020). Liquid-Liquid Phase Separation Induces Pathogenic Tau Conformations in Vitro. Nat. Commun..

[38] Mutai P., Breuzard G., Pagano A., Allegro D., Peyrot V., Chibale K. (2017). Synthesis and Biological Evaluation of 4 Arylcoumarin Analogues as Tubulin-Targeting Antitumor Agents. Bioorg. Med. Chem..

[39] Gao L.-X., Chen W.-Q., Liu Y., Jiang F.-L. (2022). Fluorescent Labeling of Human Serum Albumin by Thiol-Cyanimide Addition and Its Application in the Fluorescence Quenching Method for Nanoparticle–Protein Interactions. Anal. Chem..

[40] Siddiqui S., Ameen F., Jahan I., Nayeem S.M., Tabish M. (2019). A Comprehensive Spectroscopic and Computational Investigation on the Binding of the Anti-Asthmatic Drug Triamcinolone with Serum Albumin. New J. Chem..

[41] Weyl D.A., Murfin D. (1966). Fluorescence of Photo-Degraded Tyrosine Solutions. Nature.

[42] Paranthaman S., Uthaiah C.A., Osmani R.A.M., Hani U., Ghazwani M., Alamri A.H., Fatease A.A., Madhunapantula S.V., Gowda D.V. (2022). Anti-Proliferative Potential of Quercetin Loaded Polymeric Mixed Micelles on Rat C6 and Human U87MG Glioma Cells. Pharmaceutics.

[43] Ersoz M., Erdemir A., Derman S., Arasoglu T., Mansuroglu B. (2020). Quercetin-Loaded Nanoparticles Enhance Cytotoxicity and Antioxidant Activity on C6 Glioma Cells. Pharm. Dev. Technol..

[44] Hasan A.A.S., Kalinina E.V., Tatarskiy V.V., Volodina Y.L., Petrova A.S., Novichkova M.D., Zhdanov D.D., Shtil A.A. (2022). Suppression of the Antioxidant System and PI3K/Akt/MTOR Signaling Pathway in Cisplatin-Resistant Cancer Cells by Quercetin. Bull. Exp. Biol. Med..

[45] Mosmann T. (1983). Rapid Colorimetric Assay for Cellular Growth and Survival: Application to Proliferation and Cytotoxicity Assays. J. Immunol. Methods.

[46] Hashemzaei M., Delarami Far A., Yari A., Heravi R.E., Tabrizian K., Taghdisi S.M., Sadegh S.E., Tsarouhas K., Kouretas D., Tzanakakis G. (2017). Anticancer and Apoptosis-inducing Effects of Quercetin in Vitro and in Vivo. Oncol. Rep..

[47] Sang D., Li R., Lan Q. (2014). Quercetin Sensitizes Human Glioblastoma Cells to Temozolomide in Vitro via Inhibition of Hsp27. Acta Pharmacol. Sin..

[48] Abolibda T.Z., Fathalla M., Farag B., Zaki M.E.A., Gomha S.M. (2023). Synthesis and Molecular Docking of Some Novel 3-Thiazolyl-Coumarins as Inhibitors of VEGFR-2 Kinase. Molecules.

[49] Ye Y., Huang Z., Zhang M., Li J., Zhang Y., Lou C. (2023). Synergistic Therapeutic Potential of Alpelisib in Cancers (Excluding Breast Cancer): Preclinical and Clinical Evidences. Biomed. Pharmacother..

[50] Ivasechko I., Lozynskyi A., Senkiv J., Roszczenko P., Kozak Y., Finiuk N., Klyuchivska O., Kashchak N., Manko N., Maslyak Z. (2023). Molecular Design, Synthesis and Anticancer Activity of New Thiopyrano [2,3-d]Thiazoles Based on 5-Hydroxy-1,4-Naphthoquinone (Juglone). Eur. J. Med. Chem..

[51] Tanida I., Ueno T., Kominami E. (2008). LC3 and Autophagy. Methods Mol. Biol..

[52] Gibellini L., Bianchini E., De Biasi S., Nasi M., Cossarizza A., Pinti M. (2015). Natural Compounds Modulating Mitochondrial Functions. Evid.-Based Complement. Altern. Med. ECAM.

[53] Kicinska A., Jarmuszkiewicz W. (2020). Flavonoids and Mitochondria: Activation of Cytoprotective Pathways?. Molecules.

[54] Ostlund R.E., Leung J.T., Hajek S.V. (1979). Biochemical Determination of Tubulin-Microtubule Equilibrium in Cultured Cells. Anal. Biochem..

[55] Panda D., Samuel J.C., Massie M., Feinstein S.C., Wilson L. (2003). Differential Regulation of Microtubule Dynamics by Three- and Four-Repeat Tau: Implications for the Onset of Neurodegenerative Disease. Proc. Natl. Acad. Sci. USA.

[56] Von Bergen M., Friedhoff P., Biernat J., Heberle J., Mandelkow E.M., Mandelkow E. (2000). Assembly of Tau Protein into Alzheimer Paired Helical Filaments Depends on a Local Sequence Motif ((306)VQIVYK(311)) Forming Beta Structure. Proc. Natl. Acad. Sci. USA.

[57] Von Bergen M., Barghorn S., Li L., Marx A., Biernat J., Mandelkow E.M., Mandelkow E. (2001). Mutations of Tau Protein in Frontotemporal Dementia Promote Aggregation of Paired Helical Filaments by Enhancing Local Beta-Structure. J. Biol. Chem..

[58] Li W., Lee V.M.-Y. (2006). Characterization of Two VQIXXK Motifs for Tau Fibrillization in Vitro. Biochemistry.

[59] Zhang W., Falcon B., Murzin A.G., Fan J., Crowther R.A., Goedert M., Scheres S.H. (2019). Heparin-Induced Tau Filaments Are Polymorphic and Differ from Those in Alzheimer’s and Pick’s Diseases. eLife.

[60] Moreno-Castillo E., Álvarez-Ginarte Y.M., Valdés-Tresanco M.E., Montero-Cabrera L.A., Moreno E., Valiente P.A. (2020). Understanding the Disrupting Mechanism of the Tau Aggregation Motif “306VQIVYK311” by Phenylthiazolyl-Hydrazides Inhibitors. J. Mol. Recognit..

[61] Viswanathan G.K., Shwartz D., Losev Y., Arad E., Shemesh C., Pichinuk E., Engel H., Raveh A., Jelinek R., Cooper I. (2020). Purpurin Modulates Tau-Derived VQIVYK Fibrillization and Ameliorates Alzheimer’s Disease-like Symptoms in Animal Model. Cell. Mol. Life Sci..

[62] Haj E., Losev Y., Guru KrishnaKumar V., Pichinuk E., Engel H., Raveh A., Gazit E., Segal D. (2018). Integrating in Vitro and in Silico Approaches to Evaluate the “Dual Functionality” of Palmatine Chloride in Inhibiting and Disassembling Tau-Derived VQIVYK Peptide Fibrils. Biochim. Biophys. Acta BBA - Gen. Subj..

[63] Annadurai N., Malina L., Salmona M., Diomede L., Bastone A., Cagnotto A., Romeo M., Šrejber M., Berka K., Otyepka M. (2022). Antitumour Drugs Targeting Tau R3 VQIVYK and Cys322 Prevent Seeding of Endogenous Tau Aggregates by Exogenous Seeds. FEBS J..

[64] Levy J.M.M., Towers C.G., Thorburn A. (2017). Targeting Autophagy in Cancer. Nat. Rev. Cancer.

[65] Kocaturk N.M., Akkoc Y., Kig C., Bayraktar O., Gozuacik D., Kutlu O. (2019). Autophagy as a Molecular Target for Cancer Treatment. Eur. J. Pharm. Sci..

[66] Jin S.M., Lazarou M., Wang C., Kane L.A., Narendra D.P., Youle R.J. (2010). Mitochondrial Membrane Potential Regulates PINK1 Import and Proteolytic Destabilization by PARL. J. Cell Biol..

[67] Hu Y., Li X.-C., Wang Z., Luo Y., Zhang X., Liu X.-P., Feng Q., Wang Q., Yue Z., Chen Z. (2016). Tau Accumulation Impairs Mitophagy via Increasing Mitochondrial Membrane Potential and Reducing Mitochondrial Parkin. Oncotarget.

[68] Cummins N., Tweedie A., Zuryn S., Bertran-Gonzalez J., Götz J. (2019). Disease-Associated Tau Impairs Mitophagy by Inhibiting Parkin Translocation to Mitochondria. EMBO J..

[69] Nabti I., Reddy B.J.N., Rezgui R., Wang W., Gross S.P., Shubeita G.T. (2022). The Ubiquitous Microtubule-Associated Protein 4 (MAP4) Controls Organelle Distribution by Regulating the Activity of the Kinesin Motor. Proc. Natl. Acad. Sci. USA.

[70] Nishida K., Matsumura K., Tamura M., Nakamichi T., Shimamori K., Kuragano M., Kabir A.M.R., Kakugo A., Kotani S., Nishishita N. (2023). Effects of Three Microtubule-Associated Proteins (MAP2, MAP4, and Tau) on Microtubules’ Physical Properties and Neurite Morphology. Sci. Rep..

[71] Doki C., Nishida K., Saito S., Shiga M., Ogara H., Kuramoto A., Kuragano M., Nozumi M., Igarashi M., Nakagawa H. (2020). Microtubule Elongation along Actin Filaments Induced by Microtubule-Associated Protein 4 Contributes to the Formation of Cellular Protrusions. J. Biochem..

[72] Tardivel M., Bégard S., Bousset L., Dujardin S., Coens A., Melki R., Buée L., Colin M. (2016). Tunneling Nanotube (TNT)-Mediated Neuron-to Neuron Transfer of Pathological Tau Protein Assemblies. Acta Neuropathol. Commun..

[73] Needs H.I., Wilkinson K.A., Henley J.M., Collinson I. (2023). Aggregation-Prone Tau Impairs Mitochondrial Import, Which Affects Organelle Morphology and Neuronal Complexity. J. Cell Sci..

[74] Breuzard G., Hubert P., Nouar R., Bessa T.D., Devred F., Barbier P., Sturgis J.N., Peyrot V. (2013). Molecular Mechanisms of Tau Binding to Microtubules and Its Role in Microtubule Dynamics in Live Cells. J. Cell Sci..

[75] Chou T.C., Talalay P. (1984). Quantitative Analysis of Dose-Effect Relationships: The Combined Effects of Multiple Drugs or Enzyme Inhibitors. Adv. Enzyme Regul..

[76] De Bessa T., Breuzard G., Allegro D., Devred F., Peyrot V., Barbier P., Smet-Nocca C. (2017). Tau Interaction with Tubulin and Microtubules: From Purified Proteins to Cells. Tau Protein: Methods and Protocols.

[77] Lakowicz J.R. (2006). Principles of Fluorescence Spectroscopy.

[78] Watson J., Chambers S., Smith P. (1987). A pragmatic approach to the analysis of DNA histograms with a definable G1 peak. Cytometry.

[79] Cummings J., Zelcer N., Allen J.D., Yao D., Boyd G., Maliepaard M., Friedberg T.H., Smyth J.F., Jodrell D.I. (2004). Glucuronidation as a Mechanism of Intrinsic Drug Resistance in Colon Cancer Cells: Contribution of Drug Transport Proteins. Biochem. Pharmacol..

[80] Schobert R., Effenberger-Neidnicht K., Biersack B. (2011). Stable Combretastatin A-4 Analogues with Sub-Nanomolar Efficacy against Chemoresistant HT-29 Cells. Int. J. Clin. Pharmacol. Ther..

[81] Agrawal N.N., Soni P.A. (2005). Reaction of 2′-Hydroxy-5′-Acetamido Chalcones with Dimethyl Sulfoxide-Iodine, Pyridine-Mercuric (II) Acetate and Triethanolamine.

[82] Raval A.A., Shah N.M. (1957). Chalcones and Related Compounds Derived from 2-Hydroxy-5-Acetaminoacetophenone II. Flavones and Flavonols. J. Org. Chem..

[83] Yap S., Loft K.J., Woodman O.L., Williams S.J. (2008). Discovery of Water-Soluble Antioxidant Flavonols without Vasorelaxant Activity. ChemMedChem.

[84] Tang J.-H., Shi D.-X., Zhang L.-J., Zhang Q., Li J.-R. (2010). Facile and One-Pot Synthesis of 1,2-Dihydroquinazolin-4(3H)-Ones via Tandem Intramolecular Pinner/Dimroth Rearrangement. Synth. Commun..

