# Supplementary material for: 2-Aminothiazole-Flavonoid Hybrid Derivatives Binding to Tau Protein and Responsible for Antitumor Activity in Glioblastoma
Source: Int J Mol Sci. 2023 Oct 10;24(20):15050. doi: 10.3390/ijms242015050 (PMC10606064; doi:10.3390/ijms242015050)

**Supplementary Materials File S1 — Supplementary materials and methods**

# Thiazole-flavonoid hybrid derivatives binding to Tau protein and responsible for antitumor activity in glioblastoma

Rayane Hedna [1], Attilio DiMaio [2], Maxime Robin [2], Diane Allegro [1], Mario Tatoni [1], Vincent Peyrot [1], Pascale Barbier [1], Hervé Kovacic [1], Gilles Breuzard [1, *]

1 - Faculté des Sciences Médicales et Paramédicales, Institut de Neurophysiopathologie (INP), UMR 7051, CNRS, Aix Marseille Université, 13005 Marseille, France

2 - Faculté de Pharmacie, Institut Méditerranéen de Biodiversité et Ecologie marine et continentale (IMBE), UMR 7263, CNRS, IRD 237, Aix-Marseille Université, 13005 Marseille, France

* Correspondence: gilles.breuzard@univ-amu.fr

**Flavone derivatives Synthetic protocols Compounds 2-15:**

**Synthesis of chalcones (i)**

A solution of 2-hydroxy-5-acetamidoacetophenone (1.93 g, 10 mmol), benzaldehyde (10 mmol) and $LiOH.H_2O$ (2.94 g, 70 mmol) in MeOH (20 mL) was submitted to MicroWave Irradiation (MWI) in a CEM Apparatus. MWI Open vessel, power: 300 watts, solvent MeOH, T°= 80°C, 2 min and 20 min. The colorless solution was cooled to r.t., MeOH was removed under vaccum and the mixture was poured into 1N HCl (50 mL). The precipitate obtained was then filtered off, washed with excess water and dried to give chalcone.

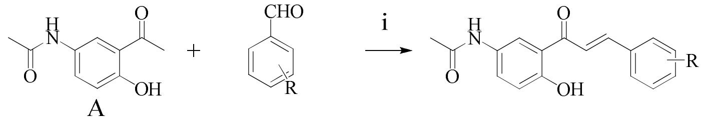


Example1: (E)-*N*-(3-cinnamoyl-4-hydroxyphenyl)acetamide

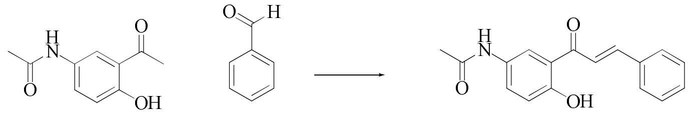


The yellow precipitate obtained was then filtered off, washed with water and dried (2.3 g, 82%, mp: 190°C) [81, 82]. $^1$H NMR (300 MHz, DMSO-$d_6$) δ 2.03 (s, 3H, $CH_3$), 6.96 (d, 1H, $J$ = 8.84 Hz), 7.47-7.50 (m, 3H), 7.68-7.73 (dd, 1H, $J$ = 2.5, 8.8 Hz), 7.78-7.84 (m, 4H), 8;15 (d, 1H, J = 2.5 Hz), 9.89 (s, 1H, OH), 11.70 (s, 1H, NH). $^{13}$C NMR (75 MHz, DMSO-$d_6$) δ 23.70 (15), 117.71 (5), 120.99 (2), 121.28 (3), 122.75 (8), 128.07 (13), 128.77 (11), 129.08 (12), 130.93 (6), 131.07 (1), 134.45 (10), 144.13 (9), 156.71 (4), 168.13 (14), 192.87 (7). Anal. Calcd. for $C_{17}H_{15}NO_3$: C, 72.58; H, 5.37; N, 4.98. Found : C, 72.01; H, 5.51; N, 4.89.

**Synthesis of flavones (ii)**

Chalcone (10 mmol), $I_2$ (0.28 g, 10% wt) were solubilized in DMSO (10 mL). The solution was submitted to MWI at 140°C (DMSO, 2+20 min, open vessel). The solution was cooled to r.t. and poured onto cold 1N HCl solution (100 mL). The solution was stirred for 1 h and the orange precipitate was then dilute with ice water (100 mL) and filtered off. The precipitate collected was washed with excess water to removed DMSO. The precipitate obtained was filtered off to give various chalcone and used without further purification.

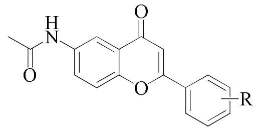


Example : *N*-(4-oxo-2-phenyl-4*H*-chromen-6-yl)acetamide

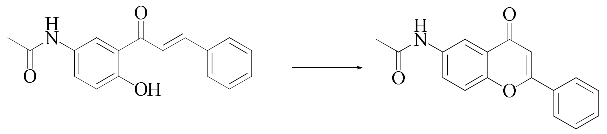


(E)-*N*-(3-cinnamoyl-4-hydroxyphenyl)acetamide (2.81 g 10 mmol), $I_2$ (0.28 g, 10% wt) were solubilized in DMSO (10 mL). The solution was submitted to MWI at 140°C (DMSO, 2+20 min, open vessel). The solution was cooled to r.t. and poured onto cold 1N HCl solution (100

mL). The solution was stirred for 1 h and the orange precipitate was then dilute with ice water (100 mL) and filtered off. The precipitate collected was washed with excess water to removed DMSO. An orange powder was obtained (2.7 g, 96%, mp: 280°C) [83]. $^1$H NMR (300 MHz, DMSO-d$_6$) δ 2.09 (s, 3H, $CH_3$), 7.00 (s, 1H), 7.56-7.61 (m, 3H), 7.73-7.76 (d, 1H, *J* = 9.0 Hz), 7.94-7.98 (dd, 1H, J = 2.7, 9.0 Hz), 8.07-8.11 (m, 2H), 8.33 (d, 1H, *J* = 2.7 Hz), 10.27 (s, 1H, NH). $^{13}$C NMR (75 MHz, DMSO-d$_6$) δ 23.98 (11), 106.42 (7), 113.01 (1), 119.01 (4), 123.50 (8a), 125.56 (3), 126.29 (2'), 129.11 (3'), 131.21 (1'), 131.74 (4'), 136.79 (2), 151.49 (4a), 162.34 (6), 168.55 (10), 176.98 (8). Anal. Calcd. for $C_{17}H_{13}NO_3$: C, 73.11; H, 4.69; N, 5.02. Found : C, 72.98; H, 4.84; N, 5.01.

**Synthesis of flavonols (iii)**

Chalcone (10 mmol) was solubilised in EtOH (50 mL), then was added NaOH 5% (40 mL) and $H_2O_2$ 25 % (10 mL). The solution was stirred overnight at r.t. and put onto water (100 mL) and acidified with 2M HCl. The yellow precipitate obtained was filtered, washed with water and dried to give corresponding flavonols.

Example: **N-(3-hydroxy-4-oxo-2-phenyl-4H-chromen-6-yl)acetamide [83]**

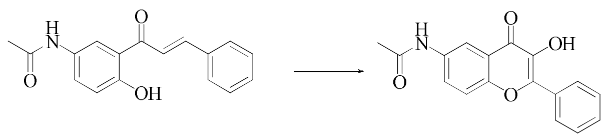


Yellow solid, yield: 41%, Mol.Wt : 295, Anal. Calc. for $C_{17}H_{13}NO_4$: C, 69.15; H, 4.44; N, 4.74. Found: C, 69.12; H, 4.48; N, 4.68. $^1$H NMR (300MHz, DMSO-*d$_6$*) δ ppm: 2.08 (s, 3H, $CH_3$), 7.51 (m, 1H, $H_{4'}$), 7.55 (m, 2H, $H_{3'}$), 7.71 (d, 1H, J=9.2Hz, $H_4$), 7.90 (dd, 1H, J=2.5Hz, 9.2Hz, $H_3$), 8.19 (m, 2H, $H_{2'}$), 8.42 (d, 1H, J=2.5Hz, $H_1$), 9.53 (s, OH), 10.30 (s, NH). $^{13}$C NMR (75 MHz, DMSO-*d$_6$*) δ ppm 24.09 (CH3), 112.77 (1), 119.08 (4), 121.48 (8a), 125.74 (3), 127.75 (2'), 128.68 (3'), 130.02 (1'), 131.43 (4'), 136.05 (2), 138.87 (7), 145.25 (6), 150.76 (4a), 168.84 (6'), 172.96 (8).

**General procedure for methylation of Flavonol (iv)**

Flavonol (10 mmol) was solubilzed in acetone (20 mL) with $K_2CO_3$ (1.eq), then was introduced Dimethyl sulphate (1eq) and the solution was heated at 50°C for 6h. The reaction was monitored by TLC and when no more beginning product appeared, the solvent was removed under reduce pressure. The mixture obtained was poured onto water (100 mL) and the pH was adjusted to 7. The precipitate obtained was filtered off and washed with water and dried to afford methoxyflavone.

Yellow solid, yield: 86%, Mol.Wt : 309, Anal. Calc. for $C_{18}H_{15}NO_4$: C, 69.89; H, 4.89; N, 4.53. Found: C, 69.83; H, 4.92; N, 4.49. $^1$H NMR (300MHz, DMSO-*d$_6$*) δ ppm: 2.08 (s, 3H, $CH_3$), 3.81 (s, 3H, $OCH_3$), 7.56 (m, 2H, $H_{3'}$), 7.58 (m, 1H, $H_{4'}$), 7.67 (d, 1H, J=9.1Hz, $H_4$), 7.94 (dd, 1H, J=9.1, 2.5Hz, $H_3$), 8.02 (m, 2H, $H_{2'}$), 8.37 (d, 1H, J=2.5Hz, $H_1$), 10.28 (s, 1H, NH). $^{13}$C NMR (75 MHz, DMSO-*d$_6$*) δ ppm 23.96(11), 59.65(12), 112.84(1), 118.87(4), 123.68(8a), 125.55(3), 128.19(2'), 128.62(3'), 130.5(1'), 130.77(4'), 136.38(2), 140.41(6), 150.61(4a), 154.77(7), 168.54(10), 173.68(8).

**General procedure for deprotection of various acetamido derivatives (v)**

Flavone, Flavonol or MethoxyFlavone (10 mmol) was added to a solution of EtOH (20 mL) and $H_2SO_4$ conc. (5 mL). The solution was submitted to MWI in open vessel 80°C, 2+20 min, upon cooling the solvent was removed under vacuo and the residue obtain is poured onto ice water (100 mL). Then the solution was neutralized with $NH_4OH$ 16% until pH=7. The red precipitate formed was collected by filtration and washed with excess cold water. Amino compound was obtained as a powder without further purification.

Example 1: 6-amino-2-phenyl-4*H*-chromen-4-one

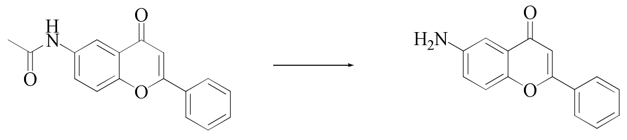

Red powder obtained without further purification (2.3 g, 96%, mp: 201°C)[3]. $^1$H NMR (300 MHz, DMSO-$d_6$) δ 6.87 (s, 1H), 7.07-7.11 (dd, 1H, *J* = 2.7, 8.8 Hz), 7.13 (d, 1H, *J* = 2.7 Hz), 7.50-7.53 (d, 1H, *J* = 8.8 Hz), 7.56-7.58 (m, 3H), 8.03-8.06 (m, 2H). $^{13}$C NMR (75 MHz, DMSO-$d_6$) δ 105.24 (1), 105.77 (7), 119.03 (4), 121.91 (3), 124.23 (8a), 126.11 (2'), 129.08 (3'), 131.43 (1'), 131.60 (4'), 146.23 (2), 148.08 (4a), 161.70 (6), 177.14 (8). Anal. Calcd. for $C_{15}H_{11}NO_2$: C, 75.94; H, 4.67; N, 5.90. Found : C, 75.77; H, 4.97; N, 5.58.

Example 2: 6-amino-3-hydroxy-2-phenyl-4H-chromen-4-one

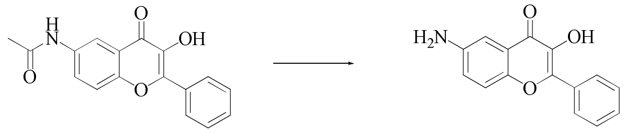

White solid, yield: 94%, Mol.Wt : 253, Anal. Calc. for $C_{15}H_{11}NO_3$: C, 71.14; H, 4.38; N, 5.53. Found: C, 71.11; H, 4.42; N, 5.49. $^1$H NMR (300MHz, DMSO-*$d_6$*) δ ppm: 7.10 (dd, 1H, J=8.9, 2.6Hz, $H_3$), 7.18 (d, 1H, J=2.6Hz, $H_1$), 7.48 (d, 1H, J=8.9Hz, $H_4$), 7.50 (m, 1H, $H_{4'}$), 7.55 (m, 2H, $H_{3'}$), 8.17 (m, 2H, $H_{2'}$), 9.27 (s, 1H, OH).

Example 3: 6-amino-3-methoxy-2-phenyl-4H-chromen-4-one

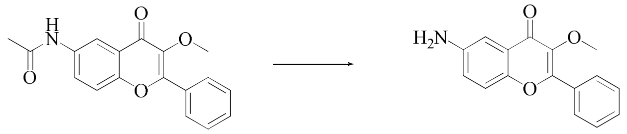

Yellow solid, yield: 80%, Mol.Wt : 267, Anal. Calc. for $C_{16}H_{13}NO_3$: C, 71.90; H, 4.90; N, 5.24. Found: C, 71.88; H, 4.92; N, 5.21. $^1$H NMR (300MHz, DMSO-*$d_6$*) δ ppm: 3.78 (s, 3H, $OCH_3$), 7.13 (dd, 1H, J=8.9, 2.6Hz, $H_3$), 7.21 (d, 1H, J=2.6Hz, $H_1$), 7.48 (d, 1H, J=8.9Hz, $H_4$), 7.57 (m, 1H, $H_{4'}$), 7.58 (m, 2H, $H_{3'}$), 8.00 (m, 2H, $H_{2'}$).

**General procedure for preparation of 2-aminothiazole moiety with various amino compounds (vi).**

Amino derivatives (5 mmol) and KSCN (0.73 g, 7.5 mmol) were stirred in AcOH (40 mL) at rt for 30 min. A solution of bromine (5 mmol) in 10 mL of AcOH was added dropwise to the

mixture under vigorous stirring at rt for 2h. During the course of the reaction a precipitate was formed. At that time, water was added (20 mL) and the pH was adjust to 4-5 with NH4OH 16%. The resulting colorless precipitate was filtered off and dried affording Thiazoloderivatives.

**1- 6-(2-aminothiazol-4-yl)-2-phenyl-4H-chromen-4-one**

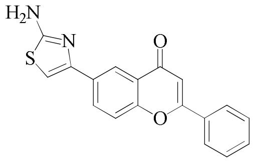


Mol. Wt.: 320; Anal. Calc. for $C_{18}H_{12}N_2O_2S$: C, 67.48; H, 3.78; N, 8.74. Found: C, 67.32; H, 3.79; N, 8.70. m/z: 320,3651 (100,0%). $^{1}$H NMR (300 MHz, DMSO-$d_6$) δ ppm 7.10 (s, 1H, C-$H_3$), 7.44 (s, 1H, C-$H_{thiazol}$), 7.59-7.63 (m, 3 H), 7.94 (d, 1H, C-$H_8$), 8.13 (d, 2H, C-$H_{phenyl}$), 8.43(dd, 1H, $J$ = 2.7, 8.8 Hz, C-$H_7$), 8.63 (d, 1H, $J$ = 2.3 Hz, C-$H_5$). $^{13}$C NMR (75 MHz, DMSO-$d_6$) δ ppm 101.94 (C-thiazol), 104.45 (C-3), 118.23 (C-8), 121.65 (C-4a), 127.76 (C-6), 127.92 (C-2'), 128.87 (C-4'), 130.54 (C-1'), 131.09 (C-5), 131.45 (C-3'), 134.23 (C-7), 150.12 (C-thiazol), 152.45 (C-8a), 162.67 (C-2), 164.45 (C-thiazol), 177.54 (C-4).

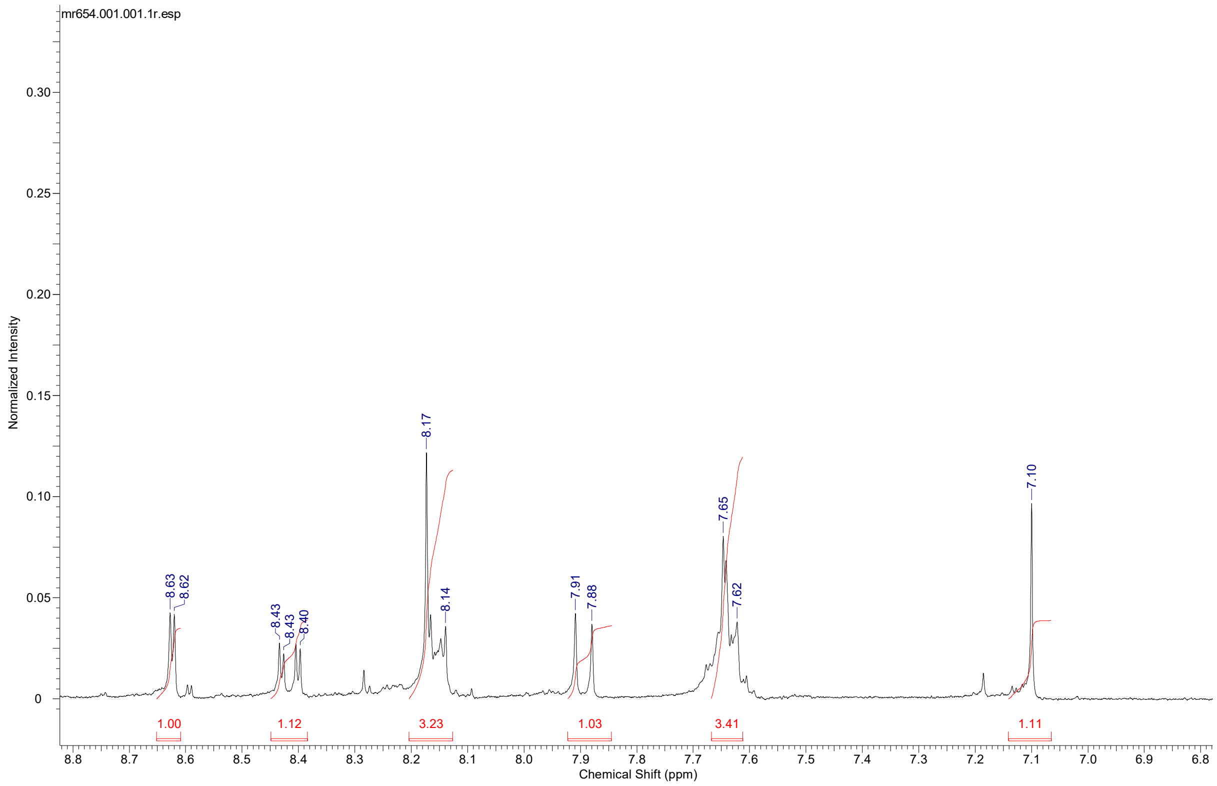


**2- MRCG38 2-amino-7-(3-phenoxyphenyl)-9H-chromeno[6,5-d]thiazol-9-one**

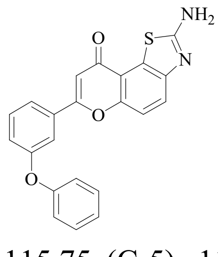


Mol. Wt.: 386; Anal. Calc. for $C_{22}H_{14}N_2O_3S$: C, 68.38; H, 3.65; N, 7.25. Found: C, 68.23; H, 3.75; N, 7.09. m/z: 386,07 (100,0%).

$^{1}$H NMR (300 MHz, DMSO-$d_6$) δ ppm 7.09 (brd, 2H, $J$=7.6Hz, C-$H_{2''}$), 7.15 (s, 1H, C-$H_8$), 7.18 (m, 1H, C-$H_{4'}$), 7.20 (m, 1H, C-$H_{4''}$), 7.43 (brt, 2H, $J$=7.6Hz, C-$H_{3''}$), 7.59 (t, 1H, $J$=7.9Hz, C-$H_{5'}$), 7.67 (d, 1H, $J$=8.9Hz, C-$H_5$), 7.77 (d, 1H, $J$=8.9Hz, C-$H_4$), 7.82 (d, 1H, $J$=2.1Hz, C-$H_{2'}$), 7.91 (brd, 1H, $J$=7.7Hz, C-$H_{6'}$). $^{13}$C NMR (75 MHz, DMSO-$d_6$) δ ppm 106.45 (C-8), 115.75 (C-5), 117.86 (C-9a), 118.68 (C-2'), 118.76 (C-2"), 121.55 (C-4'), 121.55 (C-6'), 123.15 (C-4), 123.86 (C-4"), 124.47 (C-9b), 130.21 (C-3"), 130.90 (C-5'), 133.29 (C-1'), 151.02 (C-5a), 151.02 (C-3a), 156.34 (C-1"), 157.24 (C-3'), 161.80 (C-7), 170.22 (C-2), 176.24 (C-9).

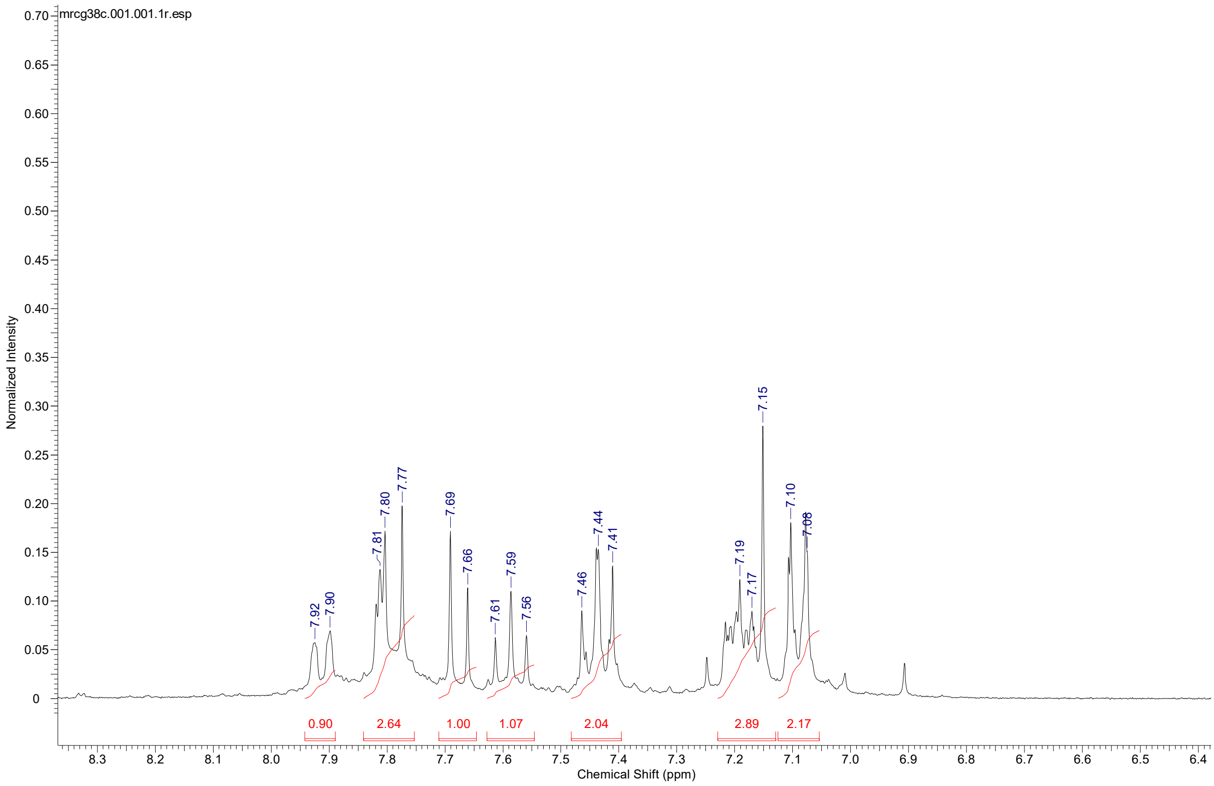


**3- 2-amino-7-phenyl-9H-chromeno[6,5-d]thiazol-9-one (MR413)**

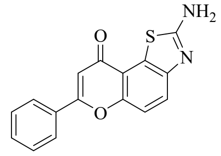


Brown solid, yield 72 %, Mol.Wt.: 294.33, Anal. Calcd. for $C_{16}H_{10}N_2O_2S$: C, 65.29; H, 3.42; N, 9.52. Found : C, 64.78; H, 3.56; N, 9.34. m/z: 294,05 (100,0%). $^1$H NMR (300 MHz, DMSO-$d_6$) δ ppm 7.14 (s, 1H), 7.59-7.63 (m, 3 H), 7.69 (d, *J*=8.8 Hz, 1 H) 7.81 (d, *J*=8.8 Hz, 1 H), 8.12 (d, 1 H). $^{13}$C NMR (75 MHz, DMSO-$d_6$) δ ppm 105.94 (8), 115.51 (5), 117.86 (9a), 123.37 (4), 124.93 (9b), 126.37 (2'), 129.15 (3'), 131.26 (1'), 131.79 (4'), 150.78 (3a), 150.97 (5a), 162.59 (7), 170.15 (2), 176.25 (9).

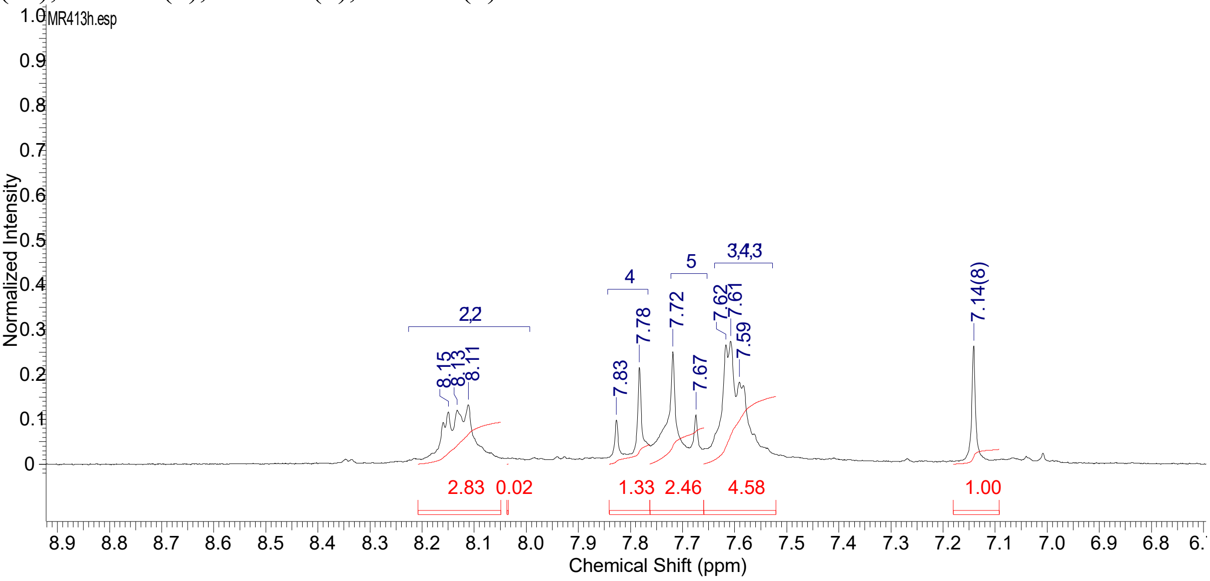

**4- MRCG50 ethyl 4-(2-amino-9-oxo-9H-chromeno[6,5-d]thiazol-7-yl)benzoate**

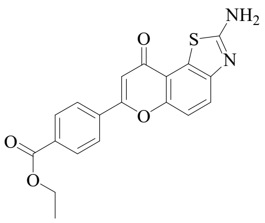

Mol. Wt.: 366; Anal. Calc. for $C_{19}H_{14}N_2O_4S$: C, 62.28; H, 3.85; N, 7.65. Found: C, 62.12; H, 3.84; N, 7.56. m/z: 366,07 (100,0%).

$^{1}$H NMR (300 MHz, DMSO-*d*$_6$) δ ppm 1.33 (t, 3H, *J*=7.0Hz, C-H$_{13}$), 4.30 (q, 2H, *J*=7.0Hz, C-H$_{12}$), 7.21 (s, 1H, C-H$_8$), 7.63 (bs, 2H, NH2), 7.73 (d, 1H, *J*=8.9Hz, C-H$_5$), 7.80 (d, 1H, *J*=8.9Hz, C-H$_4$), 8.14 (d, 2H, *J*=8.7Hz, C-H$_{2'}$), 8.28 (d, 2H, *J*=8.7 Hz, C-H$_{3'}$). $^{13}$C NMR (75 MHz, DMSO-*d*$_6$) δ ppm 14.27 (C-13), 61.32 (C-12), 107.33 (C-8), 115.74 (C-5), 118.02 (C-9a), 123.57 (C-4), 124.96 (C-9b), 126.71 (C-2'), 129.75 (C-3'), 132.32 (C-4'), 135.46 (C-1'), 150.63 (C-5a), 151.11 (C-3a), 161.30 (C-7), 165.12 (C-10), 170.39 (C-2), 176.32 (C-9).

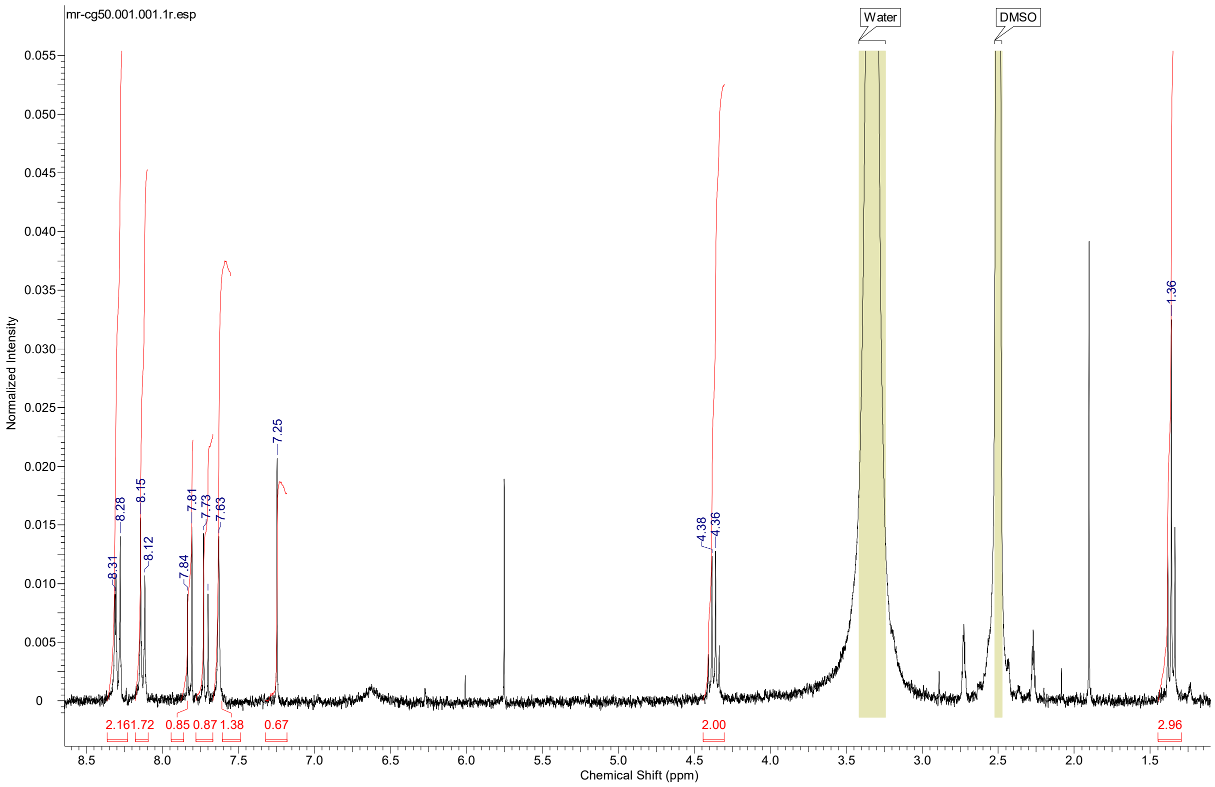


**5- MR459 2-amino-9-oxo-9H-chromeno[6,5-d]thiazole-7-carboxylic acid**

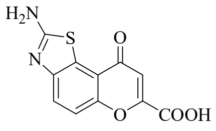

Mol. Wt.: 262; Anal. Calc. for $C_{11}H_6N_2O_4S$: C, 50.38; H, 2.31; N, 10.68. Found: C, 50.02; H, 2.36; N, 10.44. m/z: 262,00 (100,0%).

$^{1}$H NMR (300 MHz, DMSO-*d*$_6$) δ ppm 6.81 (1H, s, C-H$_8$), 7.58 (1H, d, *J*=8.87Hz, C-H$_5$), 7.58 (2H, s, NH2), 7.79 (1H, d, *J*=8.87Hz, C-H$_4$). $^{13}$C NMR (75 MHz, DMSO-*d*$_6$) δ ppm 117.3 (C-5), 118.8 (C-9a), 119.2 (C-3a), 122.80 (C-8), 128.30 (C-4), 154,5 (C-5a), 157.70 (C-9b), 160.90 (C-7), 164.50 (COOH), 166.30 (C-2), 182.10 (C-9)

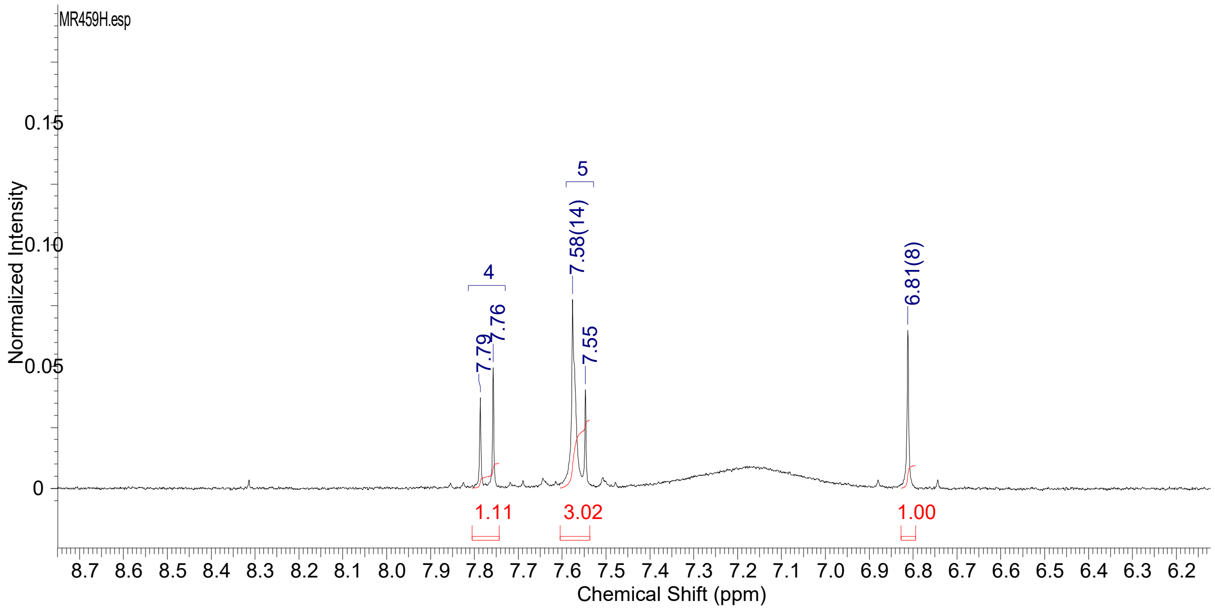


**6- MRCG18 2-amino-7-(2-fluorophenyl)-9H-chromeno[6,5-d]thiazol-9-one**

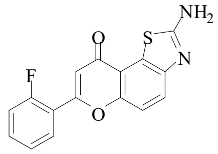


Mol. Wt.: 312; Anal. Calc. for $C_{16}H_9FN_2O_2S$: C, 61.53; H, 2.90; N, 8.97. Found: C, 61.47; H, 2.95; N, 8.87. m/z: 312.04 (100.0%).

$^1$H NMR (300 MHz, DMSO-$d_6$) δ ppm 6.85 (s, 1H, C-$H_8$), 7.42 (brd, 1H, *J*=8.5 Hz, C-$H_{5'}$), 7.47 (brd, 1H, *J*=8.5 Hz, C-$H_{3'}$), 7.62 (brd, 4H, C-$H_{5,4'}$ $NH_2$), 7.78 (d, 1H, *J*=8.6 Hz, C-$H_4$), 8.04 (t, 1H, *J*=7.5 Hz, C-$H_{6'}$).

$^{13}$C NMR (75 MHz, DMSO-$d_6$) δ ppm 110.60 (d, J=8.3Hz, C-8), 115.68 (C-5), 117.05 (d, J=22.6Hz, C-3'), 117.83 (C-9a), 119.92 (d, J=14.3Hz, C-1'), 123.73 (C-4), 125.08 (C-9b), 125.40 (C-5'), 129.82 (C-6'), 133.83 (d, J=6.8Hz, C-4'), 150.96 (C-5a), 151.33 (C-3a), 158.82 (C-7), 159.95 (d, J=248.00Hz, C-2'), 170.37 (C-2), 176.15 (C-9).

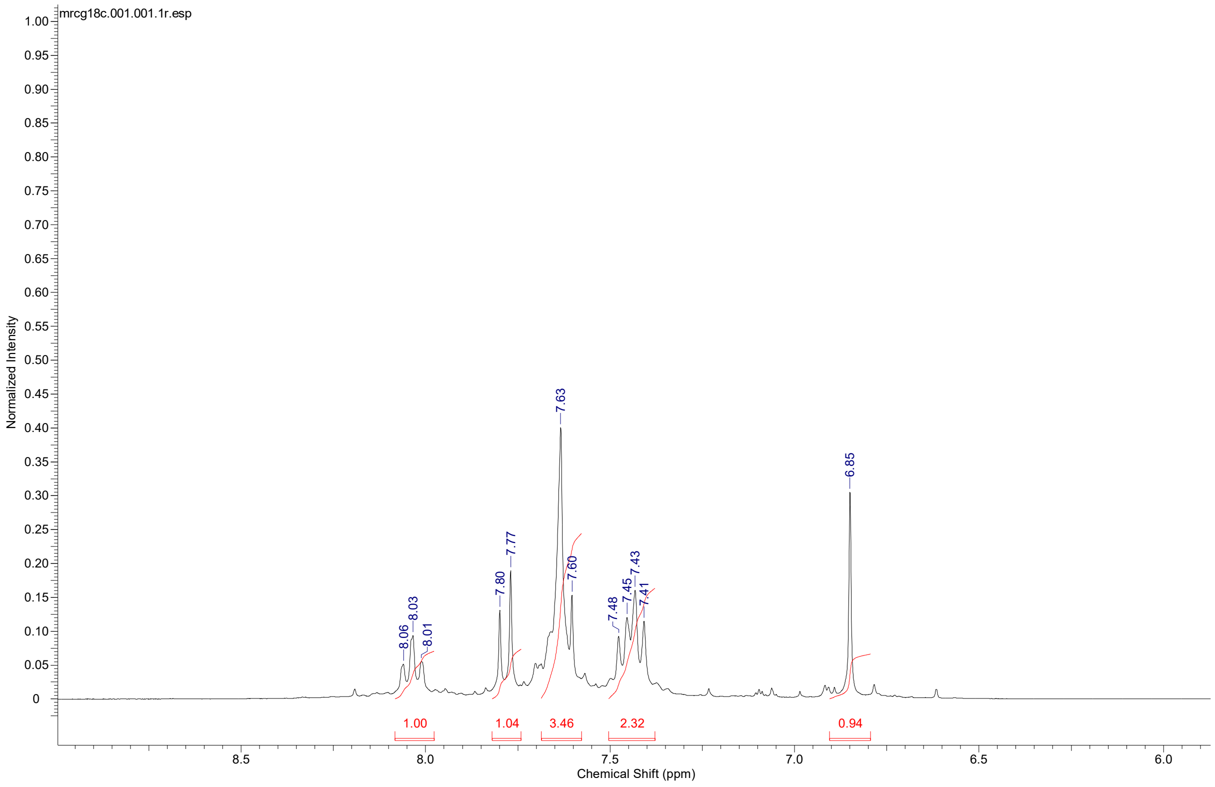

**7- MRCG19 2-amino-7-(3-fluorophenyl)-9H-chromeno[6,5-d]thiazol-9-one**

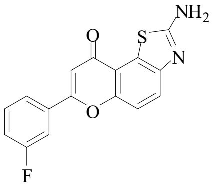


Mol. Wt.: 312; Anal. Calc. for $C_{16}H_9FN_2O_2S$: C, 61.53; H, 2.90; N, 8.97. Found: C, 61.49; H, 2.97; N, 8.84. m/z: 312.04 (100.0%).

$^1$H NMR (300 MHz, DMSO-$d_6$) δ ppm 7.22 (s, 1H, C-$H_8$), 7.46 (td, 1H, *J*=8.5, 2.3Hz, C-$H_{4'}$), 7.64 (td, 1H, *J*=8.0, 6.3Hz, C-$H_{5'}$), 7.68 (d, 1H, *J*=8.9Hz, C-$H_5$), 7.80 (d, 1H, *J*=8.9Hz, C-$H_4$),.7.99 (brd, 1H, *J*=8.0Hz, C-$H_{6'}$), 8.00 (dd, 1H, *J*=11.1, 1.8Hz, C-$H_{2'}$). $^{13}$C NMR (75 MHz, DMSO-$d_6$) δ ppm 106.64 (C-8), 113.26 (d, J=24.1Hz, $H_{2'}$), 115,84 (C-5), 117.83 (C-9a), 118.57 (d, J=21.9Hz, $H_{4'}$), 122.49 (C-6'), 123.10 (C-4), 124.34 (C-9b), 131.23 (d, J=8.3Hz, $H_{5'}$), 133.58 (d, J=8.3Hz, C-1'), 151.03 (C-5a), 151.03 (C-3a), 161.17 (C-7), 162.47 (d, J=247.00Hz, $H_{3'}$), 170.23 (C-2), 176.25 (C-9).

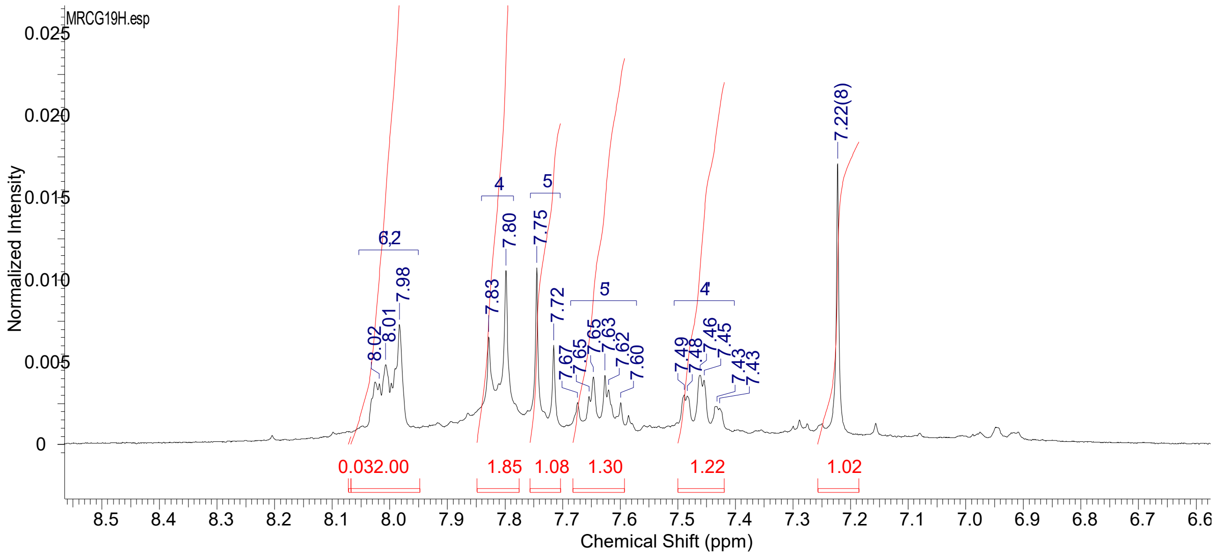


**8- MRCG05 2-amino-7-(4-fluorophenyl)-9H-chromeno[6,5-d]thiazol-9-one**

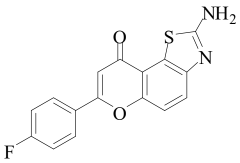


Mol. Wt.: 312; Anal. Calc. for $C_{16}H_9FN_2O_2S$: C, 61.53; H, 2.90; N, 8.97. Found: C, 61.47; H, 2.94; N, 8.87. m/z: 312.04 (100.0%),

$^1$H NMR (300 MHz, DMSO-$d_6$) δ ppm 7.12 (s, 1H, C-$H_8$), 7.44 (t, 2H, *J*=9.0Hz, C-$H_{3'}$), 7.60 (brs, 2H, $NH_2$), 7.68 (d, 1H, *J*=8.9Hz, C-$H_5$), 7.80 (d, 1H, *J*=8.9Hz, C-$H_4$), 8.21 (dd, 2H, *J*=9.0, 5.4Hz, C-$H_{2'}$). $^{13}$C NMR (75 MHz, DMSO-$d_6$) δ ppm 105.85 (C-8), 115,54 (C-5), 116.23 (d, J=22.6Hz, C-3'), 117.76 (C-9a), 123.35 (C-4), 124.93 (C-9b), 127.82 (C-1'), 129.05 (d, J=9.1Hz, C-2'), 150.80 (C-5a), 150.92 (C-3a), 161.68 (C-7), 163.41 (d, J=247,00Hz, C-4'), 170.14 (C-2), 176.19 (C-9).

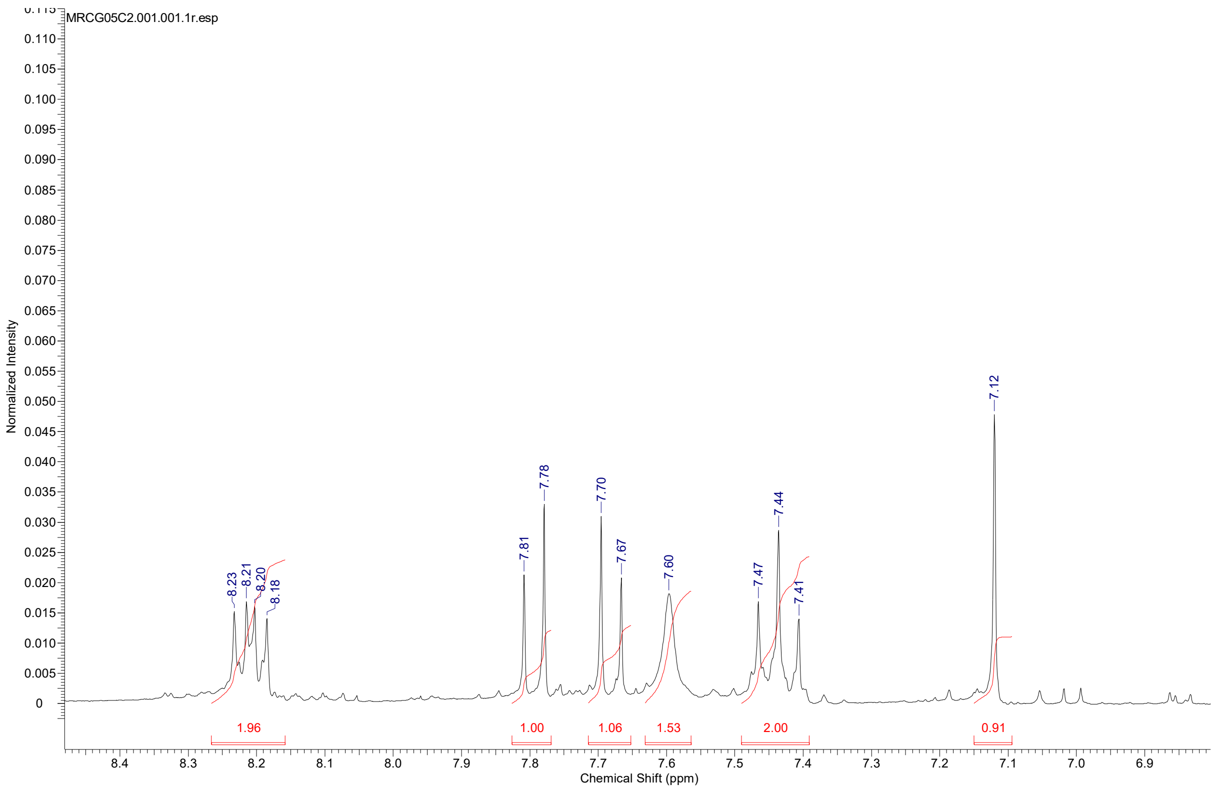


## 9- MRCG04 2-amino-7-(2-fluorophenyl)-9H-chromeno[6,5-d]thiazol-9-one

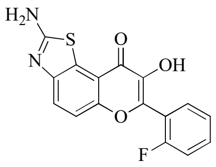


Mol. Wt.: 328; Anal. Calc. for $C_{16}H_9FN_2O_3S$: C, 58.53; H, 2.76; N, 8.53Found: C, 58.46; H, 2.86; N, 8.40. m/z: 328.03 (100.0%). $^1$H NMR (300 MHz, DMSO-$d_6$) δ ppm 7.40 (m, 2H, C-$H_{5',3'}$), 7.53 (d, 1H, $J$=8.9Hz, C-$H_5$), 7.59 (m, 3H, C-$H_{4'}$, $NH_2$), 7.77 (d, 1H, $J$=8.9Hz, C-$H_4$), 7.80 (dd, 1H, $J$=8.5 , 2.0Hz, C-$H_{6'}$), 9.51 (bs, 1H, OH). $^{13}$C NMR (75 MHz, DMSO-$d_6$) δ ppm 115.38 (C-5), 116.17 (d, J=22.6Hz, C-3'), 116.73 (C-9a), 119.06 (d, J=14.3Hz, C-1'), 123.46 (C-4), 124.42 (C-5'), 124.56 (C-9b), 131.25 (C-6'), 132.49 (d, J=9.0Hz, C-4'), 139.18 (C-8), 143.76 (C-7), 149.84 (C-5a), 150.63 (C-3a), 159.30 (d, J=251.00Hz, C-2'), 169.82 (C-2), 171.54 (C-9).

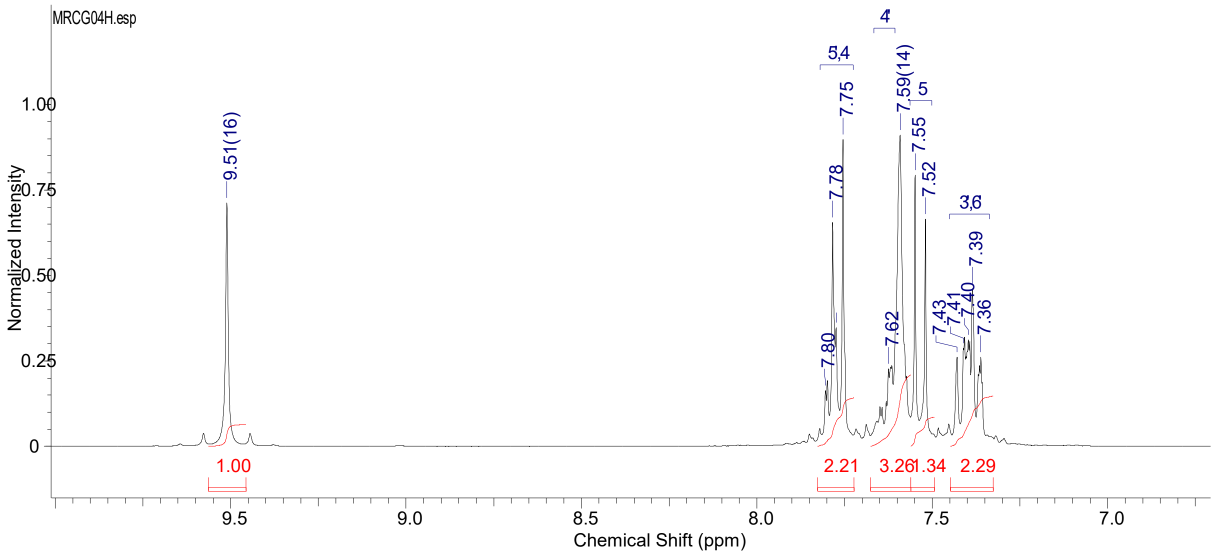

**10- MRCG21 2-amino-7-(3-fluorophenyl)-8-hydroxy-9H-chromeno[6,5-d]thiazol-9-one**

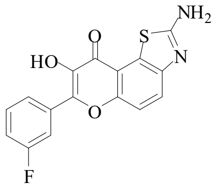


Mol. Wt.: 328; Anal. Calc. for $C_{16}H_9FN_2O_3S$: C, 58.53; H, 2.76; N, 8.53Found: C, 58.47; H, 2.89; N, 8.42. m/z: 328.03 (100.0%).

$^1$H NMR (300 MHz, DMSO-$d_6$) δ ppm 7.33-7.40 (dt, 1H, *J*=2.26, 8.50 Hz, C-$H_{4'}$), 7.61 (m, 3H, NH2, C-$H_{5'}$), 7.67-7.70 (d, 1H, *J*=8.87 Hz, C-$H_5$), 7.78-7.81 (d, 1H, *J*=8.88 Hz, C-$H_{4'}$), 8.03-8.07 (dd, 1H, *J*=2.45, 10.39 Hz, C-$H_{6'}$), 8.10-8.12 (d, 1H, *J*=8.12 Hz, C-$H_{2'}$), 10.02 (bs, 1H, OH). $^{13}$C NMR (300MHz, DMSO-$d_6$) δ ppm: 114.30 (d, J=24.0Hz, $H_{2'}$), 115.87 (C-5), 116.36 (C-9a), 116.85 (d, J=21.9Hz, C-4'), 123.55 (C-4), 123.84 (C-6'), 124.22 (C-9b), 130.83 (d, J=8.3Hz, C-5'), 133.76 (d, J=8.3Hz, C-1'), 139.53 (C-8), 144.08 (C-7), 149.07 (C-5a), 150.39 (C-3a), 162.5 (d, J=247Hz, C-3'), 169.96 (C-2), 172.01 (C-9).

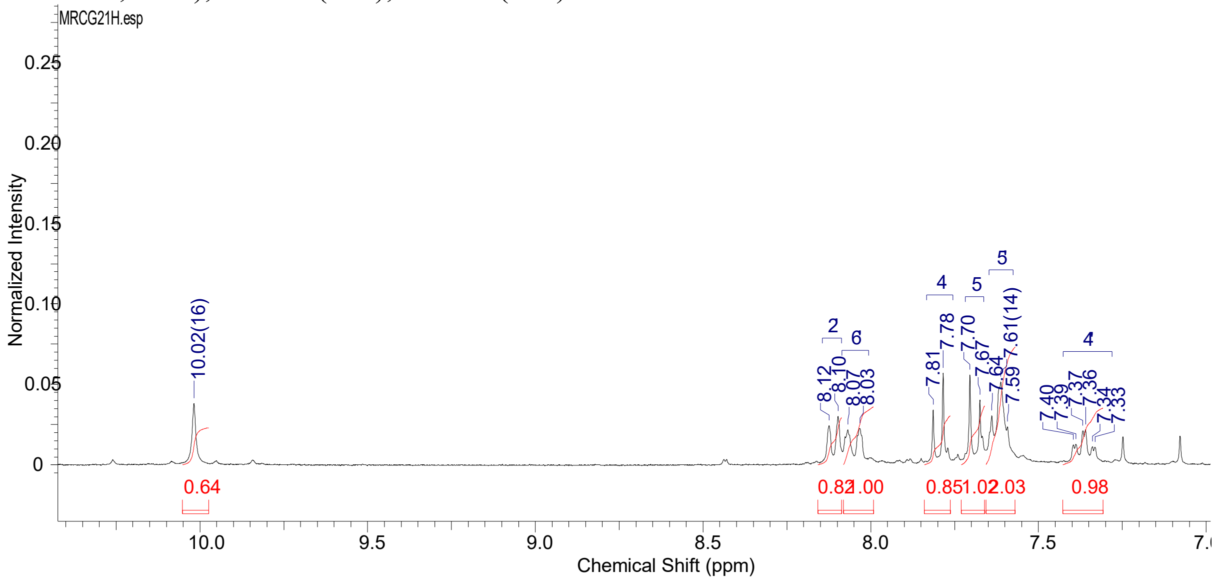


**11- MRCG06 2-amino-7-(4-fluorophenyl)-8-hydroxy-9H-chromeno[6,5-d]thiazol-9-one**

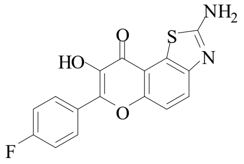


Mol. Wt.: 328; Anal. Calc. for $C_{16}H_9FN_2O_3S$: C, 58.53; H, 2.76; N, 8.53. Found: C, 58.47; H, 2.84; N, 8.42. m/z: 328.03 (100.0%).

$^1$H NMR (300 MHz, DMSO-$d_6$) δ ppm 7.42 (t, 2H, *J*=9.0Hz, C-$H_{3'}$), 7.74 (d, 1H, *J*=8.9Hz, C-$H_5$), 7.85 (d, 1H, *J*=8.9Hz, C-$H_4$), 8.29 (dd, 2H, *J*=9.0, 5.4Hz, C-$H_{2'}$). $^{13}$C NMR (75 MHz, DMSO-$d_6$) δ ppm 116.77 (C-5), 115.96 (d, J=21.9Hz, C-3'), 116.23 (C-9a), 122.16 (C-4), 122.56 (C-9b), 128.01 (C-1'), 130.55 (d, J=8.3Hz, C-2'), 139.00 (C-8), 145.63 (C-7), 150.80 (C-5a), 150.80 (C-3a), 163.00 (d, J=247,00Hz, C-4'), 170.29 (C-2), 172.00 (C-9).

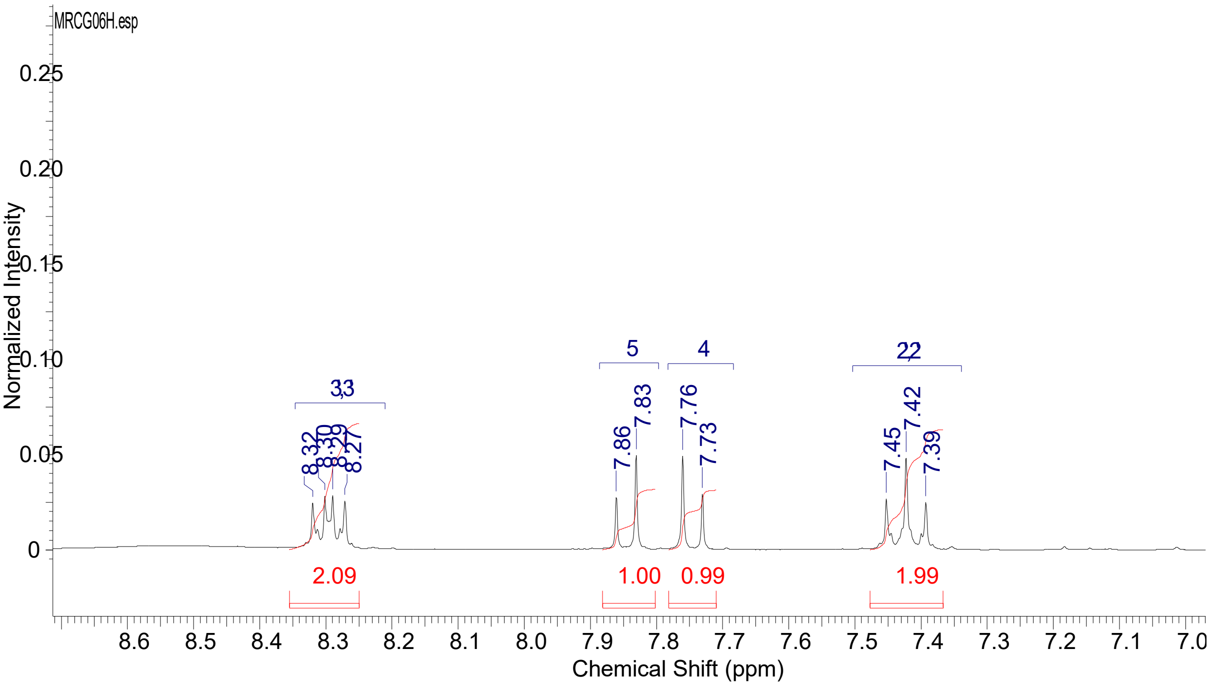


**12 -MR412 2-amino-8-hydroxy-7-phenyl-9H-chromeno[6,5-d]thiazol-9-one**

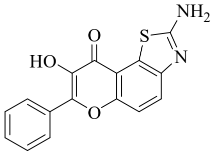


Mol. Wt.: 310 ; Anal. Calc. for $C_{16}H_{10}N_2O_3S$: C, 61.93; H, 3.25; N, 9.03. Found: C, 61.82; H, 3.35; N, 9.01.

$^1$H NMR (300 MHz, DMSO-$d_6$) δ ppm: 7.52 (m, 1H, $H_{4'}$), 7.57 (m, 2H, $H_{3'}$), 7.63 (brs, 2H, $NH_2$), 7.64 (d, 1H, J=8.9Hz, $H_5$), 7.78 (d, 1H, J=8.9Hz, $H_4$), 8.13 (m, 2H, $H_{2'}$), 8.17 (s, 1H, OH). $^{13}$C NMR (75 MHz, DMSO-$d_6$) δ ppm: 115.44 (C-5), 116.24 (C-9a), 123.44 (C-4), 124.41 (C-9b), 127.67 (C-2’), 128.55 (C-3’), 129.86 (C-1’), 131.41 (C-4’), 138.83 (C-8), 145.50 (C-7), 149.77 (C-5a), 150.17 (C-3a), 169.72 (C-2), 171.75 (C-9).

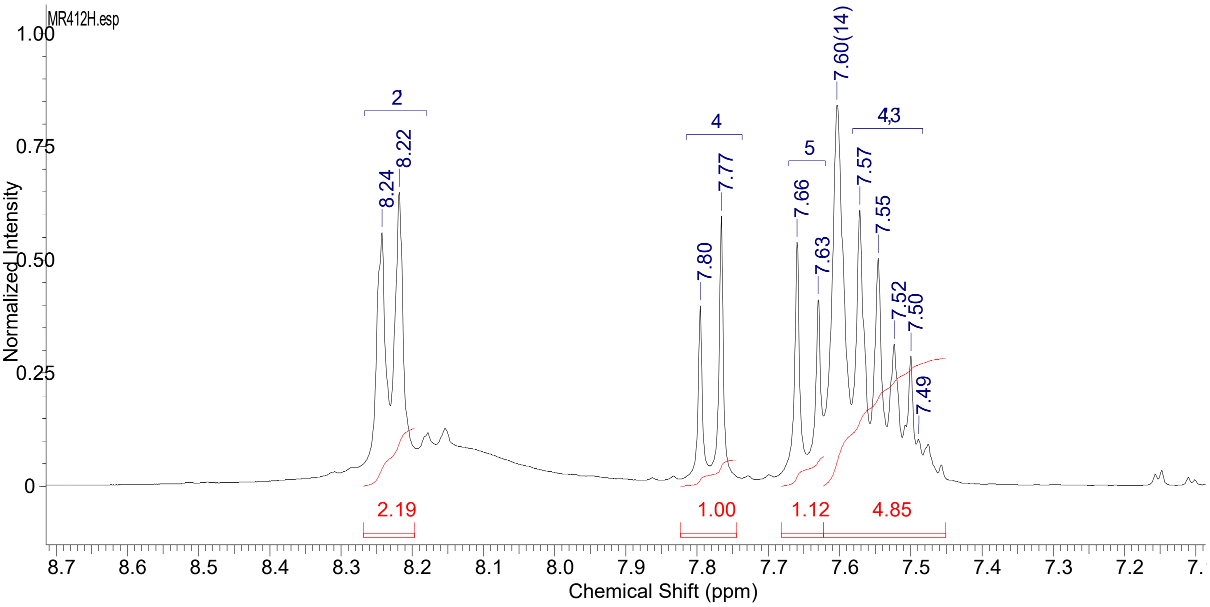

**13- MRCG28 2-amino-7-(2-fluorophenyl)-8-methoxy-9H-chromeno[6,5-d]thiazol-9-one**

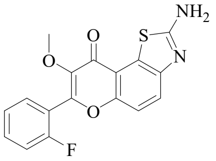


Mol. Wt.: 342; Anal. Calc. for $C_{17}H_{11}FN_2O_3S$: C, 59.64; H, 3.24; N, 8.18. Found: C, 59.59; H, 3.36; N, 8.14. m/z: 342,05 (100,0%).

$^1$H NMR (300 MHz, DMSO-$d_6$) δ ppm 3.81 (s,3H, $OCH_3$), 7.38-7.42 (brd, 1H, *J*=8.5 Hz, C-$H_{5'}$), 7.43-7.47 (brd, 1H, *J*=8.5 Hz, C-$H_{3'}$), 7.53-7.56 (d, 1H, *J*=8.6 Hz, C-$H_4$), 7.63 (bs, 2H, $NH_2$), 7.65 (brd, 1H, C-$H_{4'}$), 7.74 (t, 1H, *J*=7.5 Hz, C-$H_{6'}$), 7.76-7.79 (d, 1H, *J*=8.6 Hz, C-$H_5$).

$^{13}$C NMR (75 MHz, DMSO-$d_6$) δ ppm 60.23 ( $OCH_3$), 115.61 (C-5), 116.33 ( d, J=20.1Hz, C-3'), 118.40 (C-9a), 118.80 ( d, *J*=14.3Hz, C-1'), 123.71 (C-4), 124.54 (C-9b), 124.90 (C-5'), 131.35 (C-6'), 133.20 ( d, J=8.6Hz, C-4'), 141.19 (C-7), 150.37 (C-5a), 150.78 (C-3a), 152.24 (C-8), 159.20 ( d, J=250.00Hz, C-2'), 170.34 (C-2), 172.71 (C-9).

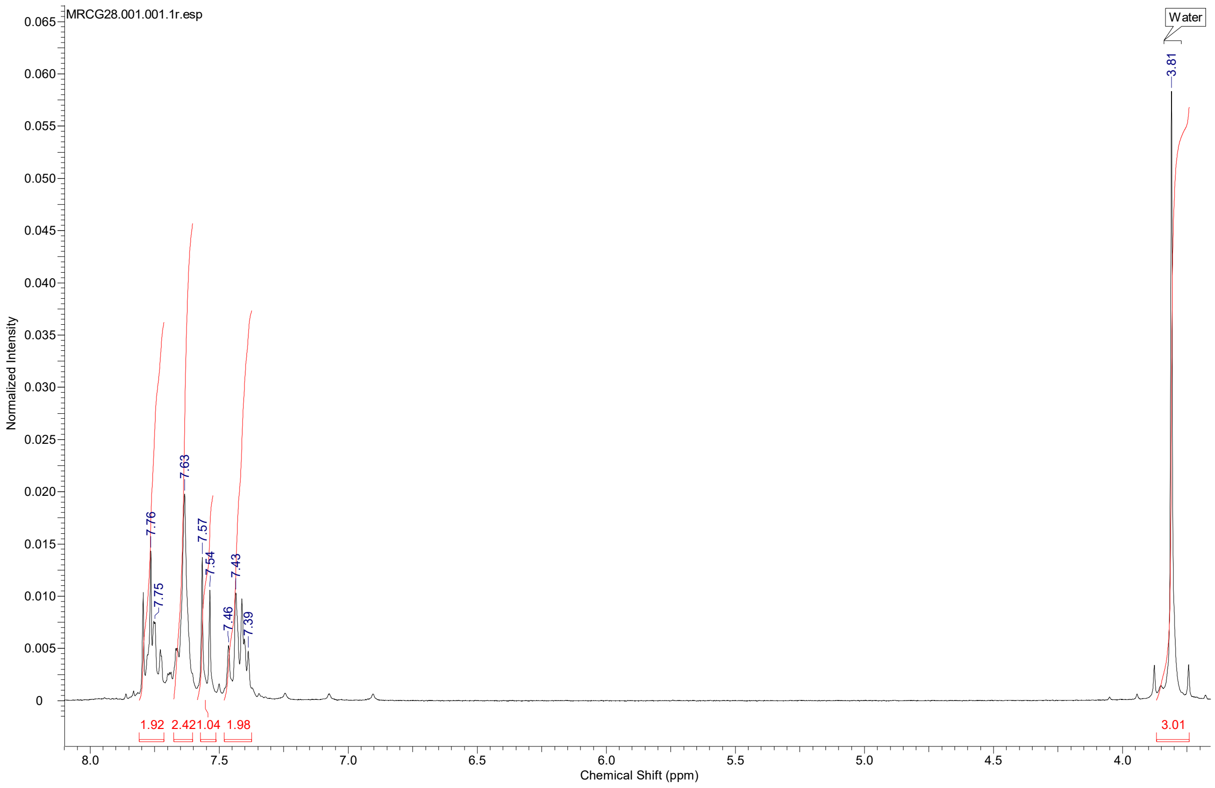


**14- MRCG31 2-amino-7-(3-fluorophenyl)-8-methoxy-9H-chromeno[6,5-d]thiazol-9-one**

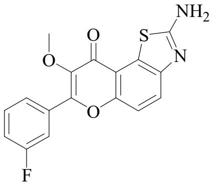


Mol. Wt.: 342; Anal. Calc. for $C_{17}H_{11}N_2FO_3S$: C, 59.64; H, 3.24; N, 8.18. Found: C, 59.32; H, 3.31; N, 8.08. m/z: 342,05 (100,0%).

$^1$H NMR (300 MHz, DMSO-$d_6$) δ ppm 3.87 (s, 3H, $OCH_3$), 7.43 (td, 1H, *J*=8.5, 2.3Hz, C-$H_{4'}$), 7.61 (td, 1H, *J*=8.0, 6.3Hz, C-$H_{5'}$), 7.62 (brs, 2H, $NH_2$), 7.68 (d, 1H, *J*=8.9Hz, C-$H_5$), 7.80 (d, 1H, *J*=8.9Hz, C-$H_4$), 7.86 (dd, 1H, 11.1, 1.8Hz, C-$H_{2'}$), 7.92 (brd, 1H, *J*=8.0Hz, C-$H_{6'}$).

$^{13}$C NMR (75 MHz, DMSO-$d_6$) δ ppm 60.03 ($OCH_3$), 115.41 (d, J=24.1Hz, $H_{2'}$), 115,69 (C-5), 118.41 (C-9a), 117.88 (d, J=21.1Hz, $H_{4'}$), 124.64 (C-6'), 123.78 (C-4), 124.80 (C-9b), 131.04 (d, J=8.3Hz, $H_{5'}$), 132.85 (d, J=9.0Hz, C-1'), 140.96 (C-7), 150.33 (C-5a), 150.50 (C-3a), 153.36 (C-8), 162.15 (d, J=243.00Hz, $H_{3'}$), 170.24 (C-2), 173.07 (C-9).

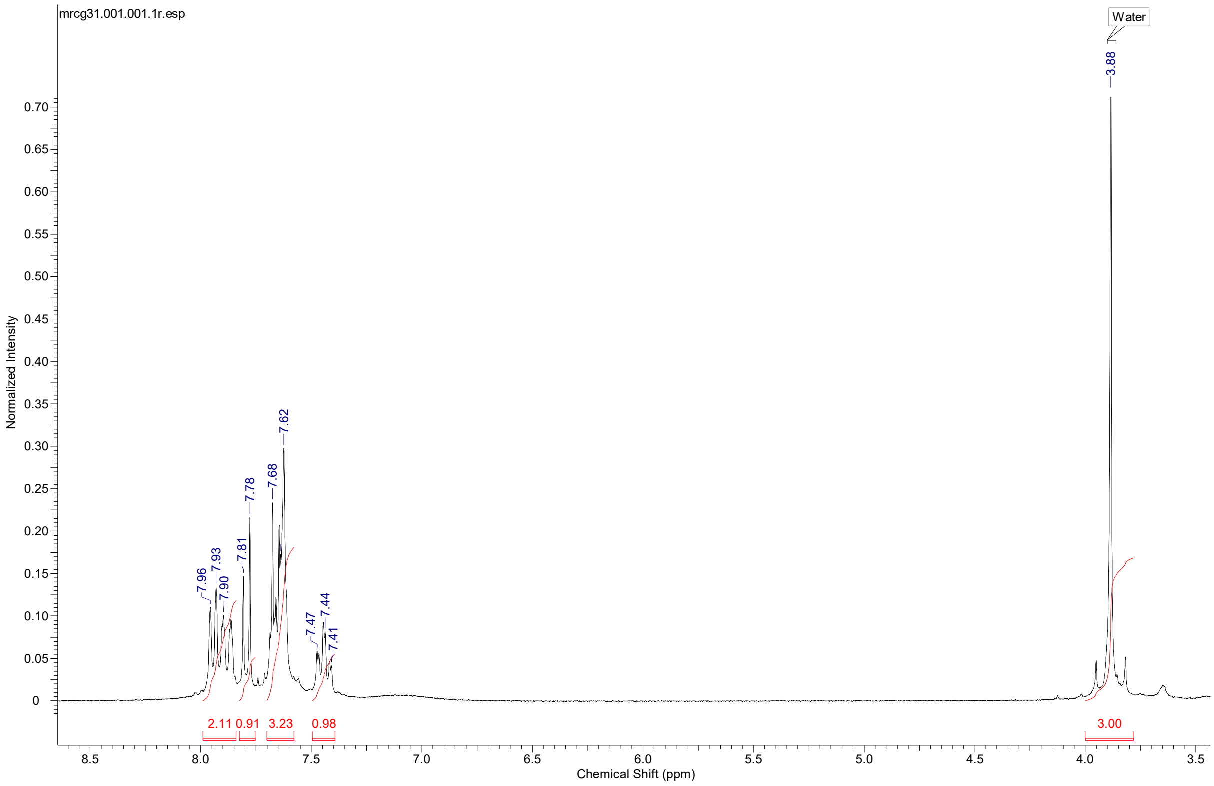


**15- MRCG09 2-amino-7-(4-fluorophenyl)-8-methoxy-9H-chromeno[6,5-d]thiazol-9-one**

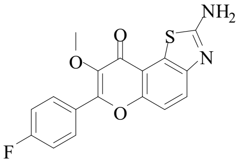


Mol. Wt.: 342; Anal. Calc. for $C_{17}H_{11}FN_2O_3S$: C, 59.64; H, 3.24; N, 8.18. Found: C, 59.56; H, 3.31; N, 8.04. m/z: 342.05 (100.0%).
$^1$H NMR (300 MHz, DMSO-$d_6$) δ ppm 3.85 ($OCH_3$), 7.46 (t, 2H, $J$=9.0Hz, C-$H_{3'}$), 7.75 (d, 1H, $J$=8.9Hz, C-$H_5$), 7.85 (d, 1H, $J$=8.9Hz, C-$H_4$), 8.16 (dd, 2H, $J$=9.0, 5.4Hz, C-$H_{2'}$). $^{13}$C NMR (75 MHz, DMSO-$d_6$) δ ppm 59.84 ($OCH_3$), 116.19 (C-5), 116.38 (d, J=21.9Hz, C-3’), 118.23 (C-9a), 122.50 (C-4), 122.50 (C-9b), 126.95 (C-1’), 131.00 (d, J=9.8Hz, C-2’), 140.37 (C-7), 150.46 (C-5a), 150.46 (C-3a), 154.37 (C-8),163.05 (d, J=247,00Hz, C-4’), 170.25 (C-2), 172.86 (C-9).

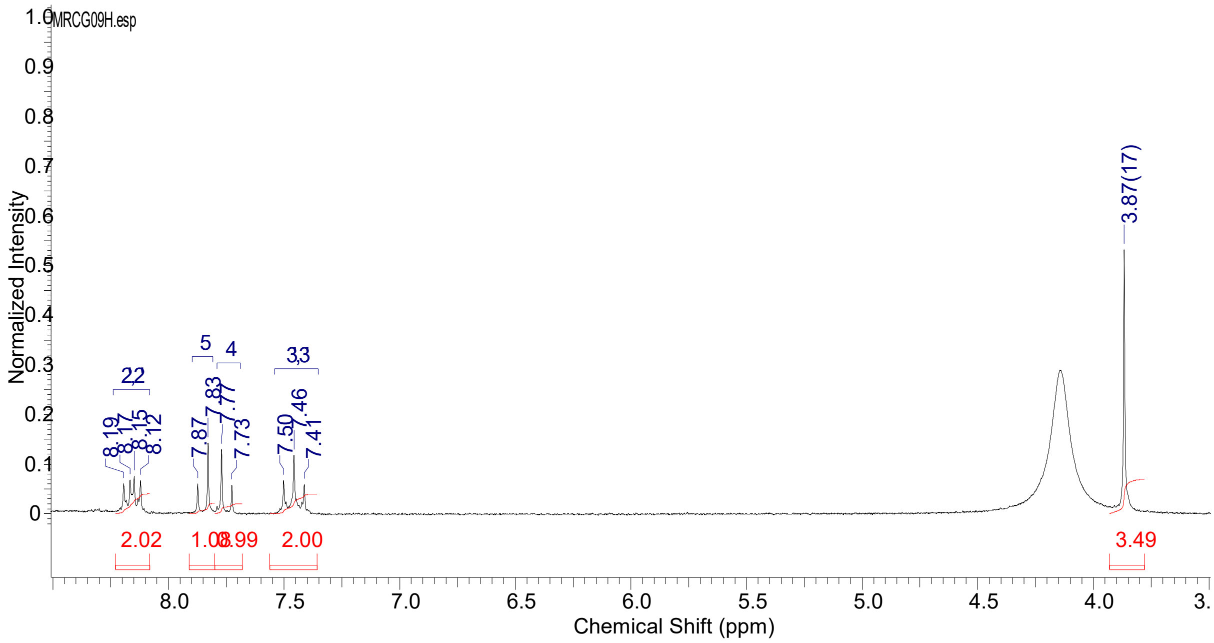

## Quinazoline Synthetic protocole

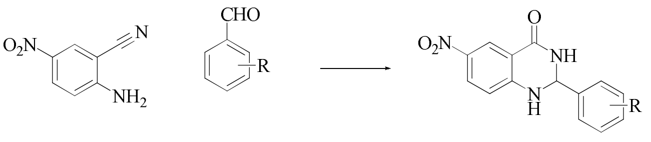

Following the procedure described by Tang et al. [84] various nitro-1,2-dihydroquinazolin-4(3H)-one could be obtained.

5-nitroanthranilonitrile (2.5 mmol) and benzaldehyde (2.5 mmol) were added to a solution of DMF (10 mL) and $ZnCl_2$ (3 mmol). The mixture was heated at reflux for 1.5 h. The cooled reaction mixture was quenched with water and the precipitate was collected by sucion.

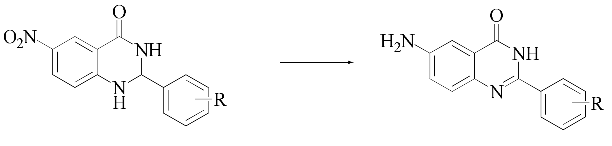

Starting with nitro derivatives (2 mmol) in EtOH 95% (15 mL) and Pd/C (0.2 mol%). The solution was placed under $H_2$ and stirred until no more beginning product appeared as indicated by TLC. The solution was filtered off to remove Pd/C and the organic layer was removed under reduce pressure. The resulting solid was poured onto 1N HCl solution to give a red precipitate of pure amino derivatives.

**Following the general procedure for preparation of 2-aminothiazole moiety with various amino compounds (vi).**

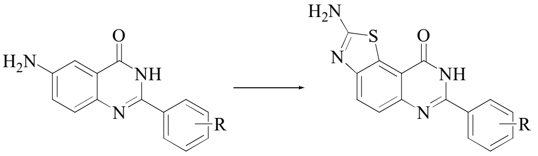

### 16- MRDS10150 2-amino-7-(3-phenoxyphenyl)thiazolo[5,4-f]quinazolin-9(8H)-one

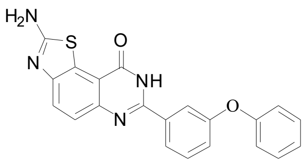

Mol. Wt.: 386; Anal. Calc. for $C_{21}H_{14}N_4O_2S$: C, 65.27; H, 3.65; N, 14.50. Found: C, 65.25; H, 3.67; N, 14.42.$^1$H NMR (75MHz, DMSO-*d6*) δ ppm: 7.08 (m, 2H, $H_{2''}$), 7.18 (brd, 1H, J= 7.9 Hz, $H_{4'}$), 7.19 (m, 1H, $H_{4''}$), 7.42 (m, 2H, $H_{3''}$), 7.57 (t, 1H, J= 7.9 Hz, $H_{5'}$), 7.62 (d, 1H, J= 8.5 Hz, $H_5$), 7.80 (d, 1H, J= 8.5 Hz, $H_4$), 7.85 (brs, 1H, $H_{2'}$), 7.96 (brd, 1H, J= 7.9 Hz, $H_{6'}$), 12.66 (brs, 1H, NH). $^{13}$C NMR (75MHz, DMSO-*d6)* δ ppm: 161.49(9), 149.38(7), 125.16(5), 123.81(4), 150.16(3a), 125.59(9b), 115.24(9a), 143.59(5a), 170.29(2), 134.80(1'), 117.80(2'), 156.92(3'), 121.40(4'), 130.41(5'), 122.76(6'), 156.51(1"), 118.87(2"), 130.22(3"), 123.81(4").

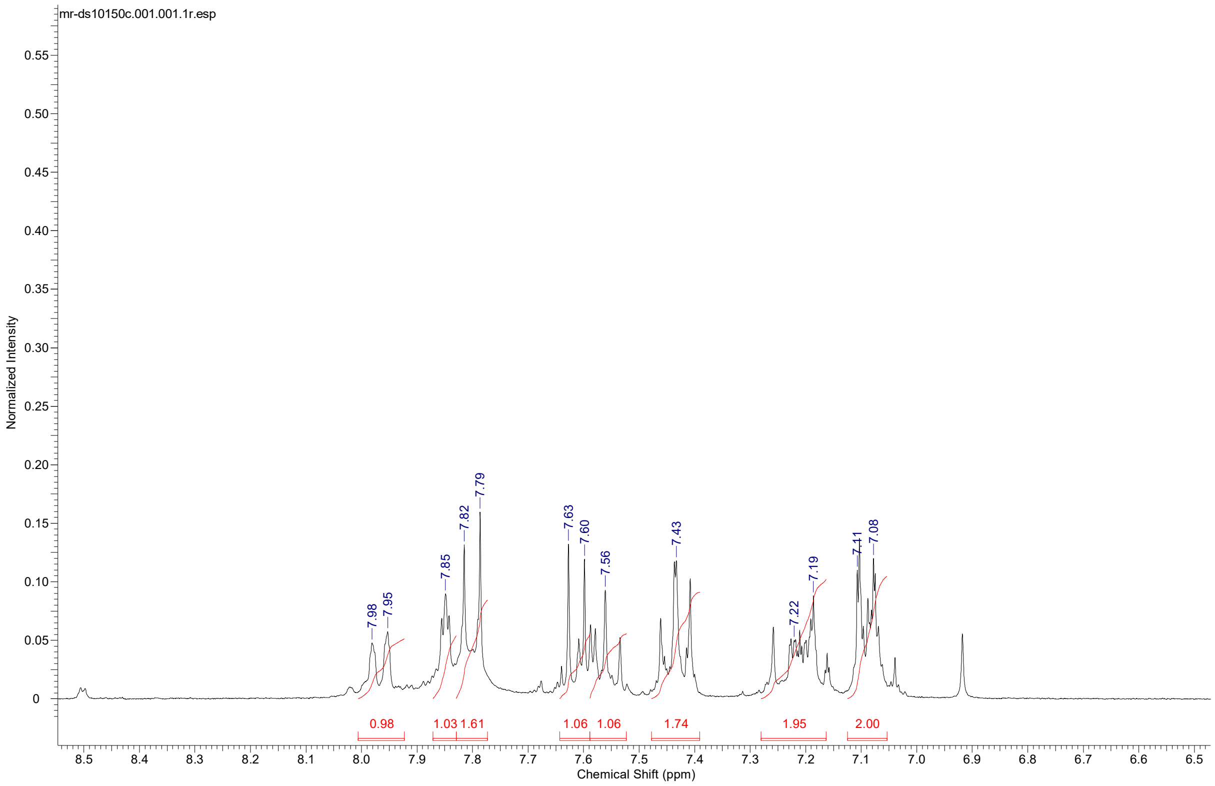


**17- MRDS10106B 2-amino-7-(4-phenoxyphenyl)thiazolo[5,4-f]quinazolin-9(8H)-one**

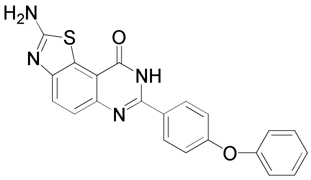


Mol. Wt.: 386; Anal. Calc. for $C_{21}H_{14}N_4O_2S$: C, 65.27; H, 3.65; N, 14.50. Found: C, 65.21; H, 3.69; N, 14.42. $^1$H NMR (75MHz, DMSO-*d$_6$*) δ ppm: 7.07 (d, 2H, $H_{3'}$), 7.08 (m, 2H, $H_{2''}$), 7.19 (m, 2H, $H_{4''}$), 7.42(m, 2H $H_{3''}$), 7.56 (d, 1H, $H_5$), 7.58 (brs, 2H, $NH_2$), 7.75 (d, 1H, $H_6$), 8.17 (d, 2H, $H_{2'}$), 12.55 (brs, 1H, NH). $^{13}$C NMR (75MHz, DMSO-*d$_6$*) δ ppm: 115.07(9), 117.87(2''), 119.81(3'), 124.46(6), 124.59(4''), 124.93(5), 126.43(8), 127.74(1'), 129.82(3''), 130.48(2'), 143.58(10), 149.35(3), 151.87(7), 155.73(1''), 159.67(4'), 161.66(1).

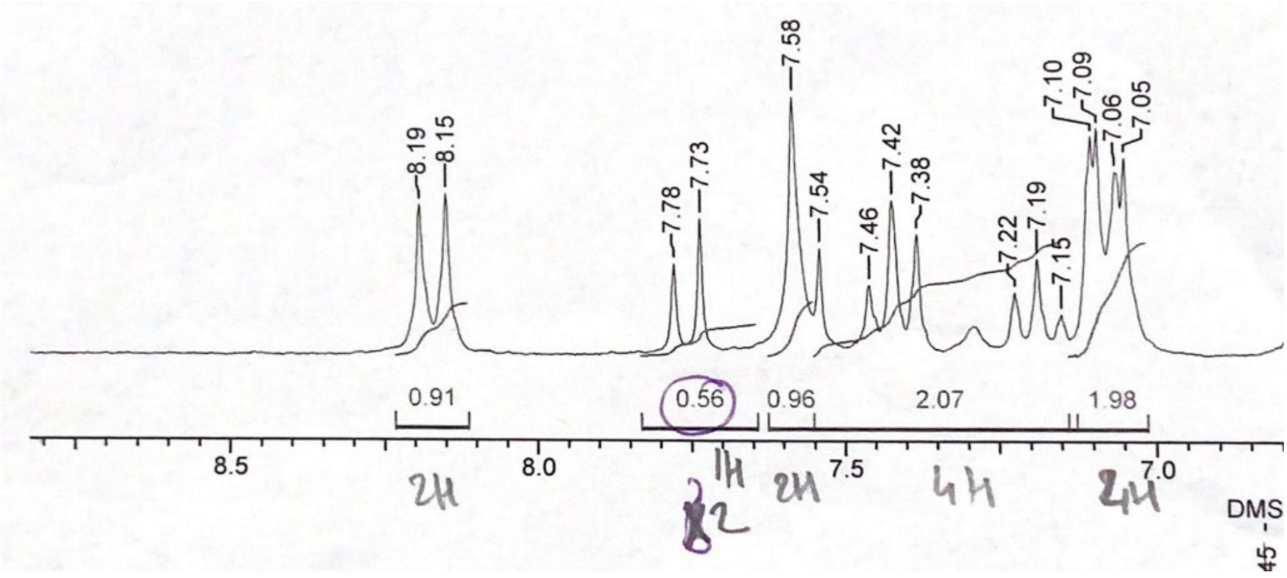

Supplement: Supplementary file 1 [file ijms-24-15050-s001.zip › Supplementary Materials File S1.pdf]
