# Supplementary material for: 2-Aminothiazole-Flavonoid Hybrid Derivatives Binding to Tau Protein and Responsible for Antitumor Activity in Glioblastoma
Source: Int J Mol Sci. 2023 Oct 10;24(20):15050. doi: 10.3390/ijms242015050 (PMC10606064; doi:10.3390/ijms242015050)
Supplement: Supplementary file 1 [file ijms-24-15050-s001.zip › Supplementary Materials File S2.pdf]

Supplementary Materials File S2 — Supplementary tables and figures

Thiazole-flavonoid hybrid derivatives binding to Tau protein and responsible for antitumor activity in glioblastoma

Rayane Hedna [1], Attilio DiMaio [2], Maxime Robin [2], Diane Allegro [1], Mario Tatoni [1], Vincent Peyrot [1], Pascale Barbier [1], Hervé Kovacic [1], Gilles Breuzard [1,*]

1 - Faculté des Sciences Médicales et Paramédicales, Institut de Neurophysiopathologie (INP), UMR 7051, CNRS, Aix Marseille Université, 13005 Marseille, France

2 - Faculté de Pharmacie, Institut Méditerranéen de Biodiversité et Ecologie marine et continentale (IMBE), UMR 7263, CNRS, IRD 237, Aix-Marseille Université, 13005 Marseille, France

* Correspondence: gilles.breuzard@univ-amu.fr

Supplementary Tables

Table S1. The anti-metabolic activity of compound 9 is recovered after the rescued expression of Tau protein in U87 shTau cells.

compounds	IC_{50} [(a)]
U87 shCTRL + eGFP	1.8 ± 0.2
U87 shTau + EGFP	> 100
U87 shTau + EGFP-Tau	4.3 ± 1.2

(a) Drug concentration that inhibits metabolic activity by 50 % after cell incubation in culture media for 72 h. Data are the mean ± SEM of three independent experiments.

Supplementary figures

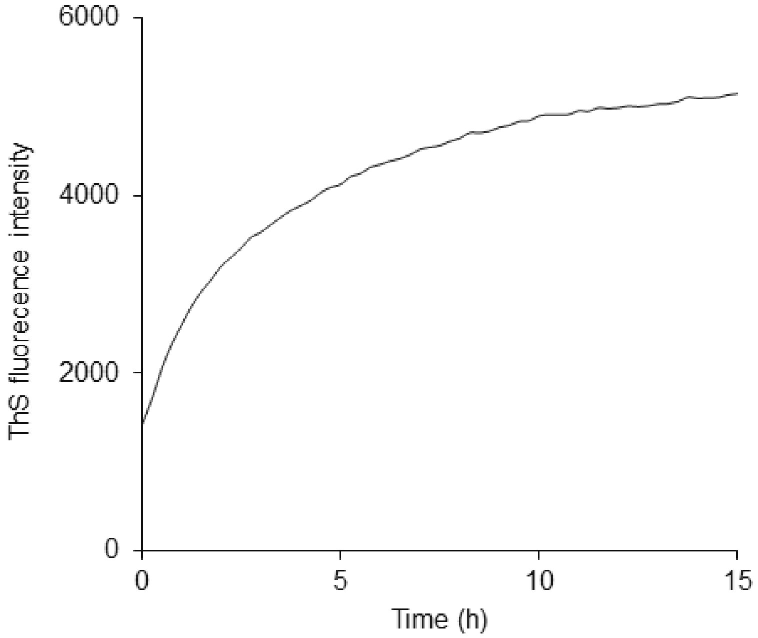

Supplementary Figure S1. Time-course of heparin-induced Tau aggregation monitored by ThS fluorescence assay. The formation of Tau aggregates increases and reaches a steady state after 8 hours of monitoring.

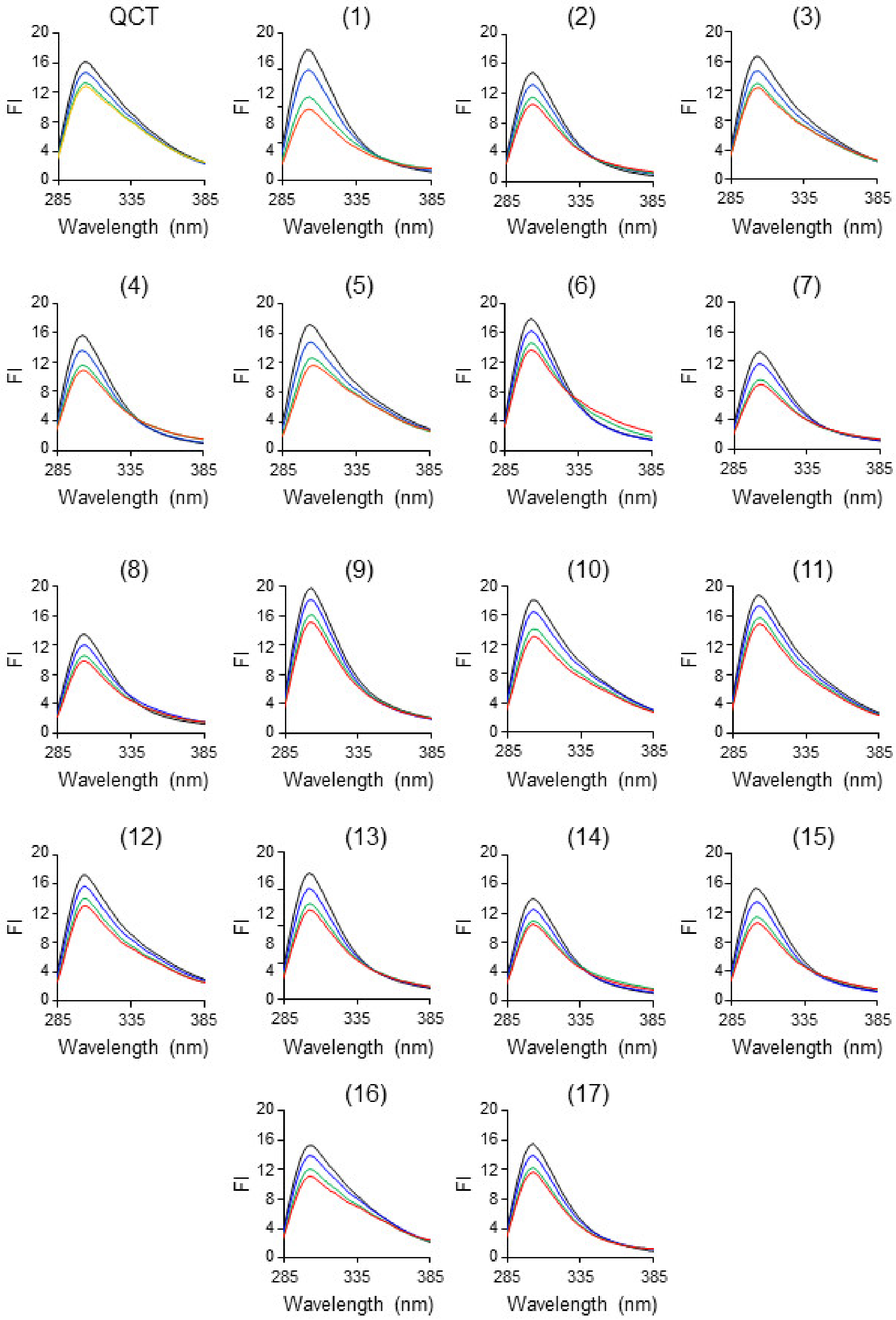

Supplementary Figure S2. Fluorescence changes in the Tau protein emission spectra produced by the binding of QCT and compounds (1-17) (excitation at 275 nm): Tau 5 µM (black curves) in buffer B (20 mM NaPi, 1 mM TCEP, 5% DMSO, pH = 6.8) at 25°C; Tau at same concentration with 4 µM (blue curves), 14 µM (green curves), 20 µM (red curves) of compounds.

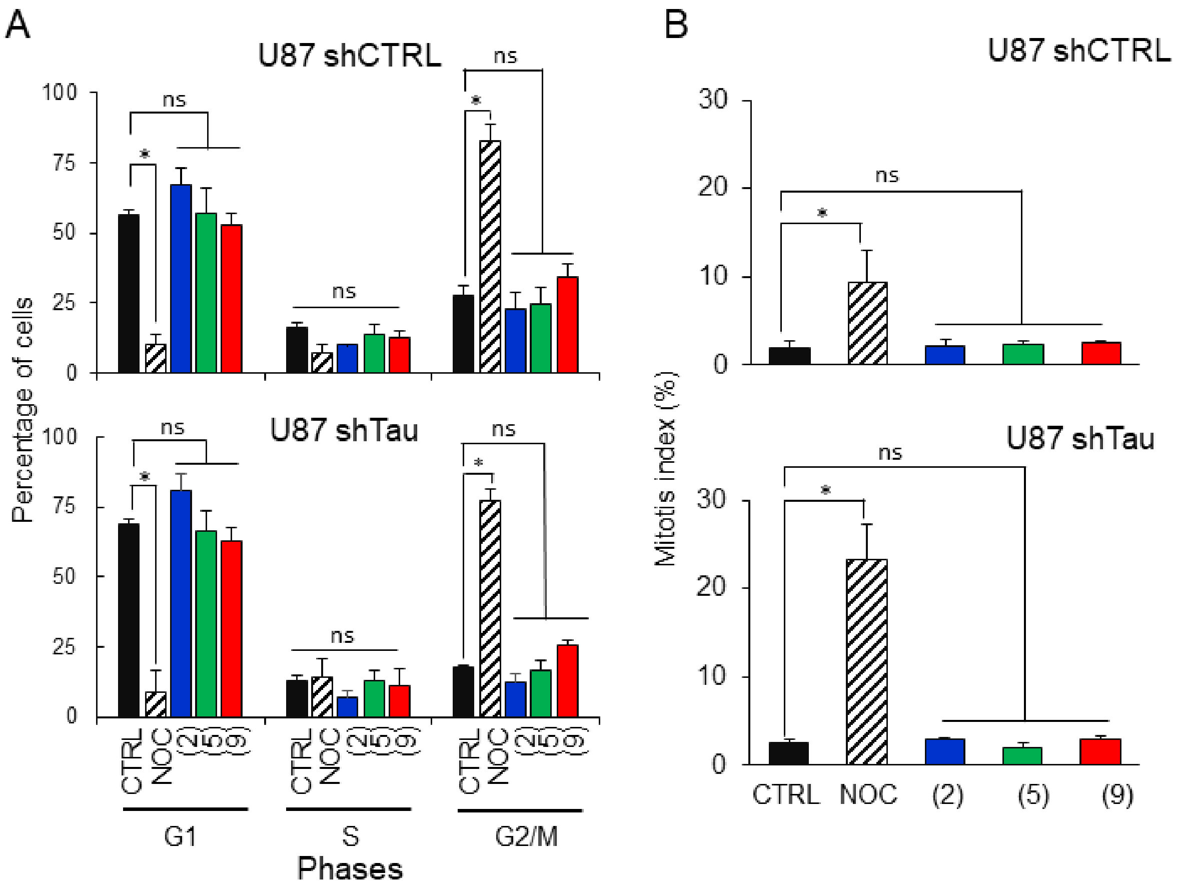

Supplementary Figure S3. Compounds did not disturb the cell cycle progression. (A) No change of phases distribution in U87 shCTRL (upper panel) and shTau (lower panel) cells treated with 1 µM nocodazole (NOC) or 10 µM compounds (2), (5) and (9) for 48 hours; cells were analyzed with the FACScan flow cytometer; all reported values (mean SD of three independent experiments) correspond to percentages of cells in G1, S and G2/M phases; (B) No significant variation of mitosis index for compound-treated cells: DAPI-labelled nuclei of cells were analyzed using HCS-based imaging approach to determine ratios (in %) of cells in mitosis on total cells. Significant statistical differences were calculated using a non-parametric Mann-Whitney test with **P < 0.01.

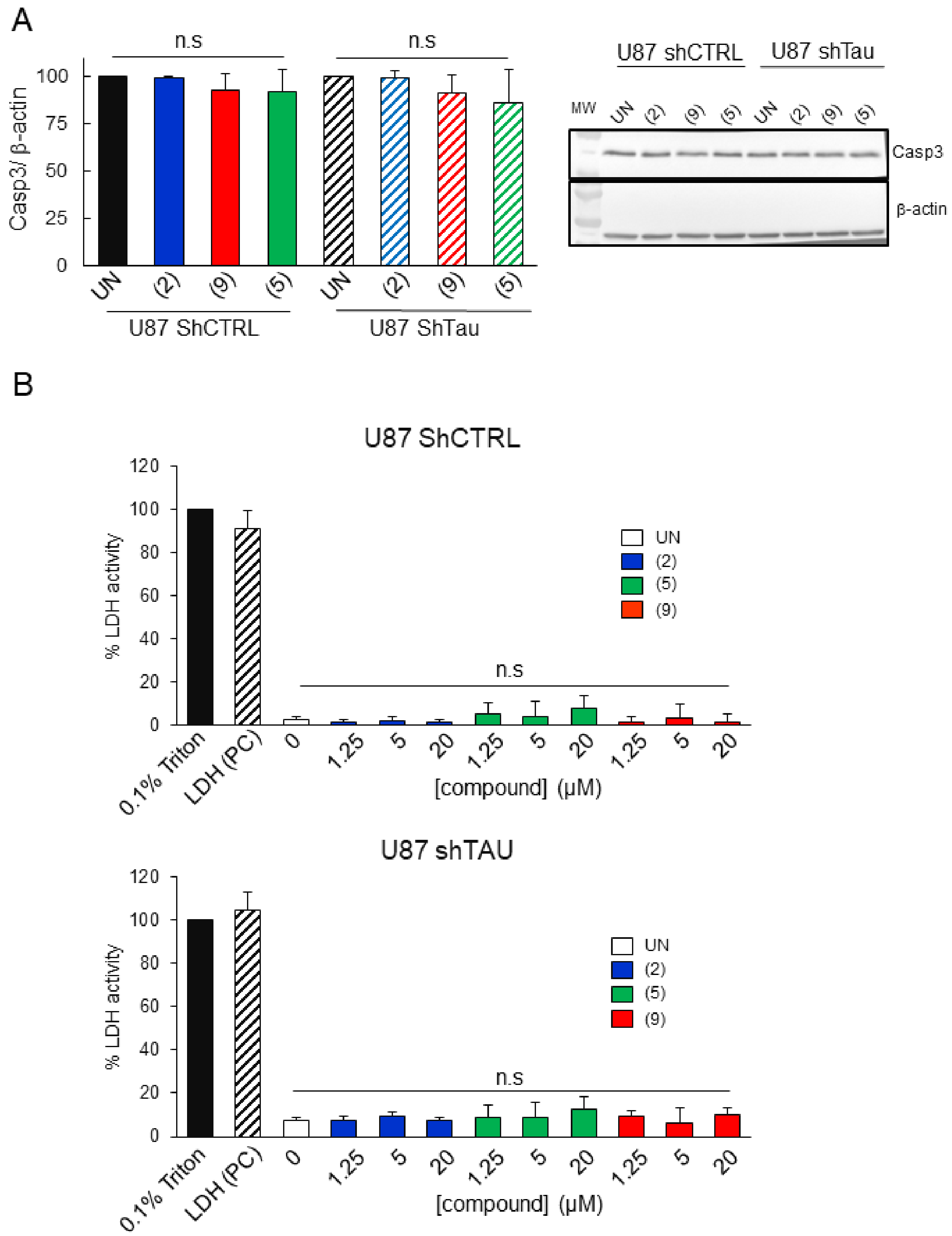

Supplementary Figure S4. Compounds (2) and (9) did not promote apoptosis or necrosis cell death. (A) The apoptotic protein caspase 3 (Casp 3) was analyzed by western blot of U87 shCTRL and U87 shTau cells: for each sample the ratio between the amount of Casp3 and GAPDH was calculated as relative fold of the reference value (with or without the compound); n = 3 lysates, ns: non-significant. The inset in (A) shows representative examples of western blot used for quantification. (B) The activity of necrotic LDH protein was analyzed by fluorescence emission in U87 shCTRL (upper panel) and U87 shTau (lower panel) cells. For each sample, the ratio between LDH florescence was calculated as the percentage of activity against reference value (0,1% Triton); n = 3 lysates; Significant statistical differences were calculated using a non-parametric Mann-Whitney test with **P < 0.01; n.s: non-significant.

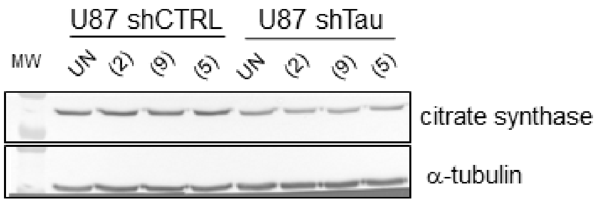

Supplementary Figure S5. Effects of Compounds (2) and (9) on cellular mitochondrial abundance. Western blot analysis of the mitochondrial matrix enzyme citrate synthase in U87 shCTRL and U87 shTau cells after 24-hour treatment with 5 μM of the compounds. Alpha-tubulin was used as a housekeeping protein. The results demonstrate that compounds (2) and (9) do not significantly alter cellular mitochondrial abundance compared to the non-treated condition in both cell lines.

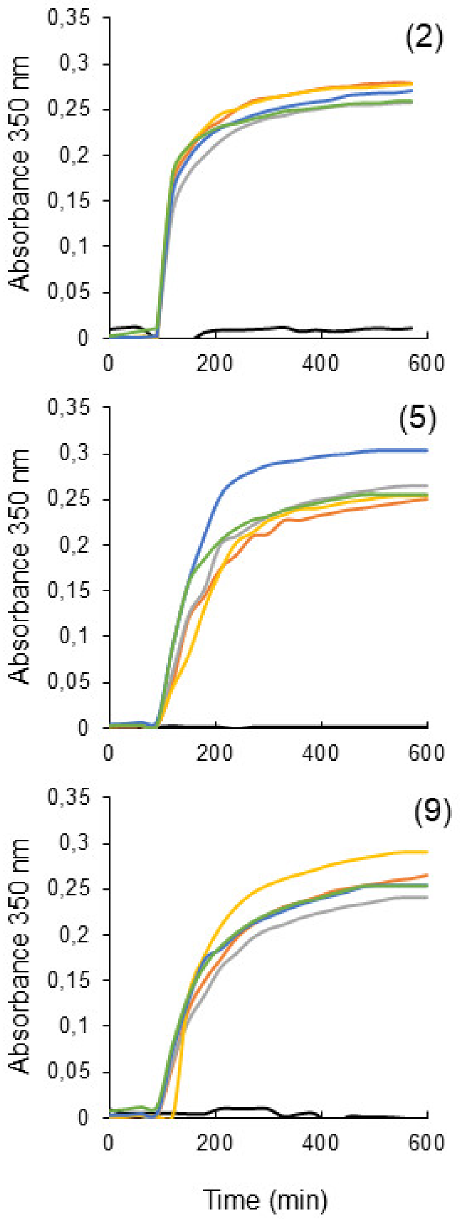

Supplementary Figure S6. Compounds (2-9) had no effect on the turbidity time course of in vitro Tau-induced microtubule assembly. The reaction was started by warming the solution at 37°C. Panels A-C show tubulin at 10 µM (black curves) and aliquots of the same solution with 5 µM Tau without compound (in gray) or with 2.5 µM (blue), 5 µM (green), 10 µM (yellow), 50 µM (red) compound (2) (upper), compound (5) (middle) and compound (9) (lower).

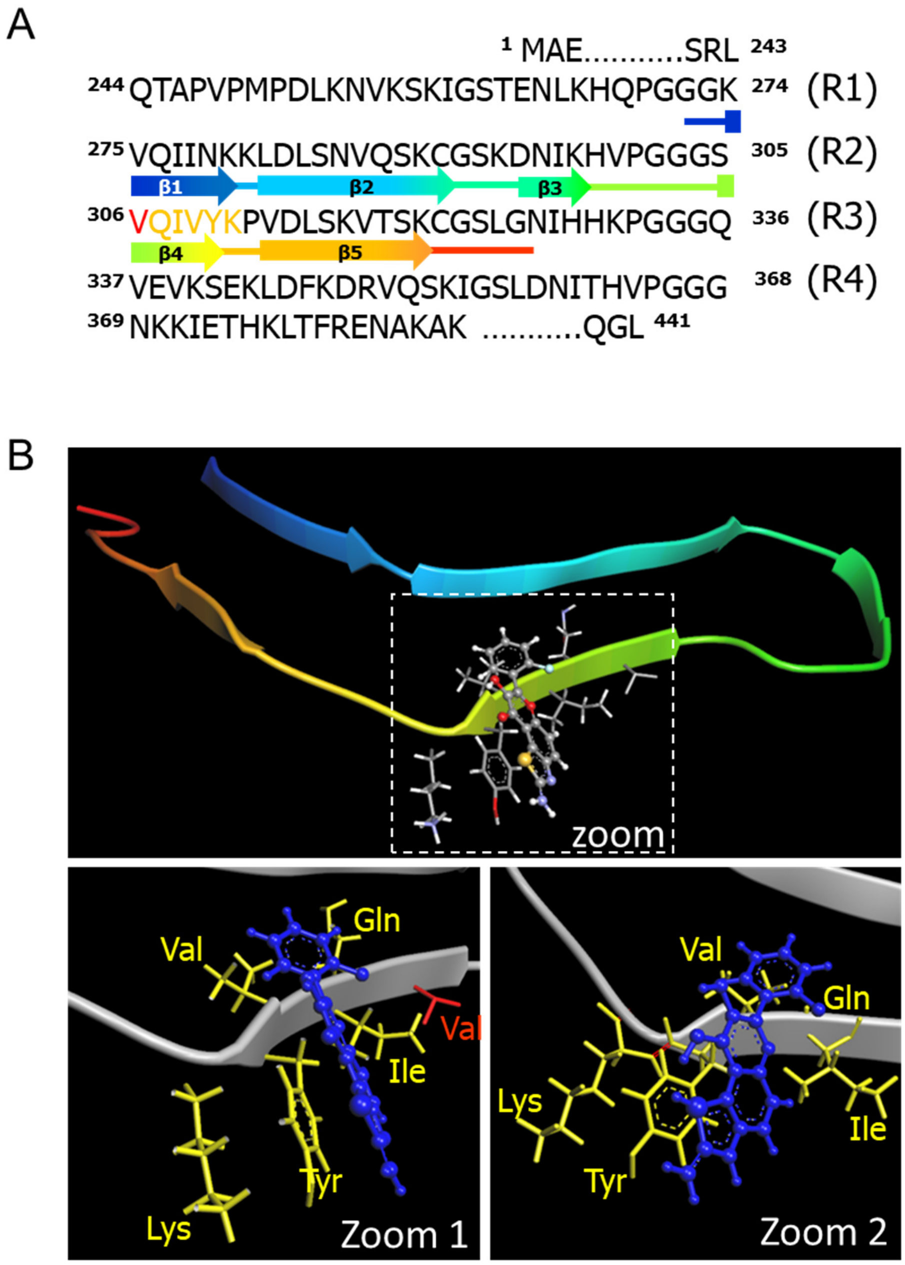

Supplementary Figure S7. In silico model of interaction between thiazoloflavonoid derivatives and Tau filaments. (A) β-strands and loop regions in the filaments are shown in different colors below the primary se-quence of the microtubule-binding repeats (R1-R4); the VQIVYK sequence (R3) is marked in yellow. (B) Representation of the secondary structure element of heparin-induced Tau snake filament (Zhang et al, 2019) binding to compound 9; (upper panel) overview of binding site of 9 with the VQIVYK sequence (R3) in Tau protein; (lower panels) two zoomed views highlight position of 9 (in blue) near to the tyrosine residue (in yellow) of β4 sheet structure of Tau filament (in gray): the planar flavonoid structure of ring A and C fused to thiazole group is closed to tyrosine residue, and the fluorinated ring B is embedded between two successive rungs (not shown here), resulting in potent steric hindrance for Tau assembly into filament.
